# Redox Isomerism in Ethynyl-Bridged Triazatruxene-Diarylmethylium Dyads

**DOI:** 10.1021/acs.joc.5c01973

**Published:** 2025-11-04

**Authors:** Lars Vogelsang, Felix Kuschel, Anja Rehse, Michael Linseis, Rainer F. Winter

**Affiliations:** Fachbereich Chemie, Universität Konstanz, Universitätsstraße 10, 78464 Konstanz, Germany

## Abstract

We present two triazatruxene-triarylmethylium TAT-Tr^+^ donor–acceptor dyads **1** ^**+**^ and **2** ^**+**^ with either 4-CF_3_- (**1** ^**+**^) or 4-F-substituted phenyl rings (**2** ^**+**^) at the tritylium (Tr^+^) site. In spite of rather large differences between the redox potentials for TAT oxidation and Tr^+^ reduction, the diamagnetic TAT-Tr^+^ forms of these dyads coexist with their paramagnetic TAT^+•^-Tr^•^ valence tautomers with one unpaired spin at every redox site. The major quantity of the diradical isomers is trapped as dimers with concomitant loss of the unpaired spin density of the Tr^•^ entity. The dimers were also observed by cyclic and square wave voltammetry and form readily by one-electron reduction of the dyads to the corresponding trityl radical. Dimerization of the neutral radicals occurs at a much faster rate than dissociation of their two-electron oxidized forms, indicating hysteretic behavior.

## Graphical abstract

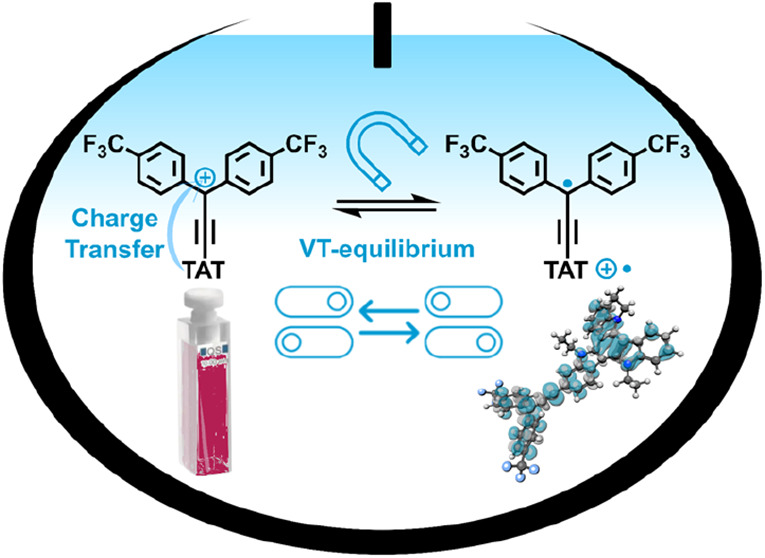

## Introduction

Molecular electronics is a widely studied research area aimed at utilizing individual molecules as components of integrated circuits as a direction toward ultimate device miniaturization.^1–4^ Switches that can reversibly toggle between different states, and whose actual state can be read out without inflicting a state change, are indispensable functional units of such circuits. Present realizations of molecules that can serve this purpose capitalize on different spin^5–7^ or charge states,^8^ distinguishable tautomeric forms that interconvert by intramolecular proton transfer,^9^ different conformations, such as rotamers,^10–12^ or molecules that can undergo reversible formation or breaking of covalent bonds.^13,14^ Another potential design of a molecular switch relies on electronic bistability, i. e. on coexisting pairs of redox isomers, so-called valence tautomers (VTs),^15–17^ that interconvert by intramolecular electron transfer (IET). The design of such molecules relies on the presence of two chemically distinct, redox-active constituents that oxidize or reduce at similar potentials and are electronically mutually insulated.^18,19^ Dyads composed of a ferrocenyl (Fc) electron donor and a perchlorotriphenylmethyl (PTM) electron acceptor as pioneered by the Veciana group as well as some pyrrole- or carbazole-tris­(2,4,6-trichlorophenyl)­methyl (TTM) radicals from the Julia group rank among the most prominent examples of such VTs (see Figure 1a).^20–23^ Thermal equilibria between the Fc-PTM^•^ and the Fc^+•^-PTM^–^ forms were studied by EPR, optical and Mößbauer spectroscopy.^20–22,24–26^ This concept was later expanded to dyads with tetrathiafulvalene (Figure 1b),^25,27–30^ oligothienylenevinylene or carbazole-based^30,31^ donors. In all these systems, both VTs have one unpaired spin which is either localized at the triarylmethyl (donor-PTM^•^/TTM^•^ form) or the donor constituent (donor^+•^-PTM^–^/TTM^–^ isomer).

**Figure 1:**
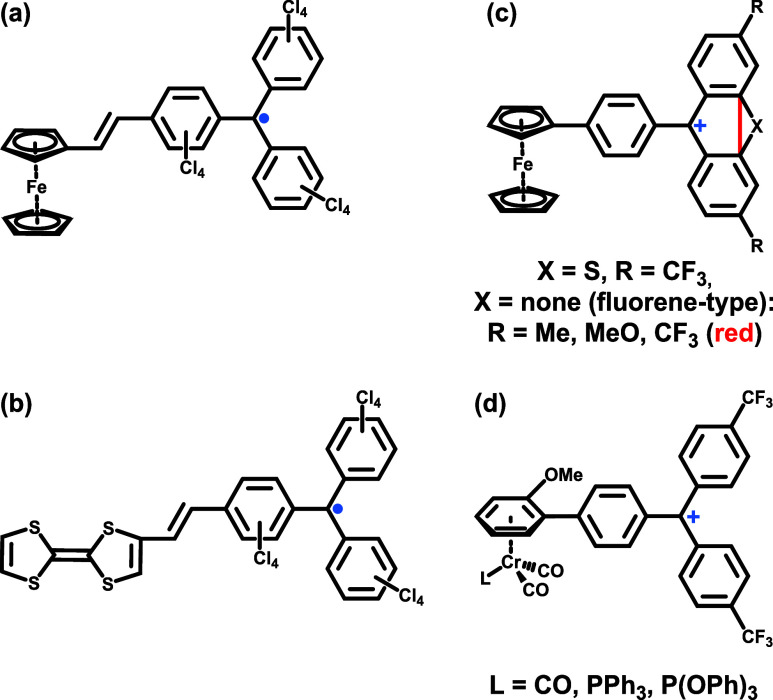
Overview over some donor–acceptor (D-A) dyads that give rise to valence tautomerism. (a) Ferrocenyl-PTM^•^; (b) tetrathiafulvalene-PTM^•^; (c) ferrocenyl-thioxanthylium and -fluorenylium ions; (d) chromium half sandwich-tritylium dyads.

We present two triazatruxene-triarylmethylium TAT-Tr^+^ donor–acceptor dyads **1** ^**+**^ and **2** ^**+**^ with either 4-CF_3_- (**1** ^**+**^) or 4-F-substituted phenyl rings (**2** ^**+**^) at the tritylium (Tr^+^) site. In spite of rather large differences between the redox potentials for TAT oxidation and Tr^+^ reduction, the diamagnetic TAT-Tr^+^ forms of these dyads coexist with their paramagnetic TAT^+•^-Tr^•^ valence tautomers with one unpaired spin at every redox site. The major quantity of the diradical isomers is trapped as dimers with concomitant loss of the unpaired spin density of the Tr^•^ entity. The dimers were also observed by cyclic and square wave voltammetry and form readily by one-electron reduction of the dyads to the corresponding trityl radical. Dimerization of the neutral radicals occurs at a much faster rate than dissociation of their two-electron oxidized forms, indicating hysteretic behavior.

Conceptually even more intriguing are pairs of VTs where the two isomers differ in the number of unpaired spins. Examples of such systems are Co complexes of redox-active dioxolene ligands that can switch between low-spin Co­(III) catecholate and high-spin Co­(II) semiquinonate forms, with the Co­(II) ion and the one-electron oxidized ligand as paramagnetic centers.^11–16,32,33^

Previous work of our group has revolved around dyads composed of ferrocene or chromium η^6^-benzene half-sandwich complex donors (CDs) and triarylmethylium (tritylium, Tr^+^) acceptors (see Figure 1c,d), which can switch between a diamagnetic, closed-shell CD-C­(Aryl)_3_^+^ and an open-shell CD^+•^-C^•^(Aryl)_3_ state with one unpaired spin at each of the two redox-active constituents.^34–38^ Like Veciana′s and Julia′s systems, they profit from the comparably high thermal and chemical stabilities of tritylium ions and trityl radicals.^39–47^ In the present work, we use triazatruxenes (TATs, Figure 2) as the donors of such dyads. Triazatruxenes are electron-rich, planarized triarylamines which are readily oxidized at modest potentials to persistent radical cations^48–50^ and dications.^51–53^ Both cations are resonance-stabilized, intrinsically delocalized mixed-valent systems of Class III according to the classification scheme of Robin and Day.^54,55^ Trisubstitution at either the *N*-atoms or the 2- or 3-positions, the latter differing in the extent of π-conjugation with the triindole core, offers ample opportunities for further modification (Figure 2).^56–60^ TATs have important appearances in the field of organic electronic devices,^61–65^ e.g. as organic field-effect transistors (OFETs),^61,66–68^ in organic photovoltaics (OPVs),^69,70^ and in organic light-emitting diodes (OLEDs).^71–73^

**Figure 2:**
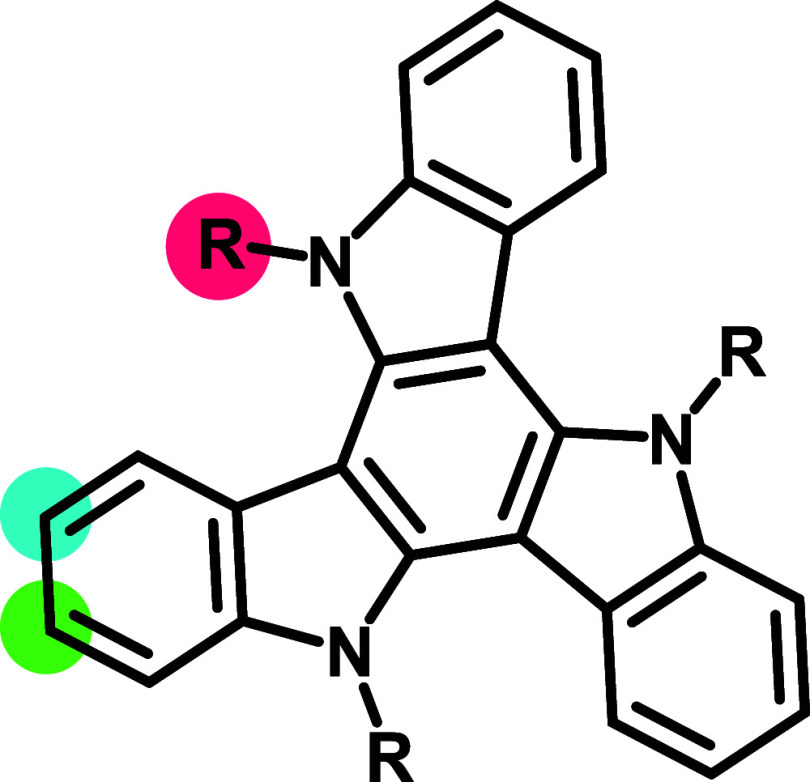
Skeletal formula of the TAT scaffold with the positions marked, which can be used for modification: the amino substituent in red, the 3-position in blue, and the 2-position in green.

In the Fc-Tr^+^/Fc^+•^-Tr^•^ dyads and the respective chromium half-sandwich complexes, the metal-centered spin escapes detection above 10 or 77 K, respectively, due to rapid relaxation.^74–79^ In contrast, the open-shell one-electron oxidized or reduced forms of the present dyads, i. e. TAT^+•^ and Tr^•^, are both EPR active at room temperature,^39,44,54,80,81^ rendering their diradical VTs easily detectable.^26,27,82^ This prompted us to prepare and investigate two such dyads with electron-deficient tritylium cations. The results of our electrochemical and spectroscopic investigations are detailed below.

## Results and Discussion

### Synthesis, Spectroscopic Characterization and Redox Properties

Our synthetic approach to TAT-C≡C–C^+^-(C_6_H_4_-4-R)_2_ dyads (R = CF_3_, **1** ^**+**^; R = F, **2** ^**+**^), (further on simplified as TAT-Tr^+^) involves deprotonation of the TAT monoalkyne **2-A**_**1**_ **-** ^**Et**^ **TAT** ^51^ with ^*n*^BuLi and the nucleophilic attack of the resulting acetylide on the carbonyl C atom of electron-poor benzophenones (4-R-C_6_H_4_)_2_C=O to provide carbinols **1-OH** and **2-OH** (Figure 3). Acceptor substitution on the benzophenone is, on the one hand, required to bring the reduction potential of the later tritylium (Tr^+^) unit reasonably close to that of TAT oxidation. On the other hand, the carbonyl C atom of benzophenone and benzaldehyde proved to be too little electrophilic to react with the TAT acetylide. *para*-Substitution at the phenyl rings is also instrumental in blocking dimerization of trityl radicals to so-called Jacobsen-Nauta dimers (see Figure S38 of the Supporting Information).^83^

**Figure 3:**
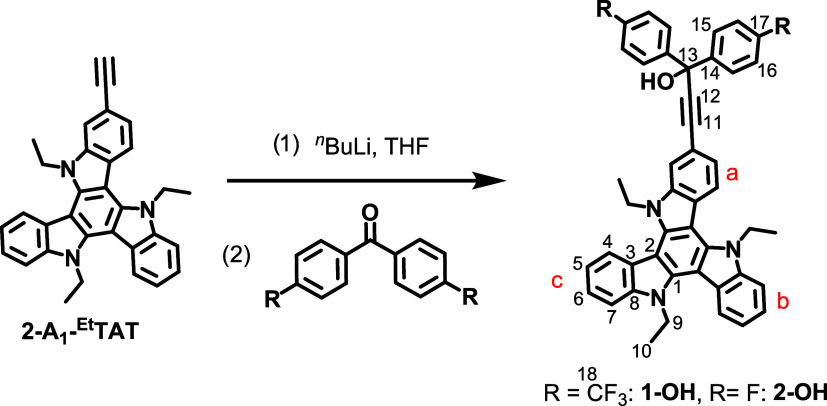
Synthesis of carbinols **1-OH** (R = CF_3_) and **2-OH** (R = F) with the numbering of the carbon atoms.

^1^H NMR-spectra of **1-OH** and **2-OH** and of the starting materials as well as ^13^C­{^1^H}-NMR-, IR- and mass spectra of the final products are provided as Figures S1 to S15 of the Supporting Information. The absence of the ethynyl proton resonance and of the ≡C–H stretch of the terminal alkyne^51^ as well as of the C=O stretching vibration of the benzophenone in IR spectra^84,85^ together with the new O–H proton resonance (**1-OH**: δ = 3.12 ppm, **2-OH**: δ = 2.98 ppm) demonstrate successful conversion. Easy protonation and dehydration of the carbinols to the desired TAT-Tr^+^ dyads is already evident from their mass spectra, which feature the molecular ion peak of the tritylium species **1** ^**+**^ and **2** ^**+**^ together with those of the {**1-OH**+H}^+^ and {**2-OH**+H}^+^ ions. Synthetically, this conversion was quantitatively achieved by careful addition of stoichiometric amounts of Brookhart’s acid, H­(OEt_2_)^+^ [BAr^F24^]^−^ ([BAr^F24^]^−^ = [B­{C_6_H_3_(CF_3_)_2_-3,5}_4_]^−^, Figure 4).^86^ Successful acid-induced dehydration is indicated by an immediate color change from light orange to intense pink (**1** ^**+**^) or red-brown (**2** ^**+**^), which is typical of such donor-tritylium dyads.^34,38,87,88^ In previous work, the very weakly coordinating [BAr^F24^]^−^ anion was found to be instrumental in chemically stabilizing such highly electrophilic Tr^+^ cations.^34,35,37,38,87,88^ Solutions of the cationic dyads were NMR silent, which already indicates paramagnetism of the samples and the presence of substantial quantities of the diradical VTs.

**Figure 4:**
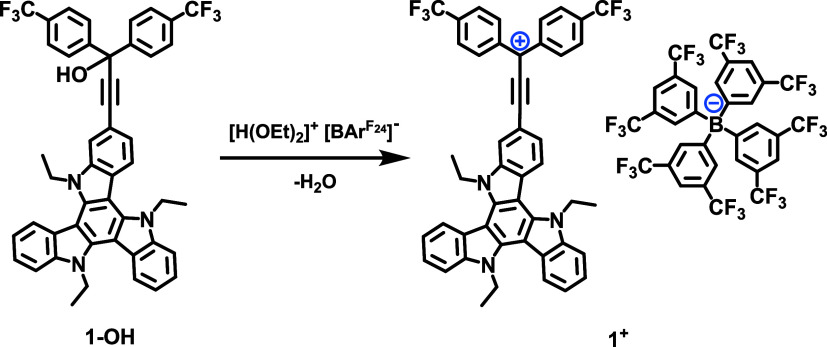
Preparation of the ethynylogous TAT-tritylium dyads **1** ^**+**^ and **2** ^**+**^ in dry CH_2_Cl_2_ as the solvent.

Both carbinols exhibit a chemically reversible TAT-centered oxidation at a half-wave potential *E*_1/2_^0/+^ of 355 mV for **1-OH** or of 367 mV for **2-OH**, similar to alkyne **2-A**_**1**_ **-** ^**Et**^ **TAT** (*E*_1/2_ = 331 mV, see Table 1 and Figure S16).

**Table 1:** Half-Wave Potentials of the Redox Processes of **1-OH** and **2-OH** and Related TATs in CH_2_Cl_2_/NBu_4_^+^ [BAr^F24^]^−^ (0.04 M) (^a^), or in CH_2_Cl_2_/NBu_4_^+^ PF_6_^–^ (0.06 M) (^b^)

	***E***_**1/2**_^**TAT/TAT+**^	***E***_**1/2**_^**TAT+/TAT2+**^	***ΔE***_**1/2**_
^**Et**^ **TAT** ^**b**^	298	914	616
**2-Br**_**3**_ **-** ^**Et**^ **TAT** ^**b**^	481	1011	530
**2-A**_**1**_ **-** ^**Et**^ **TAT** ^**b**^	331	897	566
**1-OH** ^**a**,^ ^a^	355		
**2-OH** ^**a**^	367	1153	786

a Reversible reduction at *E*_1/2_ = −1985 mV (see Figure S20 of the Supporting Information).

The expected, second oxidation of the TAT unit of **2-OH** is observed at unusually positive potential (see Table 1). In the case of **1-OH**, the strongly electron-withdrawing CF_3_ substituents at the remote diarylmethanol entity shift this process to outside the anodic breakdown limit of the CH_2_Cl_2_/NBu_4_^+^ [BAr^F24^]^−^ electrolyte while giving rise to a reversible reduction wave at −1985 mV. No such process is detected for **2-OH** and **2-A**_**1**_ **-** ^**Et**^ **TAT**.

Voltammetric investigations on freshly prepared solutions of cations **1** ^**+**^ and **2** ^**+**^ in the CH_2_Cl_2_/NBu_4_^+^ [BAr^F24^]^−^ (0.04 M) electrolyte revealed a rather intricate yet overall similar redox behavior. In first instance, the positive charge at the methylium center shifts the redox wave for TAT oxidation, process A′/A in Figure 5, by 434 (**1** ^**+**^) or 263 mV (**2** ^**+**^) anodicallyy (i.e., to a higher potential), to *E*_1/2_ = 789 mV in **1** ^**+**^, or to 630 mV in **2** ^**+**^. The ordering of the half-wave potentials thus follows the Hammett σ_p_ parameter of the *para* substituents at the diarylmethylium moiety (σ_p_(CF_3_) = 0.54, σ_p_(F) = 0.06).^89,90^ The large impact of the diarylmethyl substituents on the TAT oxidation potential is a token of uninterrupted π-conjugation within the cations. This contrasts with the carbinols, where the tetrahedral, sp^3^-hybridized C atom decouples the TAT donor from the diarylmethanol entity.

**Figure 5:**
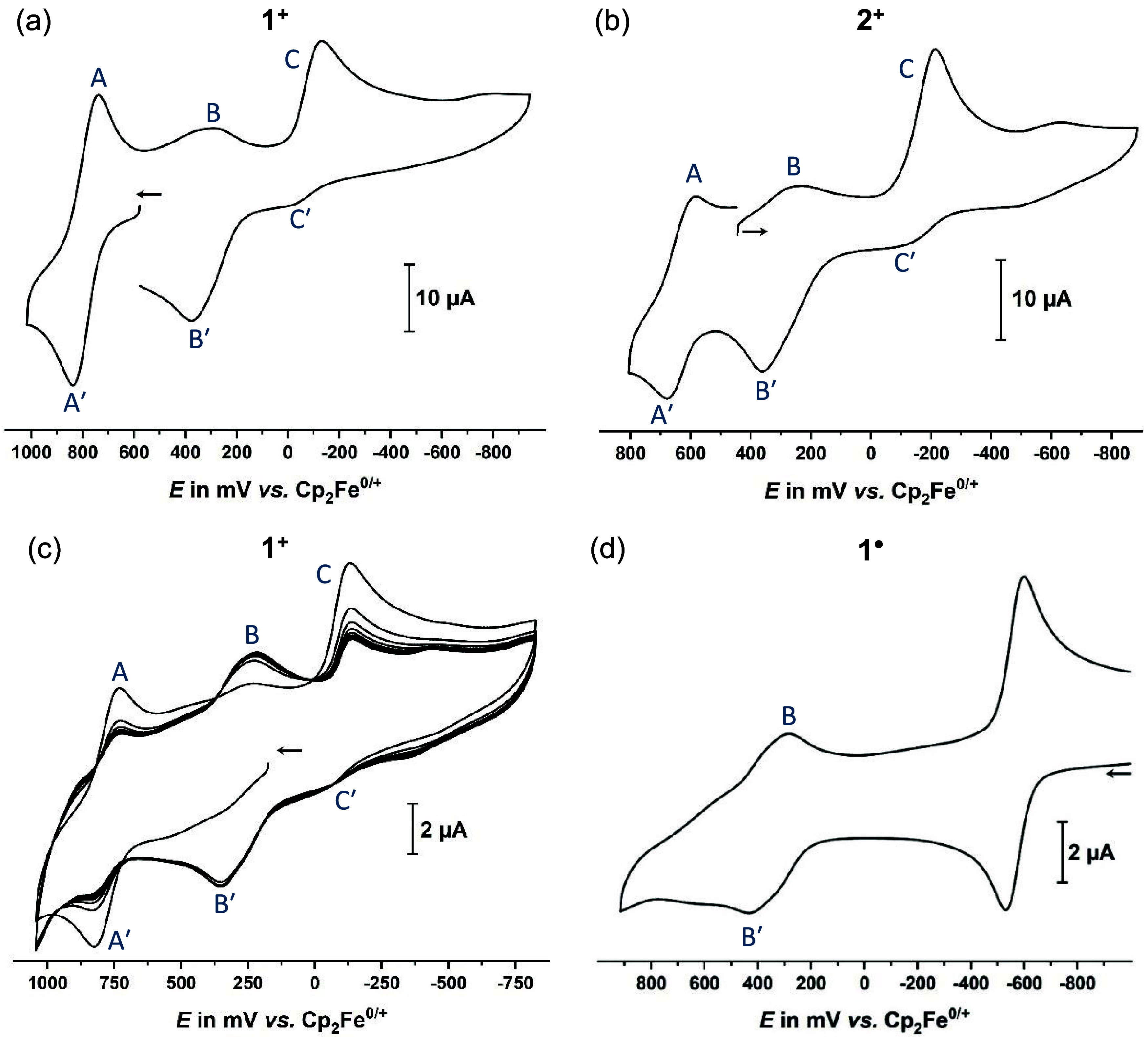
(a) Cyclic voltammogram of **1** ^**+**^ with the sweep initiated negative of the TAT oxidation (process A′/A, *c* = 0.7 mM, starting at 580 mV in oxidative direction, switching at 1030 mV and at −950 mV). (b) Cyclic voltammogram of **2** ^**+**^ starting slightly positive of wave B/B′ assigned to the reduction of the valence tautomeric, diradical dimer **2** ^**+•**^^–^ **2** ^**+•**^ with the initial (forward) sweep directed to past the reduction wave C/C′ (*c* = 0.6 mM, starting at 440 mV in reductive direction, switching at −880 mV and at 810 mV). (c) Cyclic voltammogram of **1** ^**+**^ with the sweep initiated negative of the wave B′/B assigned to the oxidation of the valence tautomeric diradical dimer **1** ^**+•**^ **-1** ^**+•**^ and the initial scan taken to past wave A′/A. After the initial scan, a total of 20 consecutive cycles were recorded (*c* = 0.4 mM, starting at 180 mV in oxidative direction, switching at 1040 mV and at −830 mV). (d) Cyclic voltammogram of chemically reduced **1** ^**+**^ showing the complete loss of wave C′/C and conversion to the putative dimer, which gives rise to wave B′/B (*c* = 0.9 mM, starting at −980 mV in oxidative direction, switching at 920 mV; note that the prominent wave at −550 mV is due to the decamethylferrocene/-ferrocenium redox couple, used as the chemical reductant). All measurements were conducted in CH_2_Cl_2_/NBu_4_^+^ [BAr^F24^]^−^ (0.04 M), at *v* = 600 mV/s and at r. t. CVs are plotted using the polarographic convention.

The same potential ordering applies to the reduction of the tritylium-like moiety, denoted as wave C/C′, which is observed as a chemically almost irreversible process at a cathodic peak potential of −96 mV (**1** ^**+/•**^) or −213 mV (**2** ^**+**/**•**^) (see Figure 5). In accordance with the electron-donating capabilities of the 2-ethynyl-^Et^TAT entity, the reduction potentials are shifted to more negative values when compared to the corresponding tritylium ions (4-R-C_6_H_4_)_3_C^+^ (R = CF_3_: *E*_1/2_^+/•^ = 290 mV;^91^ R = F: *E*_1/2_^+/•^ = ca. −150 mV^92^). Similar findings were reported for other dyads with the same diarylmethylium electron acceptors and ferrocene or {(η^6^-C_6_H_3_(OMe)­(2-Ph))­Cr­(CO)_2_(PPh_3_)} as the donor,^34,37,88,93,94^ or for dyads, where an appended donor hampers reduction of the TTM or the PTM radical, while it facilitates oxidation to the corresponding cation.^20–30,82,95,96^

Rather than showing a counter peak C′ attributable to the back-oxidation of the neutral radical to the methylium cation, a new redox process involving peaks B′/B, located at 318 mV (**1**) or 280 mV (**2**) is observed after traversing peak C. As shown in Figure 5, this process shows signs of small redox splittings for both, the forward peak B′ as well as the reverse peak B (for further voltammograms recorded at different sweep rates and for square wave voltammograms, see Figures S23 to S29 of the Supporting Information). It hence likely originates from a two-step electron transfer process that involves two identical, spatially separated and weakly interacting redox centers. Similar behavior was formerly observed for reduced ferrocenyl-tritylium and -fluorenylium ions and was ascribed to the stepwise oxidation of the donor constituents of dimers of the electrogenerated radicals.^94^ We hence assign wave B′/B to TAT oxidations in dimers **1–1** and **2–2** which form after reduction of the cations in peak C.

To shed more light on the fate of the neutral radicals, we chemically generated them inside the electrochemical cell by reducing methylium species **1** ^**+**^ and **2** ^**+**^ with excess decamethylferrocene, Cp*_2_Fe, as a sufficiently strong reductant (*E*_1/2_^+/0^ = −550 mV). Voltammograms recorded from these solutions, as exemplified for compound **1** ^**+**^ in Figure 5d, indeed show exclusively wave B′/B, now as a chemically reversible redox couple (see also Figures S30 and S31 of the Supporting Information). The same holds for chemically generated **2** ^**•**^ as shown in Figures S32 and S33 of the Supporting Information. Likewise, continuous cycling to past oxidation peak A′ and reduction peak C causes a gradual growth of wave B′/B at the expense of the redox couples A′/A and C/C′ (see Figure 5c). All these findings indicate that dimerization of the neutral trityl-type radicals occurs at a significantly faster rate than dissociation of the oxidized dimers.

Importantly, in voltammetric scans of **1** ^**+**^ and **2** ^**+**^ that are initiated slightly positive or negative of the redox couple B′/B, yet without prior scanning through wave C/C′, the latter peaks are still discernible (see Figure 5a,b, the initial scan in Figure 5c, as well as Figures S23 to S29 of the Supporting Information); they however appear with distinctly lower intensity than after passing through peak C, as is best seen in Figure 5c. The same applies to **2** ^**+**^ (Figure 5b). This brings us to the conclusion that dimers are already present without prior reduction of the tritylium cations to their neutral forms, meaning that monomeric cations **1** ^**+**^ and **2** ^**+**^ equilibrate with persistent dimers **1** ^**+•**^ **–1** ^**+•**^ and **2** ^**+•**^ **–** **2** ^**+•**^, respectively. Intriguingly, these dimers must derive from the valence tautomeric, diradical forms TAT^+•^-Tr^•^ of cations **1** ^**+**^ and **2** ^**+**^, which stabilize as dimers. This is very remarkable, as the differences in the half-wave potentials for their TAT^0/+•^ and Tr^+/•^ redox couples of 920 mV (**1** ^**+**^) and 817 mV (**2** ^**+**^), respectively, translate into Δ*G* values of 88.8 (**1** ^**+**^) or 78.8 (**2** ^**+**^) kJ/mol for the paramagnetic VTs. We thus conclude that dimerization, likely via formation of a direct C–C bond, contributes significantly to stabilizing the diradical VTs, thereby rendering them competitive with their closed-shell monomers. Figure 6 summarizes the redox processes and proposed equilibria of compounds **1** ^***n*+**^ and **2** ^***n*+**^ in their different redox states, while pertinent electrochemistry data are provided in Table 2. We note again the close relationship to ferrocenyl- and TTF-PTM^•^ as well as to pyrrole- and carbazole-TTM^•^ radicals, which were found to equilibrate with their donor^+•^-PTM^–^ or donor^+•^-TTM^–^ VTs. In the latter systems, the chloro substituents at the triarylmethyl unit however suppress dimerization.^24,26–29,82^

**Figure 6:**
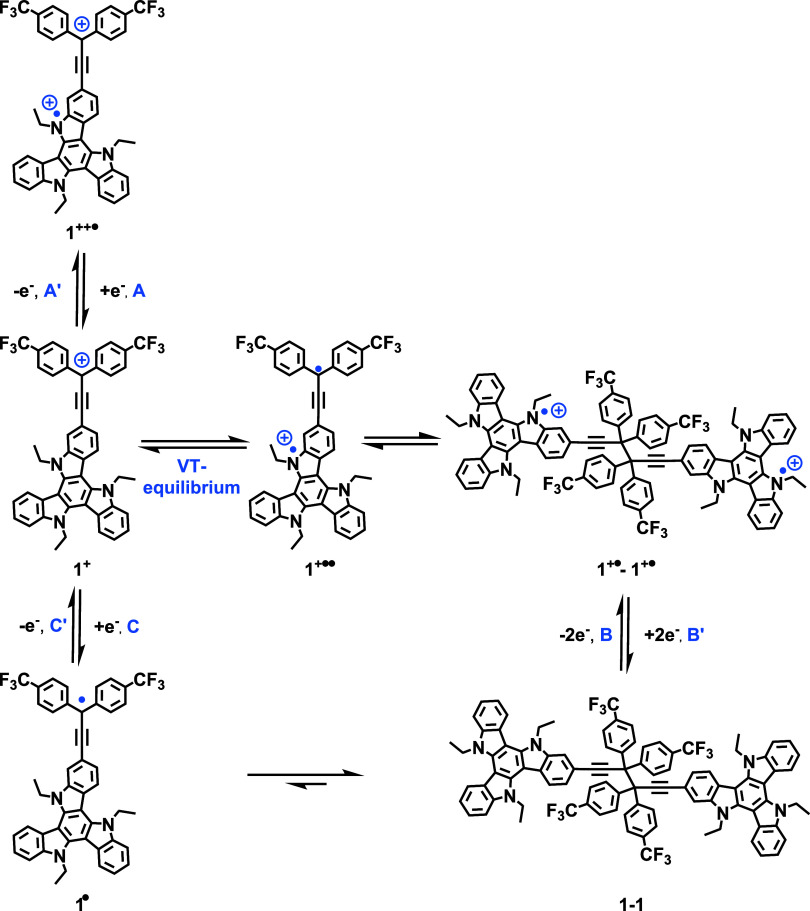
Simplified scheme of the redox processes and the proposed monomer/dimer equilibria for cations **1** ^**+**^ and **2** ^**+**^ at the example of **1** ^**+**^. Note that reduction of **1** ^**+•**^ **–1** ^**+•**^ to **1**-**1** and the oxidation of neutral **1**–**1** to **1** ^**+•**^ **–1** ^**+•**^ occur as two consecutive, closely spaced one-electron processes.

**Table 2:** Potentials of the Redox Processes Observed for **1^+^** and **2^+^** in mV in CH_2_Cl_2_/NBu_4_^+^ [BAr^F24^]^−^ (0.04 M) at r. t. and at a Scan Rate of 600 mV/s^a^

	A′/A	B′/B	C
**1** ^**+**^	789	318 (278, 358)	–96
**2** ^**+**^	630	280 (239, 321)	–213

a The redox processes are defined as given in Figures 5 and 6. The individual potential of the two steps of process B′/B is given using the broadening of the square wave peaks of B′/B when compared to the TAT oxidation A′/A in Figures S23 and S24 in oxidative direction.^97^ Due to chemical irreversibility of the redox couple C′/C, only the potential of the reductive peak C is provided.

### Valence Tautomerism Probed Through EPR Spectroscopy

The conjecture of coexisting diamagnetic cations **1** ^**+**^ and **2** ^**+**^ and their valence tautomeric, diradical isomers **1** ^**+•**^ **–** **1** ^**+•**^ and **2** ^**+•**^ **–** **2** ^**+•**^ implies that samples of **1** ^**+**^ and **2** ^**+**^ should exhibit the EPR resonance of a TAT^+•^ radical cation. Solutions of **1** ^**+**^ and **2** ^**+**^ in CH_2_Cl_2_ are indeed EPR active. They both provide an isotropic EPR resonance at a *g*-value of 2.003 without any observable hyperfine splitting (hfs) to nitrogen or hydrogen atoms as shown in Figure 7, in good match with the pristine TAT^+•^ cation.^54^ The lack of resolved hfs is due to extensive delocalization of the unpaired spin density over the entire TAT backbone, while the absence of hfs to the F nuclei at the trityl constituent precludes the presence of appreciable unpaired spin density at the diarylmethyl site. This matches with the notion that the vast majority of the paramagnetic VT exists as dimers.

**Figure 7:**
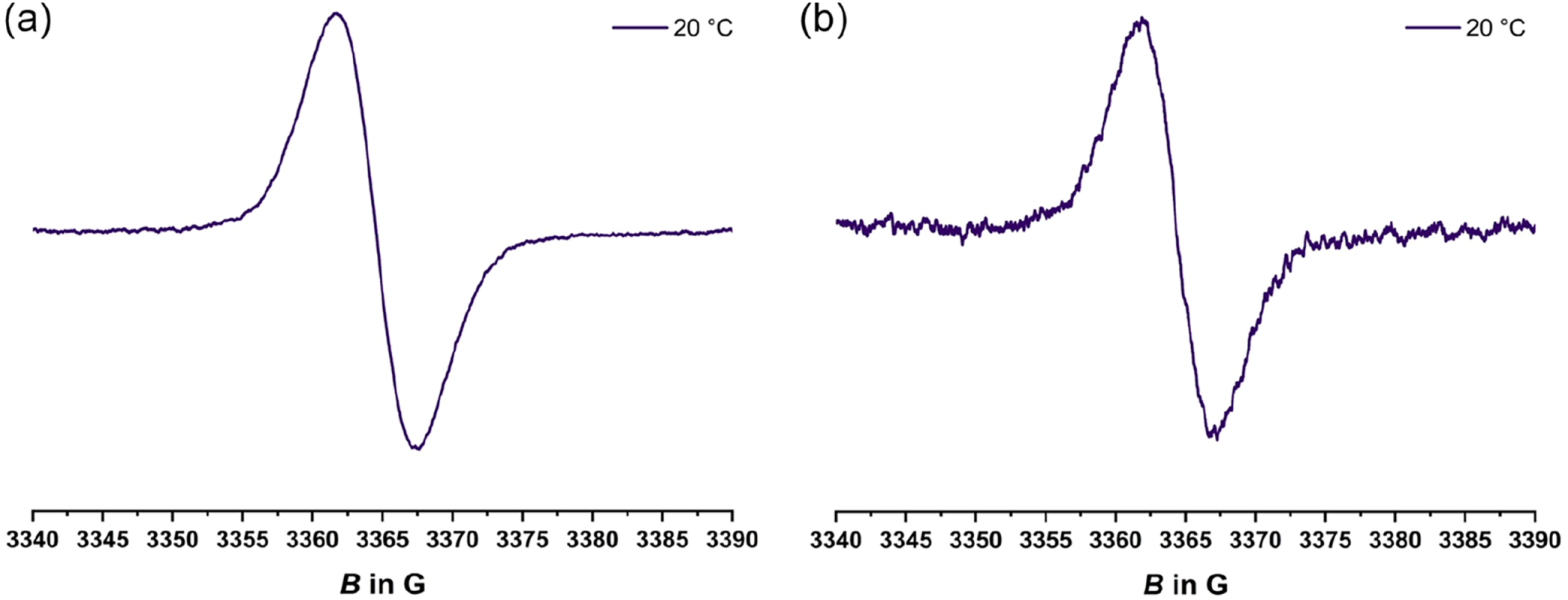
EPR spectra of (a) a 1.0 mM solution of cation **1** ^**+**^ and of (b) a 1.7 mM solution of cation **2** ^**+**^ at 20 °C (purple) in CH_2_Cl_2_ at r.t.

Although they are likewise dominantly present in the form of dimers, monomeric radicals **1** ^**•**^ and **2** ^•^, generated by reduction of **1** ^**+**^ and **2** ^**+**^ with excess decamethylferrocene, are nevertheless readily identified by EPR spectroscopy. Resolved hfs to six or two equivalent F nuclei and, in the case of **1^•^**, additional hfs to eight aryl protons (Figures 8 and S39 of the SI) clearly identify the respective trityl-like entity as the spin-bearing site. The hfs constants retrieved from digital simulations^98^ or the experimental spectra closely resemble those of the corresponding trityl radicals Ph­(C_6_H_4_-4-R)_2_C^•^ and (C_6_H_4_-4-R)_3_C^•^ in the literature.^39,43^ Relevant EPR data of all discussed compounds and the corresponding trityl derivatives are provided in Table 3. Double integration against calibrated samples of the DPPH^•^ standard according to a literature method^87^ provided us with an estimate of the amount of nondimerized radical **1** ^**•**^ present at 20 °C of ca. 10% of the nominal concentration of **1^•^**, and of only ca. 1% for **2** ^**•**^. This is internally consistent with the small intensity of the anodic return peak C′ following reduction and the prominent wave B′/B ascribed to the corresponding dimer **1–1** or **2–2**. One should note here that the TAT^+•^ and trityl radicals of the present study have the same *g* values. The EPR resonance observed for the cationic species is mainly that of TAT^+•^, as the latter resonance is present in the dicationic dimers, which is the major form, in which the cationic VTs exist, and in the minor quantities of monomeric TAT^+•^–Tr^•^ diradicals, whereas the Tr^•^ resonance is confined to the monomers. The reduced forms however only exhibit the Tr^•^-derived spin. This also explains why hfs to F nuclei are resolved for the neutral radicals, but not for the cations.

**Figure 8:**
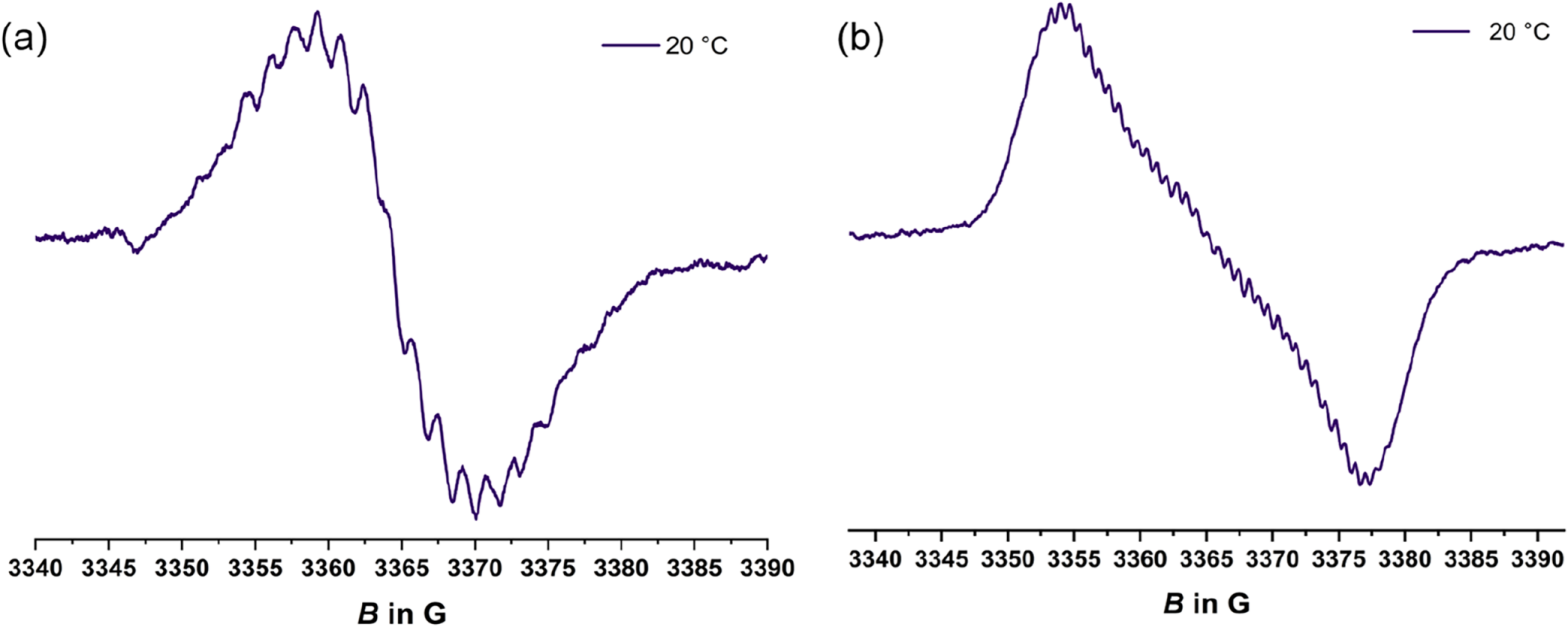
EPR spectra of (a) a 1.0 mM solution of **1** ^•^ (*A*(^19^F) = 4.10 G, *A*(^1^H_*o*_) = 3.0 G, *A*(^1^H_m_) = 0.6 G) and (b) a 1.7 mM solution of **2** ^•^ (*A* = 0.7 G; *A*(^19^F) = 6.8 G) in CH_2_Cl_2_ at r.t.

**Table 3:** EPR-Data of **1^+^**, **2^+^**, **1** ^•^, **2^•^** and Different Relevant Radicals from Literature in CH_2_Cl_2_ at r.t^a^

	*g*-value	hfs
**1^+^**	2.003	
**2** ^**+**^	2.003	
**1^•^** ^ a^	2.003	*A*(^19^F) = 4.10 G, *A*(^1^H_*o*_) = 3.0 G, *A*(^1^H_*m*_) = 0.6 G
		
		
2^•^ ^b^	2.003	*A*(^19^F) = 6.79 G
(4-FC_6_H_4_)_3_C^•^ ^81,99^	2.003	*A*(^19^F) = 6.50 G, *A*(^1^H_*o*_) = 2.70 G, *A*(^1^H_m_) = 1.13 G
(4-(CF_3_)-C_6_H_4_)_3_C^•^ ^81,91^	2.002	*A*(^19^F) = 4.00 G, *A*(^1^H_*o*_) = 2.58 G, *A*(^1^H_m_) = 1.15 G
Ph-(4-(CF_3_)-C_6_H_4_)_2_C^•^ ^39^	2.004	*A*(^19^F) = 4.20 G
^Et^ **TAT** ^+•^ ^100^	2.003	

a Obtained from digital simulation.

b Taken from the experimental spectrum.

The amounts of radical species contained in samples of **1** ^**+**^ and **2** ^**+**^ were estimated by quantitative EPR spectroscopy, using the stable DPPH^•^ radical as calibrant. Our estimate of the content diradical VT and its dimer is based on the simplifying assumption that the neutral radical and the cationic diradical dimerize to an identical extent (see the Supporting Information for details). The results of this analysis are compiled in Table 4.

**Table 4:** Amounts of Dimerization of **1^•^**/**2** ^•^ Obtained from Comparison with a DPPH^•^ Reference and Estimated Amount of Diradical VT Present in **1^+^**/**2^+^**

	monomeric radical **1** ^**•**^ or **2** ^**•**^ at r. t. in %	fraction of **1** ^**+••**^ */* **2** ^**+••**^ present in **1** ^**+**^/**2** ^**+**^ in %^87^
**1** ^**+/•**^	10	4.6
**2** ^**+/•**^	1	0.8

The rather small free radical contents for solutions of **1** ^**+**^ and **2** ^**+**^ as determined by EPR spectroscopy and the substantial differences between the two compounds need to be reconciled with the more substantial and overall very similar peak currents for the waves B/B′ assigned to their dimers that were observed in our cyclic voltammetry studies. The only plausible explanation we can think of is that the VT equilibrium for compound **2** ^**+**^ establishes on a faster rate than for **1** ^**+**^ and that equilibration between monomer and dimer is likewise fast. This, in concert, would aid to replenish dimer **2** ^**+•**^ **–2** ^**+•**^ as it is consumed in the diffusion layer near the electrode surface at a rate that keeps pace with the electrochemical experiment (see also Figures S25 to S29 of the Supporting Information).

As shown above, both types of radical species engage in monomer/dimer equilibria. For enthalpic and entropic reasons, dimerization will be more favored at lower temperatures *T.* One therefore expects that the EPR signal intensity of both types of radicals, the neutral and cationic forms, decrease on cooling, due to removing all of the unpaired spin density per two molecules undergoing dimerization (neutral forms), or the trityl-based spin of the diradical VTs of the cations, leaving the TAT^+•^ spin unaltered. We therefore monitored *T*-dependent EPR spectra using dichloromethane solutions of **1** ^**+**^ and **1** ^**•**^ and identical instrument settings. Dichloromethane was the only solvent where both redox forms were sufficiently stable and soluble over a wider temperature range that we could identify. The results of this study are provided in Figure 9. We indeed observed that the EPR signal intensity drops steadily with decreasing temperature *T*, contrary to the Boltzmann behavior expected otherwise. The general behavior thus fully agrees with the *T*-dependent shifting of the monomer/dimer equilibria. We nevertheless note that dichloromethane is known as a “lossy” EPR solvent, which might also contribute to the observed behavior.^101,102^

**Figure 9:**
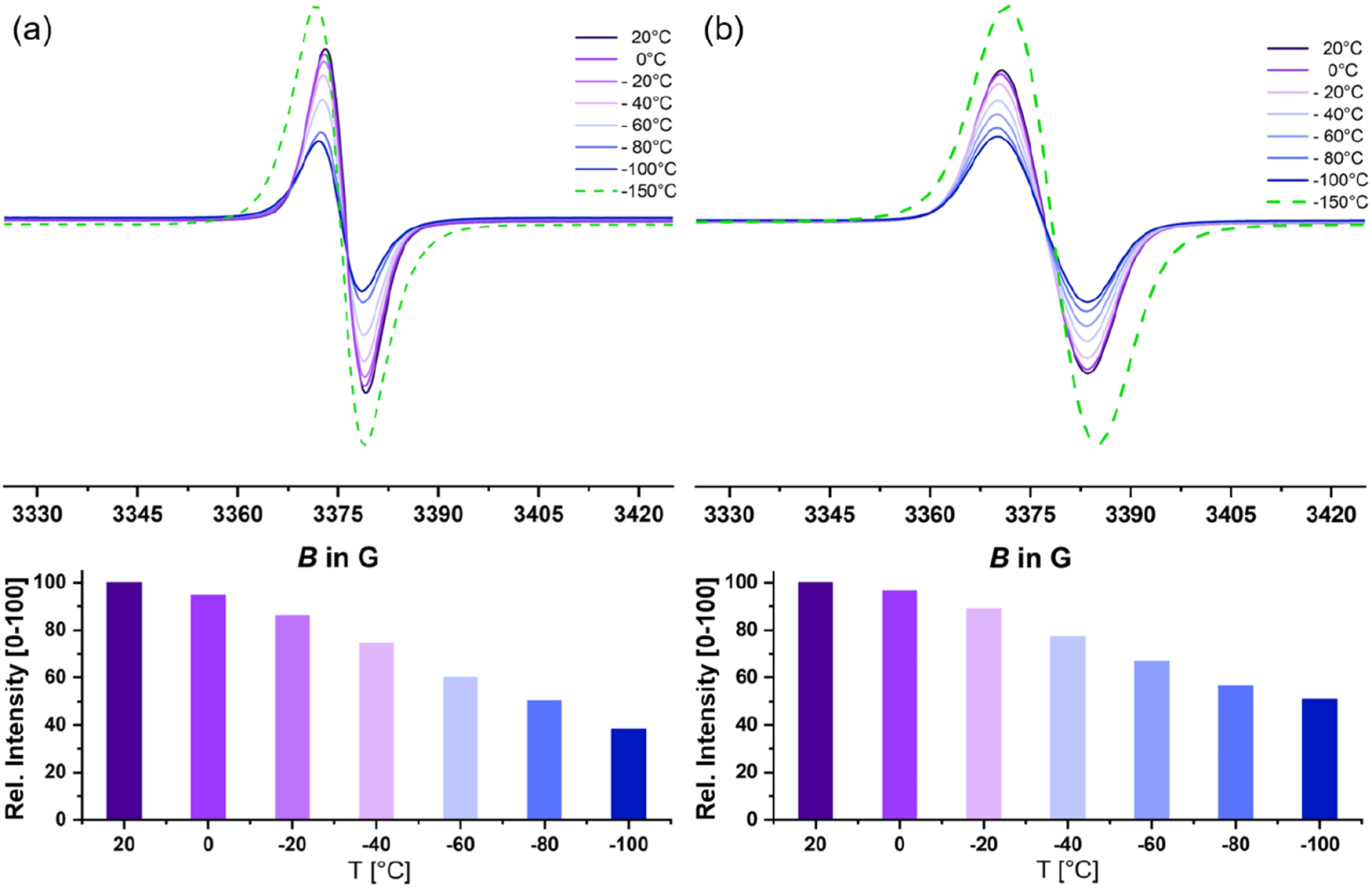
Temperature-dependent EPR spectra of (a) a 5 mM solution of cation **1** ^**+**^ and (b) a 5 mM solution of radical **1^•^** in CH_2_Cl_2_ as the solvent and in the glass at −150 °C (green dotted line). The bottom panel shows how the relative intensity of the solution spectra varies with *T*.

### Charge Transfer and Charge Resonance Excitations in 1^*n*+^ and 2^*n*+^ (*n* = 0, 1, 2) and Their Carbinol Precursors

Table 5 summarizes the experimentally observed electronic transitions of all relevant species of this study and compares them to TD-DFT computed data. In keeping with their dominant existence as tritylium-type cations, electronic absorption spectra of *in situ* generated **1** ^**+**^ and **2** ^**+**^ exhibit a characteristic, intense (ε_max_ ca. 48 · 10^3^ M^–1^·cm^–1^), vibrationally split band in the near-infrared (NIR) with distinct peaks at 880 and 995 nm for **1** ^**+**^, and at 899 and 1020 nm for **2** ^**+**^ (see Figure 10c,d). TD-DFT calculations reproduce these intense absorptions quite well, placing them at 788 or 784 nm, respectively. In both cases, the underlying excitation originates from the TAT-based HOMO–1, which is delocalized over the two remote indolyl-type subunits, and targets the tritylium-type LUMO, which is delocalized over all three phenyl rings that surround the methylium C atom, including the near TAT indolyl unit and the ethynyl spacer. It has hence distinct charge-transfer (CT) character and is readily identified as the so-called *y*-band of a triarylmethylium dye.^42,103^ The HOMO→LUMO transition, calculated at 1103 or 1003 nm, respectively, is of similar character, but has only weak intensity, thus likely being hidden under the low-energy tail of the former band. Figure 11 displays the frontier MOs that are involved in these transitions together with the corresponding electron density difference maps (EDDMs) that visualize the ensuing change of the electron density distribution (see also Figures S47 and S56 of the Supporting Information). The HOMO/HOMO–1→LUMO transition is fairly red-shifted compared to other tritylium dyes, including such which also engage in VT equilibria,^34,37,87,88,93,104,105^ but is similar to those in π-extended tris­(styryl)­methylium ions.^23,42,104,106–109^ Our quantum chemical studies also reproduced the additional, weaker electronic bands in the visible (vis) and the near UV of the electronic spectra at wavelengths close to the experimental data. According to these calculations, the vis band at lower energy (calcd. at ca. 490 nm; exp. value 522 nm (**1** ^**+**^) or 514 nm (**2** ^**+**^)) is also of TAT→CAr_3_^+^ CT character, while the band at higher energy involves CT from the acceptor-substituted aryl rings to the methylium C atom and the ethynyl-indolyl TAT unit. Excitations in the near UV arise mainly from π→π* transitions within the TAT chromophore.

**Table 5:** UV/vis/NIR Spectroscopic Data and TD-DFT Calculated Energies of the Electronic Transitions in **1-OH**, **2-OH**, **1^+^**, **2^+^**, **1^•^** and **2** and Their Dimers

neutral/reduced forms; λ in nm			cationic/oxidized forms; λ in nm		
**1-OH**	exp.	322, 365	**1-OH** ^**+^•^**^	exp.	328, 413, 740, 895, 1025, 1185, 2100
	calcd.	292, 313, 348		calcd.	297, 378, 408, 781, 1021, 3398
^**‘‘**^ **1** ^**^•^‘‘**^	exp.	319, 379, 444	**1** ^**+**^	exp.	309, 520, 880, 997
	calcd.	**1^•^**: 301, 346, 406, 470, 659, 666		calcd.	**1** ^**+**^: 281, 298, 492, 788, 1103
		**1**–**1**: 314, 316, 357			**1** ^**+••**^: 297, 340, 377, 451, 541, 683, 1036, 2870
			**1** ^**2+^•^**^	exp.	326, 396, 525, 751, 1011, 1175
				calcd.	297, 390, 456, 727, 1021, 1162
**2-OH**	exp.	323, 365, 424	**2-OH** ^**+^•^**^	exp.	327, 413, 744, 900, 1030, 1190, 1744
	calcd.	311, 348		calcd.	297, 378, 813, 1011, 3292
			**2-OH** ^**2+••**^	exp.	388, 460, 595, 756, 1050
				calcd.	328, 402, 677, 1055
^**‘‘**^ **2** ^**^•^‘‘**^	exp.	321, 369, 427	**2** ^**+**^	exp.	308, 417, 514, 898, 1023
	calcd.	**2^•^**: 305, 355, 406, 461, 596		calcd.	**2** ^**+**^: 281, 355, 404, 475, 784, 1003
		**2**–**2**: 317, 352			**2** ^**+••**^: 303, 334, 379, 453, 704, 1084, 3510

**Figure 10:**
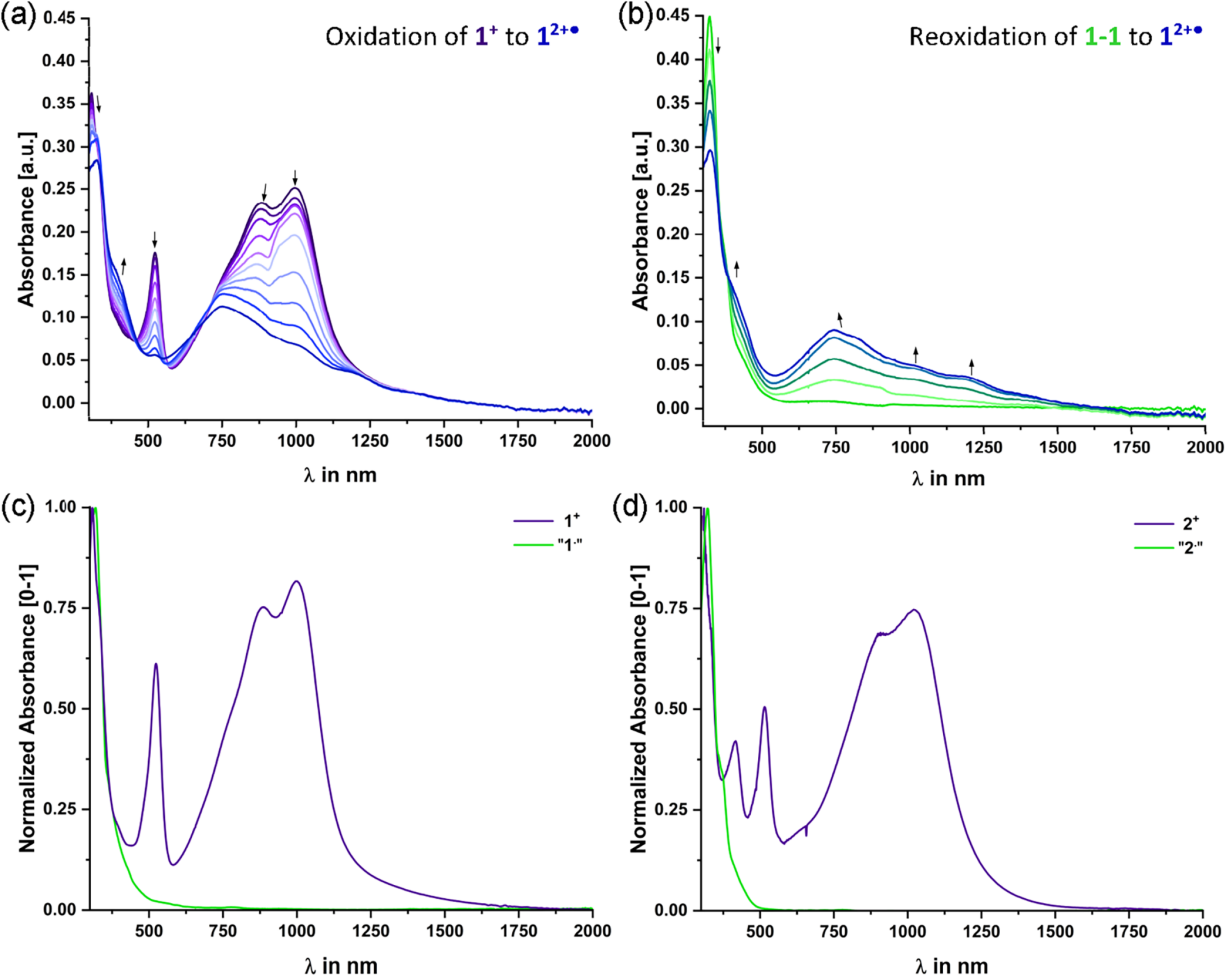
(a) Spectroscopic changes in the UV/vis/NIR range during electrolysis of **1** ^**+**^ to **1** ^**2+^•^**^ and (b) reoxidation of “**1** ^**^•^”**^ to **1** ^**2+^•^**^ in an OTTLE cell, and in C_2_H_4_Cl_2_/NBu_4_^+^ [BAr^F24^]^−^ (0.14 M) at r. t. (c) UV/vis/NIR spectrum of **1** ^**+**^ in CH_2_Cl_2_/NBu_4_^+^ [BAr^F24^]^−^ (0.04 M) and “**1^•^**’’ after addition of 1 equiv of Cp*_2_Fe to a solution of **1** ^**+**^ in toluene; both at r.t. (d) UV/vis/NIR spectrum of **2** ^**+**^ in CH_2_Cl_2_/NBu_4_^+^ [BAr^F24^]^−^ (0.04 M) and “**2**’’ after addition of 1 equiv of Cp*_2_Fe to a solution of **2** ^**+**^ in toluene; both at r.t.

**Figure 11:**
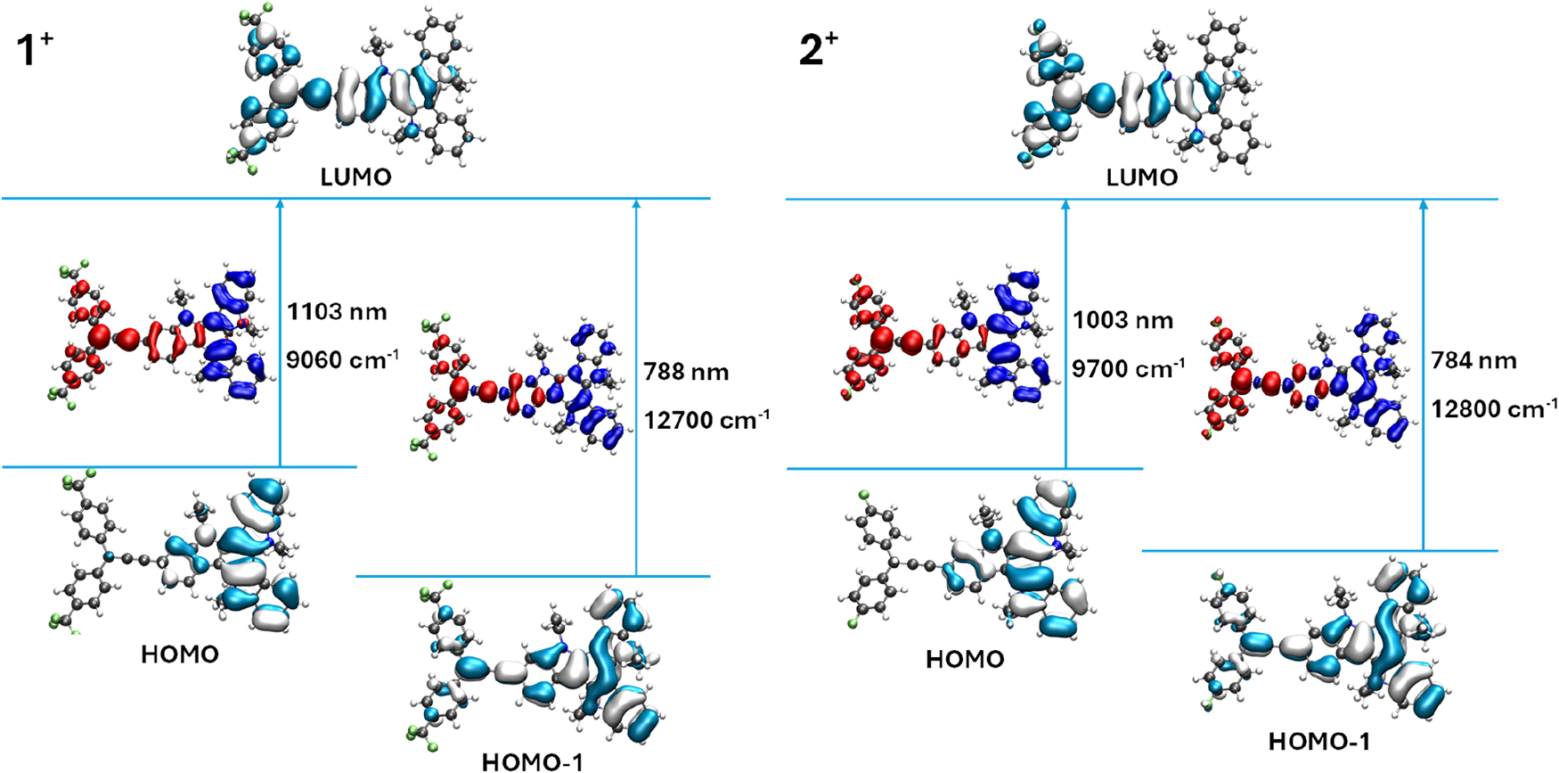
Contour diagrams of the frontier orbitals involved in the two lowest-lying CT-transitions of **1** ^**+**^ and **2** ^**+**^ and corresponding electron density difference maps (EDDMs).

We also monitored the changes in the UV/vis/NIR spectra concomitant with the oxidation or reduction of dyad **1** ^**+**^. For this purpose, we resorted to the *in situ* conditions of electrolysis of solutions of **1** ^**+**^ in CH_2_Cl_2_/NBu_4_^+^ [BAr^F24^]^−^ (0.14 M) inside a thin-layer electrolysis cell according to the design of Hartl et al.,^110^ with continuous spectroscopic monitoring. Figure 10a,b illustrate the results of spectroelectrochemical studies on **1** ^**+**^. Oxidation causes a bleaching of theTAT→tritylium CT transitions while the characteristic charge resonance (CR) bands of the TAT^+•^ chromophore^51,52,54^ with distinct maxima at 751, 1011, and 1175 nm develop (Figure 10a). Reduction of **1** ^**+**^ to the corresponding trityl radical likewise results in the complete loss of all tritylium-type absorptions in the vis and the NIR along with the growth of an intense UV band at λ = 319 nm and two shoulders at 375 and 450 nm (Figure 10c,d). Reoxidation of the neutral compounds with slowly increasing the applied potential directly proceeded to **1** ^**2+•**^ without the intermediacy of monomeric **1** ^**+**^, thereby signaling that dissociation of the dimers is slow, even after oxidation of their TAT constituents (Figure 10b). This also matches with the DFT-optimized structures of the neutral and dicationic forms of the dimers, which show that TAT oxidation does not affect the length of the C–C bond between the methyl C atoms (see Figures S42 and S43 of the Supporting Information).

The same absorption features as obtained after reduction of **1** ^**+**^ in the OTTLE cell were observed for chemically generated samples of the neutral compounds formed by reduction of **1** ^**+**^ and **2** ^**+**^ with decamethylferrocene in toluene. In this solvent, the as-formed decamethylferrocenium salt precipitates as a green solid so that it can be easily separated off. These spectra were devoid of any vis bands and resembled the calculated spectra of the neutral dimers **1–1** and **2–2** (see Figure 10c,10d; for TD-DFT computed spectra of monomeric radicals, see Figures S51 and S60 of the Supporting Information).

A CT-type excitation also accounts for the lowest-energy transition in the neutral carbinols **1-OH** and **2-OH**. Similar to the methylium cations, the donor MO distributes over the two remote indolyl rings, whereas the corresponding acceptor MO is largely constituted by the ethynyl-modified indolyl ring with additional contributions from the acceptor-substituted phenyl rings at the carbinol in the case of **1-OH**. In agreement with the strongly attenuated acceptor capabilities compared to the methylium cations, the underlying CT band is shifted to the near UV. The typical TAT based π→π* transitions are observed as the most intense band (ε ≈ 60 · 10^3^ M^–1^·cm^–1^) at even higher energy. Plots of the relevant orbitals as well as the electron density difference maps (EDDMs) of the individual transitions can be found as Figures S45 and S53 of the Supporting Information.

The chemically reversible TAT oxidations of the carbinol precursors at modestly positive half-wave potentials allowed us to also monitor the spectroscopic changes accompanying this process by means of UV/vis/NIR and IR spectroelectrochemistry (see Figures S34 to 37 of the Supporting Information).^110^ Just like in the cations, oxidation of the carbinols to their radical cations causes the growth of the characteristic CR excitations of an open-shell TAT^+•^ chromophore.^51,52,54^ Accompanying TD-DFT calculations on the radical cations suggest that the most intense of these transitions, peaking at 781 nm in **1-OH** ^**+•**^ and at 813 nm in **2-OH** ^**+•**^, is augmented with CT from the phenyl residues at the carbinol C atom. Graphical representations of the results of our TD-DFT calculations are provided in Figures S46 and S54 of the Supporting Information.

SEC experiments in the IR/NIR range reveal that oxidation causes the growth of an additional electronic band at even lower energies, at 2100 or 1720 nm (4700 or 5800 cm^–1^, see Figures S34 and S35 of the Supporting Information). This band is well accommodated by our TD-DFT calculations and is assigned to the β-HOSO→β-LUSO CR excitation within the TAT^+•^ chromophore. As TAT oxidation converts the original donor–acceptor dyad into an alkyne with two acceptor units, the moderately strong alkynyl C≡C stretching vibration initially observed at 2221 cm^–1^ almost vanishes. In contrast, the O–H stretching vibrations at 3552 cm^–1^ for **1-OH** and 3777 cm^–1^ for **2-OH** are conserved without any apparent band shift.

Further oxidation of **2-OH** to the corresponding dication causes the partial bleaching of the electronic band at the lowest energy while the vis band at 750 nm intensifies and a new band at 460 nm as well as a shoulder peak at 595 nm form. Our quantum chemical calculations assign these bands to CR excitations confined within the open-shell TAT^2+••^ chromophore, which still is a strongly coupled mixed-valent species, and a CT excitation where the diarylmethanol entity serves as the donor.

## Conclusions

The main finding of this study is that ethynyl-bridged triazatruxene-diarylmethylium dyads TAT-C≡C–C^+^(C_6_H_4_-4-R)_2_ with two *p*-trifluoromethylphenyl (**1** ^**+**^) or *p*-fluorophenyl (**2** ^**+**^) substituents at the methylium C atom equilibrate with their paramagnetic TAT^+^•^^-C≡C–C-(C_6_H_4_-4-R)_2_ valence tautomers (redox isomers) despite showing relatively large differences of the redox potentials for TAT-based oxidation and Tr^+^-centered reduction. Dimerization, presumably by C–C bond formation between their triarylmethyl constituents, seems to counterbalance the otherwise sizable energy difference of ca. 80 kJ/mol between the two valence tautomers. Direct evidence for the existence of dimers was obtained from cyclic voltammetry, where the reduction/oxidation waves of such species were already observed in solutions of the neat cations, i. e. prior to reduction to the neutral radicals. We also note that dimerization after reduction occurs at an appreciably faster rate than the dissociation of the dimers following oxidation, so that oxidized dimers **1** ^**+^•^**^ **–1** ^**+^•^**^ and **2** ^**+^•^**^ **–2** ^**+^•^**^ persist at least on the (spectro)­electrochemical time scale. Chemical reduction of the cations likewise causes extensive dimerization; nevertheless, small quantities of radical monomers are still detected by EPR spectroscopy.

Set against the background of the demonstrated multistate on-surface switching capabilities of TAT molecular units, whose states can be simultaneously switched and read out by STM techniques,^51,53,111,112^ the present dyads may offer the additional dimension of electronic bistability. Additional accessible states with differing magnetic properties make such compounds particularly attractive, if their overall switching behavior is retained upon surface deposition. Further work along these lines is aimed at bringing the TAT oxidation and tritylium reduction potentials closer together, e.g., by resorting to linkers other than ethynylene, and at stabilizing the diradical valence tautomers against dimerization.

## Supplementary Material



## Data Availability

The data underlying this study are available in the published article and its Supporting Information.

## Notes

Methods, materials and instrumentation, synthesis procedures, characterization data including ^1^H-, ^13^C­{^1^H}-NMR, 2D NMR and HR-ESI-MS spectra, cyclic and square wave voltammograms, results of UV/vis/NIR and IR spectroelectrochemical studies, EPR spectra, geometry-optimized, DFT-calculated structures of dimers along with the computed atomic coordinates, and TD-DFT calculations on **1-OH**, **2-OH**, **1^+^**, **2^+^**, **1–1** and **2–2** (PDF)

## References

[1] Heath J. R. (2009). Molecular
Electronics. Annu. Rev. Mater. Res..

[2] Heath J. R., Ratner M. A. (2003). Molecular Electronics. Phys.
Today.

[3] Ratner M. (2013). A brief history
of molecular electronics. Nat. Nanotechnol..

[4] Carroll R. L., Gorman C. B. (2002). The Genesis of Molecular Electronics. Angew. Chem., Int. Ed..

[5] Craig G. A., Roubeau O., Aromí G. (2014). Spin state
switching in 2,6-bis­(pyrazol-3-yl)­pyridine
(3-bpp) based Fe­(II) complexes. Coord. Chem.
Rev..

[6] Köbke A., Gutzeit F., Röhricht F., Schlimm A., Grunwald J., Tuczek F., Studniarek M., Longo D., Choueikani F., Otero E. (2020). Reversible coordination-induced spin-state switching
in complexes on metal surfaces. Nat. Nanotechnol..

[7] Gütlich P., Gaspar A. B., Garcia Y. (2013). Spin state switching in iron coordination
compounds. Beilstein J. Org. Chem..

[8] Ohmann R., Vitali L., Kern K. (2010). Actuated transitory
metal-ligand
bond as tunable electromechanical switch. Nano
Lett..

[9] Auwärter W., Seufert K., Bischoff F., Ecija D., Vijayaraghavan S., Joshi S., Klappenberger F., Samudrala N., Barth J. V. (2012). A surface-anchored molecular four-level conductance
switch based on single proton transfer. Nat.
Nanotechnol..

[10] Jaekel S., Richter A., Lindner R., Bechstein R., Nacci C., Hecht S., Kühnle A., Grill L. (2018). Reversible and Efficient Light-Induced Molecular Switching on an
Insulator Surface. ACS Nano.

[11] Iancu V., Hla S.-W. (2006). Realization of a
four-step molecular switch in scanning
tunneling microscope manipulation of single chlorophyll-a molecules. Proc. Natl. Acad. Sci..

[12] Tierney H. L., Murphy C. J., Jewell A. D., Baber A. E., Iski E. V., Khodaverdian H. Y., McGuire A. F., Klebanov N., Sykes E. C. H. (2011). Experimental
demonstration of a single-molecule electric motor. Nat. Nanotechnol..

[13] Mohn F., Repp J., Gross L., Meyer G., Dyer M. S., Persson M. (2010). Reversible bond formation
in a gold-atom-organic-molecule
complex as a molecular switch. Phys. Rev. Lett..

[14] Albrecht F., Fatayer S., Pozo I., Tavernelli I., Repp J., Peña D., Gross L. (2022). Selectivity in single-molecule
reactions by tip-induced redox chemistry. Science.

[15] Roubeau O., Colin A., Schmitt V., Clérac R. (2004). Thermoreversible
Gels as Magneto-Optical Switches. Angew. Chem..

[16] Bentley A. K., Ellis A. B., Lisensky G. C., Crone W. C. (2005). Suspensions of nickel
nanowires as magneto-optical switches. Nanotechnology.

[17] Huang X.-D., Xu Y., Fan K., Bao S.-S., Kurmoo M., Zheng L.-M. (2018). Reversible
SC-SC Transformation involving 4 + 4 Cycloaddition of Anthracene:
A Single-Ion to Single-Molecule Magnet and Yellow-Green to Blue-White
Emission. Angew. Chem., Int. Ed..

[18] Liu X., Xie J., Niklas J., Turner E. E., Yuan D., Anderson J. S., Rack J. J., Poluektov O. G., Yu L. (2022). Donor–Acceptor
Conjugated Copolymers Containing Transition-Metal Complex: Intrachain
Magnetic Exchange Interactions and Magneto-Optical Activity. Chem. Mater..

[19] Feringa B. L., Jager W. F., de Lange B. (1993). Organic materials
for reversible
optical data storage. Tetrahedron.

[20] Ratera I., Sporer C., Ruiz-Molina D., Ventosa N., Baggerman J., Brouwer A. M., Rovira C., Veciana J. (2007). Solvent tuning from
normal to inverted marcus region of intramolecular electron transfer
in ferrocene-based organic radicals. J. Am.
Chem. Soc..

[21] Ratera I., Ruiz-Molina D., Vidal-Gancedo J., Novoa J. J., Wurst K., Letard J.-F., Rovira C., Veciana J. (2004). Supramolecular photomagnetic
materials: photoinduced dimerization of ferrocene-based polychlorotriphenylmethyl
radicals. Chem. - Eur. J..

[22] Ratera I., Ruiz-Molina D., Renz F., Ensling J., Wurst K., Rovira C., Gütlich P., Veciana J. (2003). A new valence tautomerism
example in an electroactive ferrocene substituted triphenylmethyl
radical. J. Am. Chem. Soc..

[23] Fajarí L., Papoular R., Reig M., Brillas E., Jorda J. L., Vallcorba O., Rius J., Velasco D., Juliá L. (2014). Charge transfer
states in stable neutral and oxidized radical adducts from carbazole
derivatives. J. Org. Chem..

[24] D’Avino G., Grisanti L., Guasch J., Ratera I., Veciana J., Painelli A. (2008). Bistability in Fc-PTM
crystals: the role of intermolecular
electrostatic interactions. J. Am. Chem. Soc..

[25] D’Avino G., Grisanti L., Painelli A., Guasch J., Ratera I., Veciana J. (2009). Cooperativity
from electrostatic interactions: understanding
bistability in molecular crystals. CrystEngComm.

[26] Guasch J., Grisanti L., Souto M., Lloveras V., Vidal-Gancedo J., Ratera I., Painelli A., Rovira C., Veciana J. (2013). Intra- and
intermolecular charge transfer in aggregates of tetrathiafulvalene-triphenylmethyl
radical derivatives in solution. J. Am. Chem.
Soc..

[27] Souto M., Morales D. C., Guasch J., Ratera I., Rovira C., Painelli A., Veciana J. (2014). Intramolecular electron transfer
and charge delocalization in bistable donor–acceptor systems
based on perchlorotriphenylmethyl radicals linked to ferrocene and
tetrathiafulvalene units. J. Phys. Org. Chem..

[28] Calbo J., Aragó J., Otón F., Lloveras V., Mas-Torrent M., Vidal-Gancedo J., Veciana J., Rovira C., Ortí E. (2013). Tetrathiafulvalene-based
mixed-valence acceptor-donor-acceptor triads: a joint theoretical
and experimental approach. Chem. - Eur. J..

[29] Guasch J., Grisanti L., Jung S., Morales D., D’Avino G., Souto M., Fontrodona X., Painelli A., Renz F., Ratera I., Veciana J. (2013). Bistability
of Fc-PTM-Based Dyads:
The Role of the Donor Strength. Chem. Mater..

[30] Mayorga
Burrezo P., Franco C., Caballero R., Mas-Torrent M., Langa F., López Navarrete J. T., Rovira C., Veciana J., Casado J. (2018). Oligothienylenevinylene
Polarons and Bipolarons Confined between Electron-Accepting Perchlorotriphenylmethyl
Radicals. Chem. - Eur. J..

[31] Gilabert A., Fajarí L., Sirés I., Reig M., Brillas E., Velasco D., Anglada J. M., Juliá L. (2017). Twisted intramolecular
charge transfer in a carbazole-based chromophore: the stable [(4-N-carbazolyl)-2,3,5,6-tetrachlorophenyl]­bis­(2,3,5,6-tetrachlorophenyl)­methyl
radical. New J. Chem..

[32] Witt A., Heinemann F. W., Khusniyarov M. M. (2015). Bidirectional photoswitching of magnetic
properties at room temperature: ligand-driven light-induced valence
tautomerism. Chem. Sci..

[33] Witt A., Heinemann F. W., Sproules S., Khusniyarov M. M. (2014). Modulation
of magnetic properties at room temperature: coordination-induced valence
tautomerism in a cobalt dioxolene complex. Chem.
- Eur. J..

[34] Rehse A., Linseis M., Azarkh M., Drescher M., Winter R. F. (2023). Valence
Tautomerism in Chromium Half-Sandwich Triarylmethylium Dyads. Inorganics.

[35] Casper L. A., Linseis M., Demeshko S., Azarkh M., Drescher M., Winter R. F. (2021). Tailoring Valence Tautomerism by
Using Redox Potentials:
Studies on Ferrocene-Based Triarylmethylium Dyes with Electron-Poor
Fluorenylium and Thioxanthylium Acceptors. Chem.—Eur.
J..

[36] Casper L. A., Deuter K. L., Rehse A., Winter R. F. (2024). Dimerization of
9-Phenyl-ferroceno2,3indenylmethyl Radicals: Electrochemical and Spectroelectrochemical
Studies. ACS Org. Inorg. Au.

[37] Casper L. A., Oßwald S., Anders P., Rosenbaum L.-C., Winter R. F. (2020). Extremely Electron-Poor
Bis­(diarylmethylium)-Substituted
Ferrocenes and the First Peroxoferrocenophane. Z. Anorg. Allg. Chem..

[38] Nau M., Casper L. A., Haug G., Linseis M., Demeshko S., Winter R. F. (2023). Linker permethylation
as a means to foster valence
tautomerism and thwart dimerization in ferrocenyl-triarylmethylium
cations. Dalton Trans..

[39] Neumann W. P., Uzick W., Zarkadis A. K. (1986). Sterically
hindered free radicals.
14. Substituent-dependent stabilization of para-substituted triphenylmethyl
radicals. J. Am. Chem. Soc..

[40] Olah G. A. (1970). Stable
carbonium ions in solution. Science.

[41] Hinz A., Labbow R., Reiß F., Schulz A., Sievert K., Villinger A. (2015). Synthesis and structure of tritylium salts. Struct. Chem..

[42] Duxbury D. F. (1993). The photochemistry
and photophysics of triphenylmethane dyes in solid and liquid media. Chem. Rev..

[43] Dünnebacke D., Neumann W. P., Penenory A., Stewen U. (1989). Über sterisch
gehinderte freie Radikale, XIX. Stabile 4,4′,4″-trisubstituierte
Triphenylmethyl-Radikale. Chem. Ber..

[44] van
der Hart W. J. (1970). The E.S.R. spectra of triarylmethyl radicals. Mol. Phys..

[45] Fleck N., Heubach C. A., Hett T., Haege F. R., Bawol P. P., Baltruschat H., Schiemann O. (2020). SLIM: A Short-Linked,
Highly Redox-Stable
Trityl Label for High-Sensitivity In-Cell EPR Distance Measurements. Angew. Chem. Int. Ed..

[46] Song H., Pietrasiak E., Lee E. (2022). Persistent Radicals Derived from
N-Heterocyclic Carbenes for Material Applications. Acc. Chem. Res..

[47] Bobko A. A., Dhimitruka I., Zweier J. L., Khramtsov V. V. (2007). Trityl
radicals as persistent dual function pH and oxygen probes for in vivo
electron paramagnetic resonance spectroscopy and imaging: concept
and experiment. J. Am. Chem. Soc..

[48] García-Frutos E. M., Gómez-Lor B. (2008). Synthesis and self-association properties of functionalized *C*
_3_-symmetric hexakis­(p-substituted-phenylethynyl)­triindoles. J. Am. Chem. Soc..

[49] Li N., Chen Y., Duan S., Chen G., Xu Y., Tong H., Sanehira Y., Miyasaka T., Li A., Wang X.-F. (2020). Planar perovskite
solar cells using triazatruxene-based
hyperbranched conjugated polymers and small molecule as hole-transporting
materials. J. Photochem. Photobiol. A: Chem..

[50] Yuan M.-S., Li T.-B., Wang W.-J., Du Z.-T., Wang J.-R., Fang Q. (2012). Thiophene-functionalized
octupolar triindoles: synthesis and photophysical
properties. Spectrochim. Acta, Part A.

[51] Vogelsang L., Birk T., Paschke F., Bauer A., Enenkel V., Holz L. M., Fonin M., Winter R. F. (2023). Ferrocenyl-Substituted
Triazatruxenes: Synthesis, Electronic Properties, and the Impact of
Ferrocenyl Residues on Directional On-Surface Switching on Ag(111). Inorg. Chem..

[52] Ruiz C., García-Frutos E. M., da Silva Filho D. A., López Navarrete J. T., Ruiz
Delgado M. C., Gómez-Lor B. (2014). Symmetry Lowering in Triindoles:
Impact on the Electronic
and Photophysical Properties. J. Phys. Chem.
C.

[53] Vogelsang L., Birk T., Kostrzewa F., Bauch N., Maier G., Rendler J., Linseis M., Fonin M., Winter R. F. (2025). Synthesis,
electronic properties and on-surface switching behaviour of triazatruxene
dimers and tetramers. J. Mater. Chem. C.

[54] Thomas T.
G., Chandra Shekar S., Swathi R. S., Gopidas K. R. (2017). Triazatruxene radical
cation: a trigonal class III mixed valence system. RSC Adv..

[55] Robin M. B., Day P. (1968). Mixed Valence Chemistry-A Survey and Classification. Adv. Inorg. Chem. Radiochem..

[56] Wang Y., Chen S., Zhang G. (2023). An alternative
approach to triazatruxene
synthesis and derivatization to a boron difluoride complex. Org. Chem. Front..

[57] Lai W.-Y., He Q.-Y., Chen D.-Y., Huang W. (2008). Synthesis and Characterization
of Starburst 9-Phenylcarbazole/Triazatruxene Hybrids. Chem. Lett..

[58] Li X.-C., Wang C.-Y., Lai W.-Y., Huang W. (2016). Triazatruxene-based
materials for organic electronics and optoelectronics. J. Mater. Chem. C.

[59] Lai W., Liu D., Huang W. (2010). Triazatruxene-containing
hyperbranched polymers: Microwave-assisted
synthesis and optoelectronic properties. Sci.
Chin. Lett..

[60] Qian X., Zhu Y.-Z., Song J., Gao X.-P., Zheng J.-Y. (2013). New donor-π-acceptor
type triazatruxene derivatives for highly efficient dye-sensitized
solar cells. Org. Lett..

[61] Li Q., Zhang Y., Xie Z., Zhen Y., Hu W., Dong H. (2022). Polycyclic aromatic
hydrocarbon-based organic semiconductors: ring-closing
synthesis and optoelectronic properties. J.
Mater. Chem. C.

[62] Feng X., Wu J., Ai M., Pisula W., Zhi L., Rabe J. P., Müllen K. (2007). Triangle-Shaped
Polycyclic Aromatic Hydrocarbons. Angew. Chem.
Int. Ed..

[63] Liu L., Yang G., Duan Y., Geng Y., Wu Y., Su Z. (2014). The relationship between intermolecular interactions and charge transport
properties of trifluoromethylated polycyclic aromatic hydrocarbons. Org. Electron..

[64] Aumaitre C., Morin J.-F. (2019). Polycyclic Aromatic
Hydrocarbons as Potential Building
Blocks for Organic Solar Cells. Chem. Rec..

[65] Feng X., Pisula W., Müllen K. (2009). Large polycyclic aromatic hydrocarbons:
Synthesis and discotic organization. Pure Appl.
Chem..

[66] Kilaru S., Gade R., bhongiri Y., Tripathi A., Chetti P., Pola S. (2022). Organic materials based on hetero
polycyclic aromatic hydrocarbons
for organic thin-film transistor applications. Mater. Sci. Semicond. Process..

[67] Bhattacharyya K., Mukhopadhyay T. K., Datta A. (2016). Controlling electronic
effects and
intermolecular packing in contorted polyaromatic hydrocarbons (c-PAHs):
towards high mobility field effect transistors. Phys. Chem. Chem. Phys..

[68] Mori T., Takeuchi H., Fujikawa H. (2005). Field-effect
transistors based on
a polycyclic aromatic hydrocarbon core as a two-dimensional conductor. J. Appl. Phys..

[69] Zhang L., Cao Y., Colella N. S., Liang Y., Brédas J.-L., Houk K. N., Briseno A. L. (2015). Unconventional,
chemically stable,
and soluble two-dimensional angular polycyclic aromatic hydrocarbons:
from molecular design to device applications. Acc. Chem. Res..

[70] Hesse H. C., Schaffer C., Hundschell C., Narita A., Feng X., Müllen K., Nickel B., Schmidt-Mende L. (2012). Large polycyclic
aromatic hydrocarbons for application in donor–acceptor photovoltaics. Phys. Satus Solidi A.

[71] Zhang D., Duan L. (2019). Polycyclic Aromatic
Hydrocarbon Derivatives toward Ideal Electron-Transporting
Materials for Organic Light-Emitting Diodes. J. Phys. Chem. Lett..

[72] Bin J.-K., Hong J.-I. (2011). Efficient blue organic
light-emitting diode using anthracene-derived
emitters based on polycyclic aromatic hydrocarbons. Org. Electron..

[73] Wagner J., Zimmermann Crocomo P., Kochman M. A., Kubas A., Data P., Lindner M. (2022). Modular Nitrogen-Doped
Concave Polycyclic Aromatic
Hydrocarbons for High-Performance Organic Light-Emitting Diodes with
Tunable Emission Mechanisms. Angew. Chem., Int.
Ed..

[74] Prins R., Reinders F. J. (1969). Electron spin resonance of the cation of ferrocene. J. Am. Chem. Soc..

[75] Prins R. (1970). Electronic
structure of the ferricenium cation. Mol. Phys..

[76] van
Order N., Geiger W. E., Bitterwolf T. E., Rheingold A. L. (1987). Mixed-valent cations of dinuclear chromium arene complexes:
electrochemical, spectroscopic, and structural considerations. J. Am. Chem. Soc..

[77] Castellani M. P., Connelly N. G., Pike R. D., Rieger A. L., Rieger P. H. (1997). EPR Spectra
of [Cr­(CO)_2_L­(η-C_6_Me_6_)]^+^ (L = PEt_3_, PPh_3_, P­(OEt)_3_, P­(OPh)_3_): Analysis of Line Widths and Determination
of Ground State Configuration from Interpretation of ^31^P Couplings. Organometallics.

[78] Camire
Ohrenberg N., Paradee L. M., DeWitte R. J., Chong D., Geiger W. E. (2010). Spectra and Synthetic-Time-Scale Substitution Reactions
of Electrochemically Produced [Cr­(CO)_3_(η^6^-arene)]^+^ Complexes. Organometallics.

[79] Prins R., Korswagen A. R. (1970). Substituent
effects in the ESR spectra of ferricenium
cations. J. Org. Chem..

[80] Ishizu K., Mukai K., Shibayama A., Kondo K. (1977). ENDOR Studies on Low-Symmetry
Triphenylmethyl with ortho- or para -Methoxy Substituents. Bull. Chem. Soc. Jpn..

[81] Neumann W. P., Penenory A., Stewen U., Lehnig M. (1989). Sterically hindered
free radicals. 18. Stabilization of free radicals by substituents
as studied by using triphenylmethyls. J. Am.
Chem. Soc..

[82] Souto M., Guasch J., Lloveras V., Mayorga P., López
Navarrete J. T., Casado J., Ratera I., Rovira C., Painelli A., Veciana J. (2013). Thermomagnetic Molecular System Based
on TTF-PTM Radical: Switching the Spin and Charge Delocalization. J. Phys. Chem. Lett..

[83] Jacobson P. (1905). Zur ≪Triphenylmethyl≫-Frage. Ber. Dtsch. Chem. Ges..

[84] Ishiyama T., Hartwig J. (2000). A Heck-Type Reaction
Involving Carbon–Heteroatom
Double Bonds. Rhodium­(I)-Catalyzed Coupling of Aryl Halides with N-Pyrazyl
Aldimines. J. Am. Chem. Soc..

[85] Lucas P., Mehdi N. E., Ho H. A., Bélanger D., Breau L. (2000). Expedient Synthesis of Symmetric
Aryl Ketones and of Ambient-Temperature
Molten Salts of Imidazole. Synthesis.

[86] Brookhart M., Grant B., Volpe A. F. (1992). (3,5-(CF_3_)_2_C_6_H_3_)_4_B]^−^[H­(OEt_2_)_2_]^+^: a convenient reagent for generation
and stabilization of cationic, highly electrophilic organometallic
complexes. Organometallics.

[87] Casper L. A., Wursthorn L., Geppert M., Roser P., Linseis M., Drescher M., Winter R. F. (2020). 4-Ferrocenylphenyl-Substituted Tritylium
Dyes with Open and Interlinked C^+^Ar_2_ Entities:
Redox Behavior, Electrochromism, and a Quantitative Study of the Dimerization
of Their Neutral Radicals. Organometallics.

[88] Casper L. A., Ebel V., Linseis M., Winter R. F. (2021). Five shades of green:
substituent influence on the (spectro-) electrochemical properties
of diferrocenyl­(phenyl)­methylium dyes. Dalton
Trans..

[89] Sheppard W. A. (1963). The Effect
of Fluorine Substitution on the Electronic Properties of Alkoxy, Alkylthio
and Alkylsulfonyl Groups. J. Am. Chem. Soc..

[90] Siodła T., Ozimiński W. P., Hoffmann M., Koroniak H., Krygowski T. M. (2014). Toward
a physical interpretation of substituent effects: the case of fluorine
and trifluoromethyl groups. J. Org. Chem..

[91] Heuer A. M., Coste S. C., Singh G., Mercado B. Q., Mayer J. M. (2023). A Guide
to Tris­(4-Substituted)-triphenylmethyl Radicals. J. Org. Chem..

[92] Strohbusch F. (1972). Polarographische
Untersuchungen der Substituenteneffekte in Triarylmethylkationen. Ber. Bunsenges. Phys. Chem..

[93] Oßwald S., Breimaier S., Linseis M., Winter R. F. (2017). Polyelectrochromic
Vinyl Ruthenium-Modified Tritylium Dyes. Organometallics.

[94] Oßwald S., Casper L. A., Anders P., Schiebel E., Demeshko S., Winter R. F. (2018). Electrochemical, Spectroelectrochemical, Mößbauer,
and EPR Spectroscopic Studies on Ferrocenyl-Substituted Tritylium
Dyes. Chem. J. Eur..

[95] Reig M., Gozálvez C., Jankauskas V., Gaidelis V., Grazulevicius J. V., Fajarí L., Juliá L., Velasco D. (2016). Stable All-Organic
Radicals with Ambipolar Charge Transport. Chem.
- Eur. J..

[96] Castellanos S., Velasco D., López-Calahorra F., Brillas E., Julia L. (2008). Taking advantage of the radical character of tris­(2,4,6-trichlorophenyl)­methyl
to synthesize new paramagnetic glassy molecular materials. J. Org. Chem..

[97] Richardson D. E., Taube H. (1981). Determination of E20-E10
in multistep charge transfer by stationary-electrode
pulse and cyclic voltammetry: application to binuclear ruthenium ammines. Inorg. Chem..

[98] The MathWorks, Inc . MATLAB Release R2025a; The MathWorks, Inc., 2025.

[99] Sinclair J., Kivelson D. (1968). Electron spin resonance
studies of substituted triphenylmethyl
radicals. J. Am. Chem. Soc..

[100] Maier, M. Triazatruxenes with peripheral redox sites: Improved synthetical strategies and control of chemical and physical properties; PhD thesis, Univesity of Konstanz 2019.

[101] Dalal D. P., Eaton S. S., Eaton G. R. (1981). The effects of lossy
solvents on quantitative EPR studies. J. Magn.
Reson. 1969.

[102] Lewis I. C., Singer L. S. (1965). Electron Spin Resonance of Radical
Cations Produced by the Oxidation of Aromatic Hydrocarbons with SbCl5. J. Chem. Phys..

[103] Lewis G. N., Calvin M. (1939). The Color of Organic
Substances. Chem. Rev..

[104] Hellwinkel D., Fritsch H. (1989). Phenylvinylog erweiterte
Triphenylmethylium-Systeme. Chem. Ber..

[105] Ballesteros P., Cuadrado A., Gilabert A., Fajarí L., Sirés I., Brillas E., Almajano M. P., Velasco D., Anglada J. M., Juliá L. (2019). Formation of a stable biradical triplet
state cation versus a closed shell singlet state cation by oxidation
of adducts of 3,6-dimethoxycarbazole and polychlorotriphenylmethyl
radicals. Phys. Chem. Chem. Phys..

[106] Aaron C., Barker C. C. (1971). Steric effects in di- and tri-arylmethane
dyes. Part X. Electronic absorption spectra of bridged derivatives
of malachite green and crystal violet. J. Chem.
Soc. B.

[107] Lewis L. M., Indig G. L. (2000). Solvent effects
on the spectroscopic
properties of triarylmethane dyes. Dyes Pigm..

[108] Sengupta S., Kumar Sadhukhan S. (2000). Tris­[4-(ferrocenylvinyl)-3-methylphenyl]­methylium
tetrafluoroborate: a strongly absorbing organometallic near-IR dye. J. Mater. Chem..

[109] Sengupta S., Sadhukhan S. K. (2000). Trivinylogs
of Crystal Violet: Synthesis
and absorption properties of new near-IR dyes. J. Chem. Soc., Perkin Trans. 1.

[110] Krejčik M., Daněk M., Hartl F. (1991). Simple construction
of an infrared optically transparent thin-layer electrochemical cell. J. Electroanal. Interfac. Electrochem..

[111] Bauer A., Maier M., Schosser W. M., Diegel J., Paschke F., Dedkov Y., Pauly F., Winter R. F., Fonin M. (2020). Tip-Induced Inversion of the Chirality
of a Molecule’s Adsorption
Potential Probed by the Switching Directionality. Adv. Mater..

[112] Bauer A., Birk T., Paschke F., Fuhrberg A., Diegel J., Becherer A.-K., Vogelsang L., Maier M., Schosser W. M., Pauly F. (2024). Fully
Reprogrammable 2D Array of Multi-State Molecular Switching Units. Adv. Mater..

