# Supplementary material for: Redox Isomerism in Ethynyl-Bridged Triazatruxene-Diarylmethylium Dyads
Source: J Org Chem. 2025 Nov 4;90(50):17716–28. doi: 10.1021/acs.joc.5c01973 (PMC12723676; doi:10.1021/acs.joc.5c01973)
Supplement: Supplementary file 1 [file jo5c01973_si_001.pdf]

Redox Isomerism in Ethynyl-Bridged Triazatruxene-Diarylmethylium Dyads

Lars Vogelsang*, Felix Kuschel*, Anja Rehse*, Michael Linseis* and Rainer F. Winter*,[a]

*Fachbereich Chemie, Universität Konstanz, Universitätsstraße 10, 78464 Konstanz, Germany

[a]rainer.winter@uni-konstanz.de

List of contents

Materials and Methods 2

NMR spectroscopy 2

Mass spectrometry 2

Electrochemical investigations 2

UV/vis/NIR Spectroscopy and Spectroelectrochemistry 2

Quantum chemistry 3

EPR spectrocopy and spin-counting 3

Experimental Section 4

NMR and Mass Spectra 7

Voltammetric measurements 17

UV/vis/NIR and IR Spectroelectrochemistry 25

EPR spectroscopy 27

Quantum Chemistry 28

References 111

Materials and Methods

NMR spectroscopy

NMR spectra were measured on a *Bruker* Avance III *400* (^{1}H-NMR: 400 MHz, ^{13}C-NMR: 101 MHz), or on a *Jeol* JNM-ECZR (^{1}H-NMR: 500 MHz, ^{13}C-NMR: 121 MHz) spectrometer. For all spectra, deuterated solvents were used. Referencing was done based on the resonance signals of the residual protonated solvent for ^{1}H-NMR spectra or on the ^{13}C resonance signal for ^{13}C-NMR spectra.

Mass spectrometry

High-resolution ESI mass spectra on the carbinol precursors **1-OH** and **2-OH** were acquired on CH_2Cl_2 solutions of the respective compounds with a *LTC Orbitrap Velos* by *Thermo Fischer Scientific* in positive ion mode. The minimum resolution was 60000 FWHM. Calibration was performed with a Pierce LTQ Velos ESI Positive Solution by *Thermo Fischer Scientific*. Simulation was done based on the natural abundances of the respective elements using Xcalibur™ by *Thermo Fischer Scientific.*

Electrochemical investigations

Cyclic voltammograms were measured under argon atmosphere in the CH_2Cl_2/NBu_4^+ $[BAr^{F24}]^-$ ($[BAr^{F24}]^-$ = $[B\{C_6H_3(CF_3)_2\text{-}3,5\}_4]^-$) 0.04 M electrolyte. $Na^+[BAr^{F24}]^-$ was prepared using 3,5-bis(trifluoromethyl)bromobenzene according to the literature.[1] Subsequent cation-exchange for NBu_4^+ was also conducted according to literature-known procedures.[2] Voltammetric measurements were performed with a computer-controlled *BASi* potentiostat. A custom-made cylindrical, vacuum-tight, single-compartment cell with a platinum or a glassy carbon disk electrode from *BASi* embedded in a PCTFE plastic body as working electrode (surface area: Pt = 2.56 mm^2, glassy carbon = 7.1 mm^2) was used for measurement. A coiled platinum wire was used as counter electrode, while a coiled silver wire served as (pseudo)reference electrode. These electrodes were sealed into glass capillaries and fixated to sidearms of the cell using Quickfit screws. The working electrode was polished with diamond pastes of 1 µm and 0.25 µm grain size prior to measurements. The working electrode was inserted into the top port of the cell through a Quickfit adapter. Referencing was performed using either decamethylferrocene (Cp^*_2Fe, $E_{1/2}$ = –550 mV vs $FcH^{0/+}$) or cobaltocenium hexafluorophosphate ($[Cp_2Co]^+[PF_6]^-$, $E_{1/2}$ = –1330 mV vs $FcH^{0/+}$) as internal calibrants. The calibrant was added after all scans of interest were acquired, and representative sets of additional scans were performed in the presence of the redox standard. Potentials are provided using the polarographic plotting convention relative to the ferrocene/ferrocenium (FcH/FcH^+) scale ($E_{1/2}$ (FcH/FcH^+) = 0 mV).

UV/vis/NIR Spectroscopy and Spectroelectrochemistry

FT-IR/NIR spectra were recorded using a *Bruker* Tensor III setup with detection over the range of 1000 to 11500 cm^{-1}. UV/vis/NIR spectra were measured using a *TIDAS* fiber optic diode array spectrometer using MCS UV/Vis and PGS NIR instruments from *J&M*. Extinction coefficients were determined by using solutions in Hellma quartz cuvettes with 0.1 cm thickness. Spectroelectrochemical measurements were conducted in an OTTLE (optically transparent thin-layer electrochemical) cell according to the design of Hartl et al.[3] The cell is custom-built with CaF_2 windows, Pt-mesh working and counter electrodes, and a Ag/AgCl pseudo-reference electrode. The measurements were conducted in dry and degassed solutions of NBu_4^+ $[BAr^{F24}]^-$ (0.14 M) in 1,2-$Cl_2C_2H_4$. Electrochemical responses in this solvent (CAUTION; 1,2-$Cl_2C_2H_4$ is a suspected carcinogen) are very similar to those in CH_2Cl_2, while the higher boiling point of 1,2-$Cl_2C_2H_4$ helps to avoid the formation of gas bubbles, which can result in unwanted mixing or even the loss of electrical contact at the electrodes. Potentials were applied using a *Wenking Pos 2* potenstiostat by *Intelligent Controls GmbH*.

Quantum chemistry

Quantum chemical calculations were performed using GAUSSIAN 16 program packages.[4] Alkyne functionalities were modeled with dummy atoms to avoid angles of 180° during optimization. Electronic transitions were rendered by the time-dependent density functional theory (TD-DFT) approach. Geometrical optimization and calculation of the molecular orbitals was performed using triple-ζ basis sets (6-31G(d))[5] for all atoms. The PBE1PBE functional was used for all compounds.[6] Solvent effects were considered using the polarizable conductor continuum model (CPCM).[7] Molecular orbitals are depicted in blue and white for positive and negative signs of the wavefunctions. Electron density difference maps (EDDMs) for analysing electronic transitions are provided in blue (electron density loss) and red (electron density gain). Isovalues were set to 0.02 for all molecular orbitals (MOs) and to 0.001 for spin orbitals.

EPR spectrocopy and spin-counting

EPR spectra for spin counting were measured on an X-band spectrometer MiniScope MS5000 by *Magnettech GmbH*, controlled by ESR Studio 1.63.0. *T*-dependent EPR studies were conducted with a HO3 temperature controller equipped with an external thermostat with liquid nitrogen as cooling agent. All cations and radicals were freshly prepared in the glovebox as solutions in CH_2Cl_2 and measured in sealed EPR-tubes. The following parameters were uniformly used during the experiments.

Sweep time:	60 s
Modulation	0.6 mT
Power:	6.3096 mW

Quantitative spin counting employed double integration of the EPR resonances of samples of known concentrations using a calibration line recorded for solutions of the stable standard $DPPH^{\bullet}$ ($DPPH^{\bullet}$ = diphenylpicrylhydrazyl) at different concentrations and at r. t. according to a procedure described in the literature.[8] Calculations were done using the following equations:

$$\text{Amount of radical [\%]} = \frac{\textit{Absolute integral of the EPR signal of compound } \boldsymbol{N} \textit{ at r. t.}}{\textit{Reference value of a DPPH}^{\bullet} \textit{ solution} \cdot [\boldsymbol{N}]} \cdot 100\ \%$$

$$\mathrm{VT}_{exp}\ [\%] = \frac{\textit{Absolute integral of the EPR signal of } \boldsymbol{N}^{+} \textit{ at r. t.}}{\textit{Reference value of a DPPH}^{\bullet} \textit{ solution } [\mathrm{M}] \cdot [\boldsymbol{N}^{+}]} \cdot 100\ \%$$

$$\mathrm{VT}_{corr}\ [\%] = \left(\mathrm{VT}_{exp} - \frac{\mathrm{VT}_{exp}}{100 - \frac{100 - \text{Amount of radical}}{100}} \right) \cdot 2$$

For resolving hyperfine structuring of the trityl radicals and picturing the spectra of the tritylium ions, we recorded EPR spectra on 1.7 mM (**1**$^{+}$, **1**$^{\bullet}$) or 1.0 mM (**2**$^{+}$, **2**$^{\bullet}$) samples of the radicals in CH_2Cl_2 on a tabletop X-band spectrometer MiniScope 400 with a temperature controller model H03 by *Magnettech*.

Experimental Section

All syntheses were carried out under nitrogen atmosphere using common Schlenk techniques, if necessary. Solvents were dried over suitable drying agents, deoxygenated by purging with dinitrogen, and stored under inert atmosphere. All starting materials were either purchased from commercial suppliers or prepared according to the indicated published procedures: 4,4'-bis(trifluoromethyl)benzophenone,[9] 4,4'-difluoro-benzophenone,[9] **2-A$_1$-^{Et}TAT**[10] and Brookhart's acid.[11]

2-(4,4'-Di(trifluoromethyl)-diphenylmethylalcohol-ethynyl)-*N,N',N''*-triethyltriazatruxene **(1-OH)**

92 mg (0.21 mmol, 1.0 equiv.) of **2-A$_1$-^{Et}TAT** were dissolved in 10 mL of dry THF and cooled to –78 °C. Subsequently, 0.13 mL (0.32 mmol, 1.5 equiv.) of a 2.5 M solution of ^{n}BuLi in hexane were added dropwise. The reaction mixture was stirred for 90 minutes at this temperature. Subsequently, 109 mg of 4,4'-bis(trifluoromethyl)benzophenone (0.34 mmol, 1.6 equiv.) were added dropwise in 5 mL of dry THF. The reaction was allowed to reach r. t. and to stir overnight. The reaction mixture was poured into 200 mL of H_2O. The phases were extracted with CH_2Cl_2 (3×50 mL). The combined organic phases were dried over Na_2SO_4 and the solvents were removed under reduced pressure. The crude product was extracted into mixture (6:1/ vol:vol) of pentane / diethyl ether (3×40 mL) and the solvents were removed under reduced pressure. The remaining solid was purified *via* column chromatography using CH_2Cl_2/ petroleum ether as eluent (1:1/ vol:vol). The product was isolated as microcrystalline, orange powder in a yield of 26 % (0.05 mmol, 55 mg).

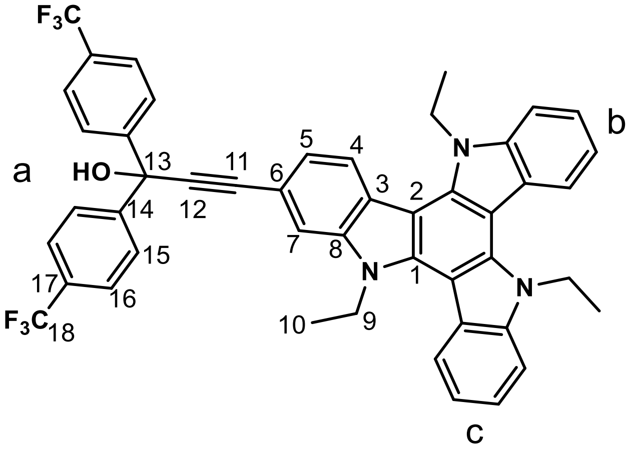

^{1}H-NMR (400 MHz, CD_2Cl_2, 300 K) δ[ppm] = 8.34 (vt, J = 8.4 Hz, 2H, H-4$_{b,c}$), 8.28 (d, J = 8.4 Hz, 1H, H-4$_a$), 7.90 (d, J = 7.7 Hz, 2H, H-16$_a$), 7.78 (d, J = 1.5 Hz, 1H, H-7$_a$), 7.74 – 7.58 (m, 6H, H-15$_a$ und H-7$_{b,c}$), 7.55 – 7.44 (m, 5H, H-5$_a$ + H-6$_{b,c}$), 7.37 (ddd, J = 8.2, 7.1, 1.2 Hz, 2H, H-5$_{c,b}$), 5.09 – 4.94 (m, 6H, H-9$_{a-c}$), 3.12 (s, 1H, -O*H*), 1.68 – 1.58 (m, 9H, H-10$_{a-c}$).

^{13}C{H}-NMR (101 MHz, CD_2Cl_2, 300 K) δ[ppm] = 148.6 (d, $^5J_{C-F}$ = 1.4 Hz, C-14$_a$), 140.9 (s, C-8$_{b,c}$), 140.4 (s, C-8$_a$), 139.7 (s, C-1$_{a,b,c}$), 139.3 (s, C-1$_{a-c}$), 138.5 (s, C-1$_{a-c}$), 130.4 (q, $^2J_{CF}$ = 32.5 Hz, C-17$_a$), 125.7 (q, $^3J_{CF}$ = 3.7 Hz, C-16$_a$), 124.5 (s, C-6$_a$), 124.2 (q, $^1J_{CF}$ = 272.2 Hz, C-18$_a$), 123.7 (s, C-5$_a$), 123.6 (s, C-2$_{b,c}$), 123.5 (s C-6$_{b\ or\ c}$), 123.3 (s, C-6$_{b\ or\ c}$), 121.7 (s, C4$_{b/c}$), 121.6 (s, 4$_{b/c}$), 121.4 (s, C-4$_a$), 120.1 (s, C-5$_{b\ or\ c}$), 120.1 (s, C-5$_{b\ or\ c}$), 115.0 (s, C-2$_a$), 113.6 (s, C-7$_a$), 110.6 (s, C-7$_{b\ or\ c}$), 110.6 (s, C-7$_{b\ or\ c}$), 103.8 (s, C-3$_a$), 103.3 (s, C-3$_{b,c}$), 90.2 (s, C-11$_a$), 89.4 (s, C-12$_a$), 74.5 (s, C-13$_a$), 41.9 (s, C-9$_{a-c}$), 15.7 (s, C-10$_a$), 15.6 (s, C-10$_{b\ or\ c}$), 15.5 (s, C-10$_{b\ or\ c}$).

^{19}F-NMR (376 MHz, CD_2Cl_2, 300 K) δ[ppm] = –62.56 (s, C**F$_3$**).

MS (ESI) m/z: [**1**+H]$^+$ Calcd. for 755.27; Found 755.27, [**1-OH**]$^+$ Calcd. 771.27; Found 771.27 (height: 0.4), [**1-OH**+H]$^+$ Calcd. 772.27; Found 772.27 (height: 1).

2-(4,4'-Difluoro-diphenylmethylalcohol-ethynyl)-*N,N',N''*-triethyltriazatruxene (**2-OH**)

92 mg (0.21 mmol, 1.0 equiv.) of **2-A$_1$-^{Et}TAT** were dissolved in 10 mL of dry THF and cooled to –78 °C. Subsequently, 0.13 mL (0.32 mmol, 1.5 equiv.) of a 2.5 M solution of ^{n}BuLi in hexane were added dropwise. The reaction mixture was stirred for 90 minutes. Subsequently, 69 mg of 4,4'-difluoro-benzophenone (0.34 mmol, 1.6 equiv.) were added dropwise in 5 mL of dry THF. The reaction was allowed to reach r. t. and afterwards stirred overnight. The reaction mixture was poured into 200 mL of H_2O. The phases were extracted with CH_2Cl_2 (3×50 mL). The combined organic phases were dried over Na_2SO_4 and the solvents were removed under reduced pressure. The crude product was extracted with a pentane/ diethyl ether solution (3:1/ vol:vol) three times and the solvents were removed under reduced pressure. The solid was purified *via* column chromatography using CH_2Cl_2/ petroleum ether as eluent (1:1/ vol:vol). The product was isolated as crystalline, orange powder in a yield of 42 % (0.09 mmol, 50 mg).

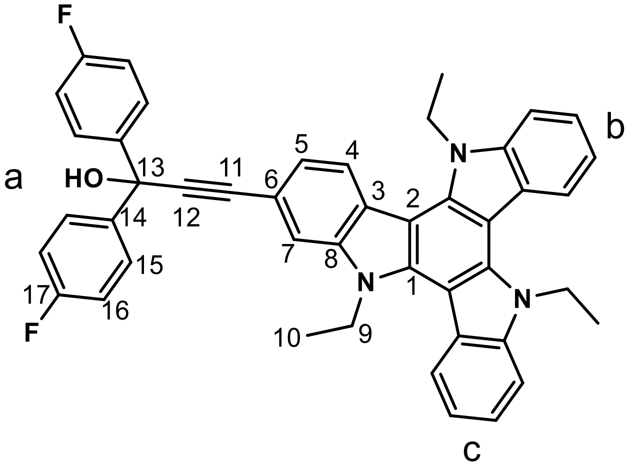

^{1}H-NMR (400 MHz, CD_2Cl_2, 300 K) δ[ppm] = 8.34 (vt, *J* = 7.8 Hz, 2H, H-4$_{b,c}$), 8.27 (d, *J* = 8.4 Hz, 1H, H-4$_a$), 7.77 (d, *J* = 1.4 Hz, 1H, H-7$_a$), 7.75 – 7.70 (m, 4H, H-15$_a$), 7.67 (dt, *J* = 8.3, 1.4 Hz, 2H, H-7$_{b,c}$), 7.53 – 7.42 (m, 3H, H-5$_{a\text{-}c}$), 7.37 (ddd, *J* = 8.1, 7.0, 1.1 Hz, 2H, H-6$_{b,c}$), 7.14 – 7.03 (m, 4H, H-16$_a$), 5.12 – 4.90 (m, 6H, H-9$_{a\text{-}c}$), 2.98 (s, 1H, -O*H*), 1.65 – 1.59 (m, 9H, H-10$_{a\text{-}c}$).

^{13}C{H}-NMR (101 MHz, CD_2Cl_2, 300 K) δ[ppm] = 162.9 (d, $^1J_{CF}$ = 246.1 Hz, C-17$_a$), 141.9 (d, $^4J_{CF}$ = 3.0 Hz, C-14$_a$), 141.4 (s, C-8$_{c/b}$), 140.8 (s, C-8$_a$), 140.0, 139.6, 138.9 (s, C-1$_{a/b/c}$), 128.6 (d, $^3J_{CF}$ = 8.2 Hz, C-15$_a$), 124.5 (s, C-6$_a$), 124.0 (s, C-2$_{b,c}$), 123.80, 123.79, (C-5$_{b,c}$), 123.7 (s, C-5$_a$) 122.10, 122.08 (C-4$_{b,c}$), 121.9 (s, C-4$_a$), 120.6 (C-6$_{b,c}$), 116.2 (s, C-2$_a$), 115.6 (d, $^2J_{CF}$ = 22.0 Hz, C-16$_a$), 114.0 (s, C-7$_a$), 111.0, 110.99 (C-7$_{b,c}$), 104.1 (s, C-3$_a$), 103.6 (s, C-3$_{b \text{ or } c}$), 103.6 (s, C-3$_{b \text{ or } c}$) 91.0 (s, C-11$_a$), 88.5 (s, C-12$_a$), 74.6 (s, C-13$_a$), 42.3 (s, C-9$_{a\text{-}c}$), 15.9 (s, C-10$_a$), 15.8 (s, C-10$_{a \text{ or } b}$), 15.8 (s, C-10$_{a \text{ or } b}$).

^{19}F-NMR (376 MHz, CD_2Cl_2, 300 K) δ[ppm] = –105.76 (s, **F**–Aryl).

MS (ESI) m/z: [**2**]$^+$ Calcd. for 654.27; Found 654.27, [**2-OH**+H]$^+$ Calcd. 671.27; Found 671.27, [**2-2**+H]$^+$ Calcd. 1309.55; Found 1309.55.

Cations 1⁺ and 2⁺ were generated freshly in situ from the respective carbinol by adding one equivalent of Brookhart´s acid,[11] $[H(OEt_2)]^+$ $[BAr^{F24}]^-$, in CH_2Cl_2 or the 0.04 M CH_2Cl_2/ NBu_4^+ $[BAr^{F24}]^-$ electrolyte, which resulted in an immediate color change from pale orange to intense pink or red-brown. Successful conversion was verified by recording UV/vis spectra prior to conducting voltammetric studies or recording EPR spectra. Cyclic and square wave voltammetry confirmed that the initial TAT oxidation of the respective carbinol **1-OH** or **2-OH** was no longer detected. Extinction coefficients are based on those of the carbinol precursors and accounting for concentration changes due to sample dilution. For referencing of the voltammograms, $[Cp_2Co]^+$ $[PF_6]^-$ had to be used as the internal standard.

For EPR studies, the cations for these studies were made from balanced amounts of **1-OH** and **2-OH** that were dissolved in a defined volume of CH_2Cl_2. To these solutions, 1 equiv. of Brookhart's acid in a defined volume of CH_2Cl_2 was added. For spin-counting, the quantity of the carbinol was chosen so as to yield a 5 mM solution of the corresponding cations. The one-electron reduced forms were generated by addition of an excess (>1 equiv.) of solid decamethylferrocene, Cp^*_2Fe, to solutions of the cationic compounds. For improved resolution of the EPR spectra, the solutions of the neutral compounds were diluted to a final concentration of 1.7 mM (**1•**) or 1.0 mM (**2•**). The EPR tubes were filled and sealed inside a glovebox.

NMR and Mass Spectra

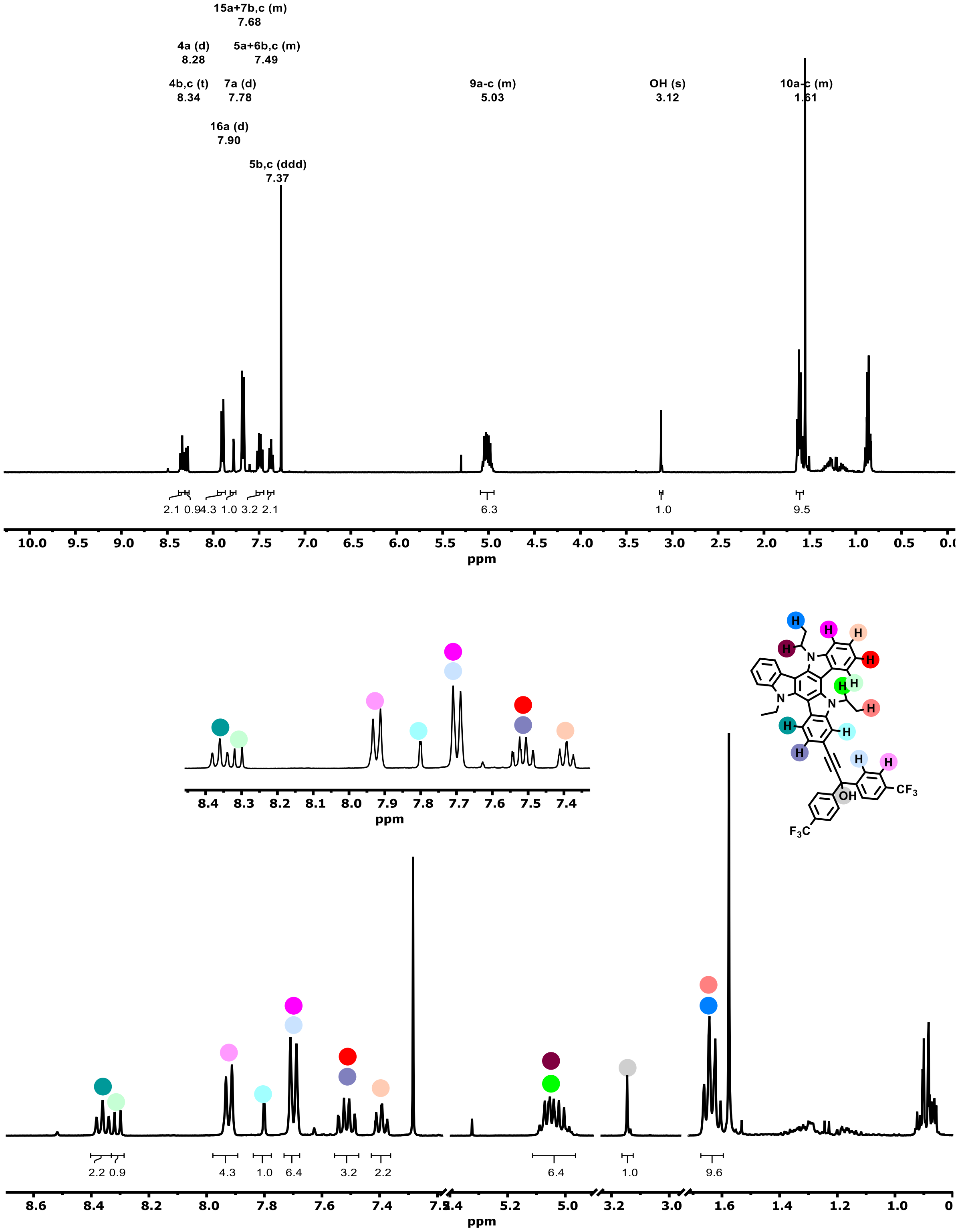

Figure S1. ^{1}H-NMR spectrum of **1-OH** in $CDCl_3$ (400 MHz, 300 K).

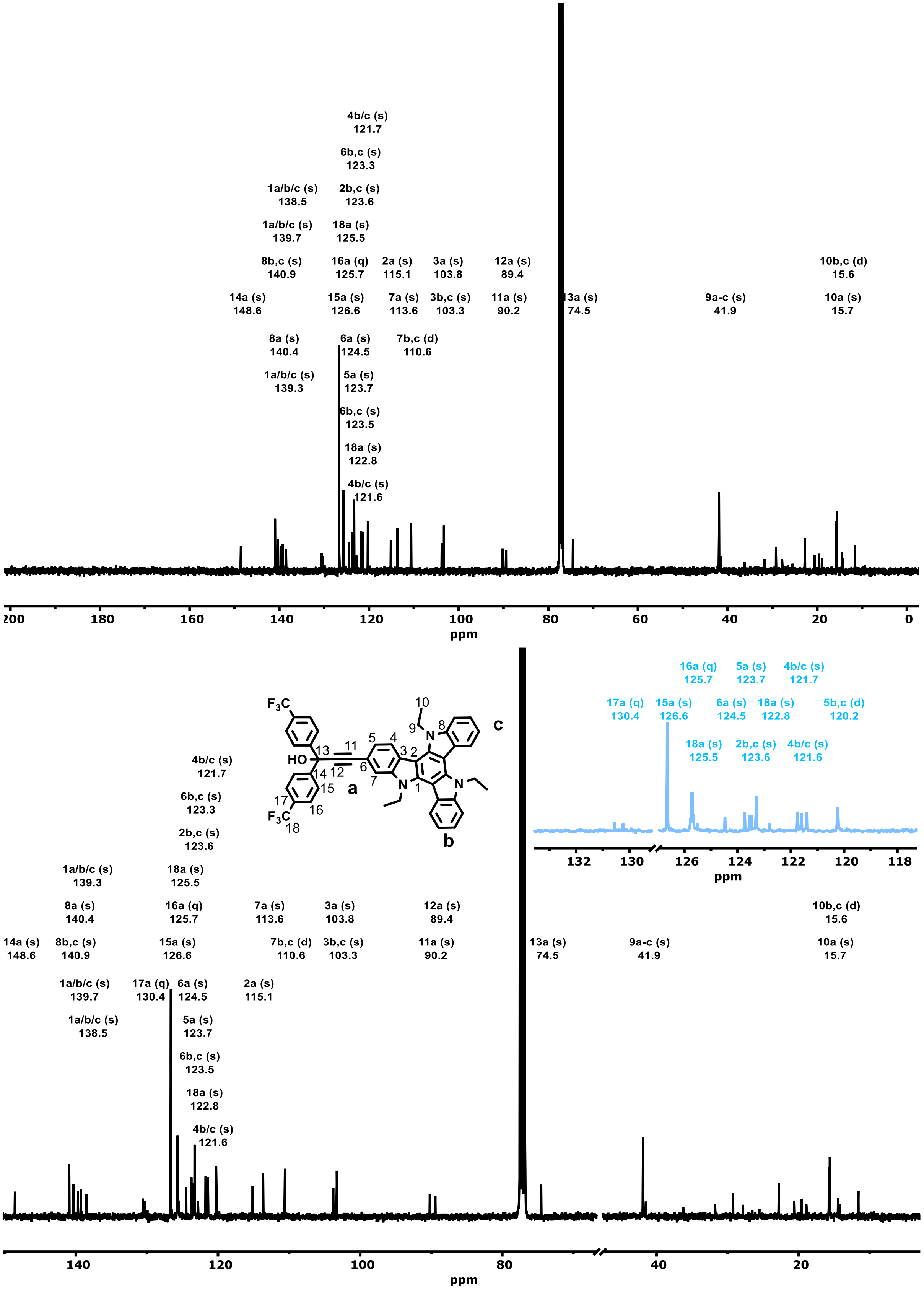

Figure S2. $^{13}C\{^{1}H\}$-NMR spectrum of **1-OH** in $CDCl_3$ (101 MHz, 300 K).

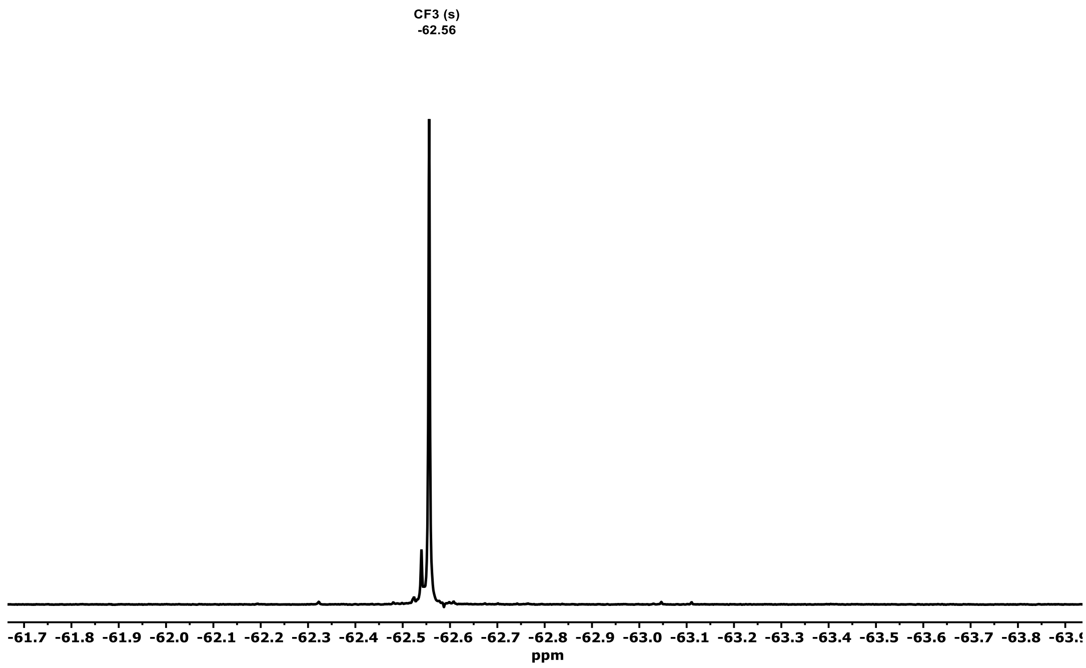

Figure S3. ^{19}F-NMR spectrum of **1-OH** in $CDCl_3$ (376 MHz, 300 K).

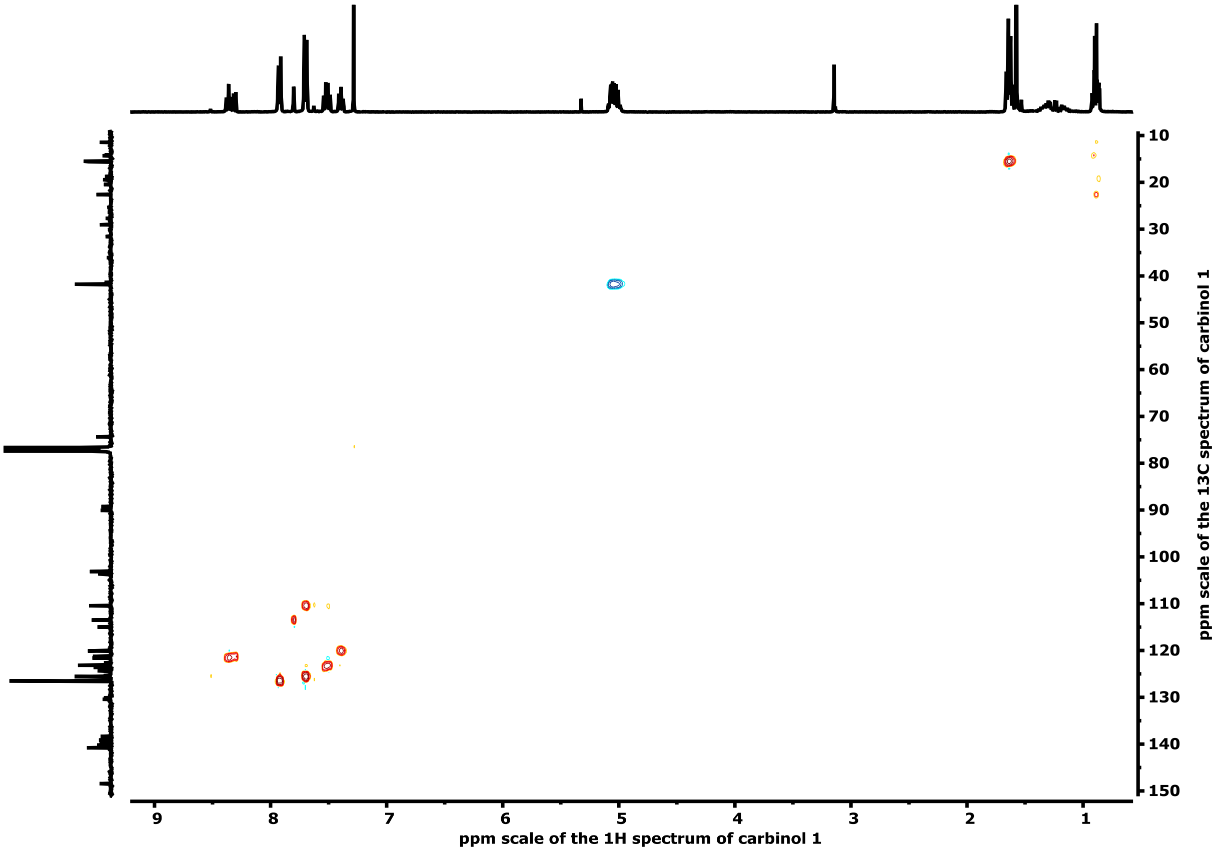

Figure S4. 2D HSQC spectrum of **1-OH** in $CDCl_3$ (^1H: 400 MHz, $^{13}C\{^1H\}$: 101 MHz, 300 K).

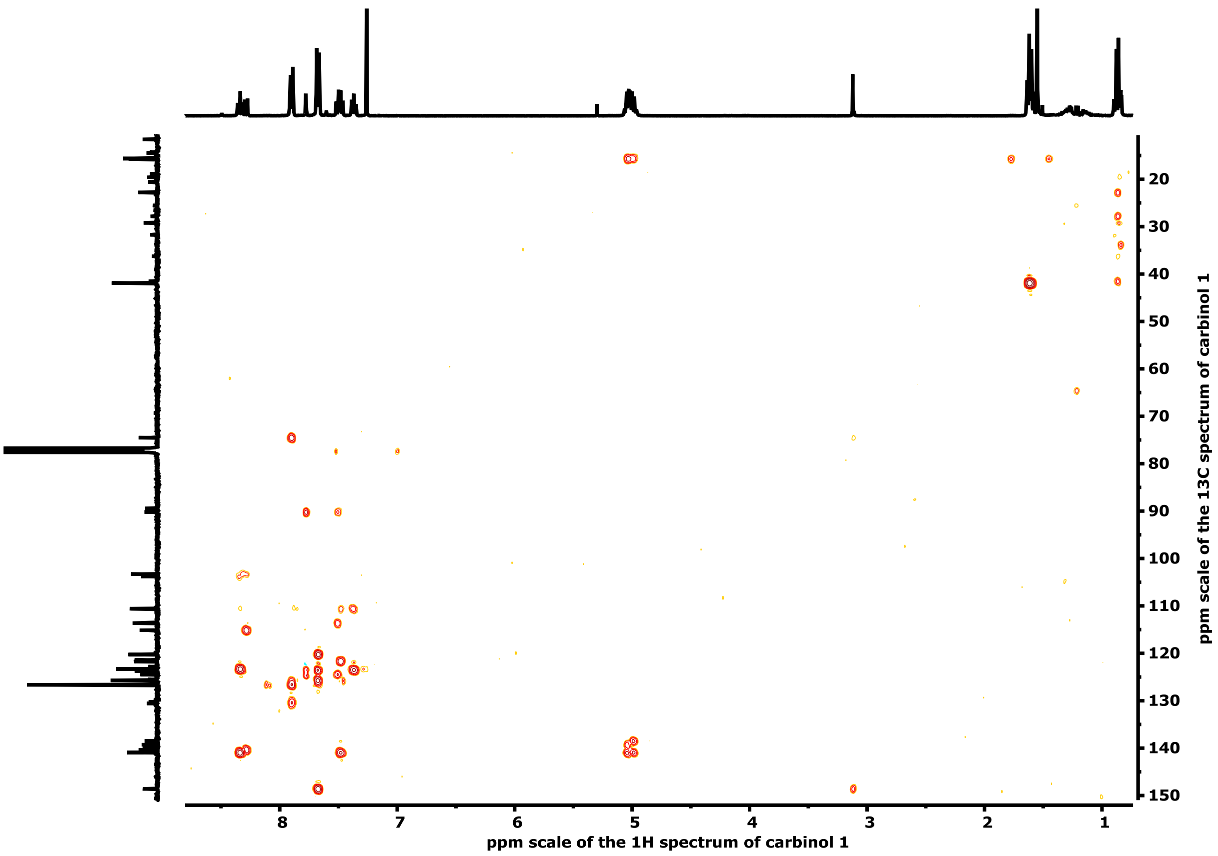

Figure S5. 2D HMBC spectrum of **1-OH** in $CDCl_3$ (^1H: 400 MHz, $^{13}C\{^1H\}$: 101 MHz, 300 K).

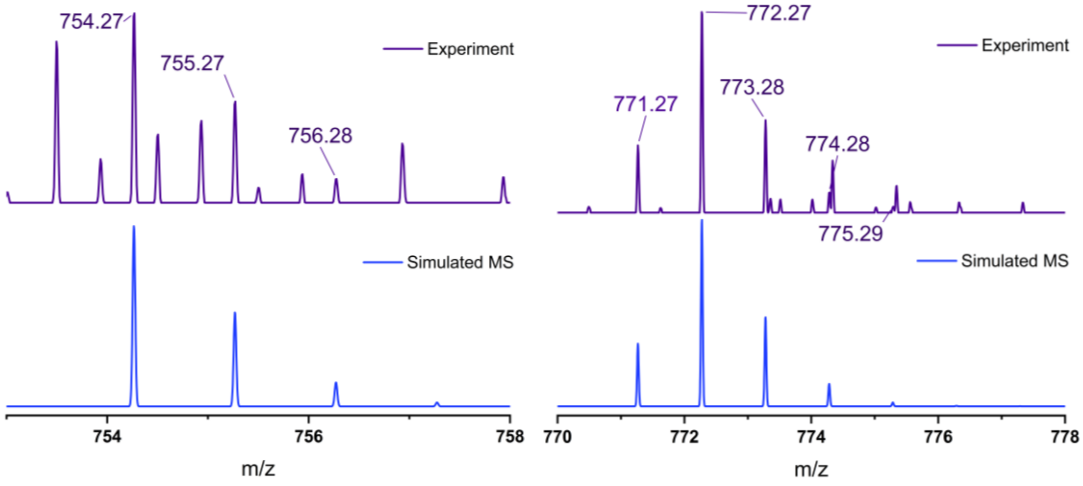

Figure S6. Molecular ion peaks in the ESI mass spectrum (CH_2Cl_2) of **1-OH** with mass peaks of the ions {**1**-OH}$^+$ (left) **1**$^+$ and {**1**+H}$^+$ (both right). The bottom panel shows the calculated mass peaks of the different isotopomers as simulated ion peaks.

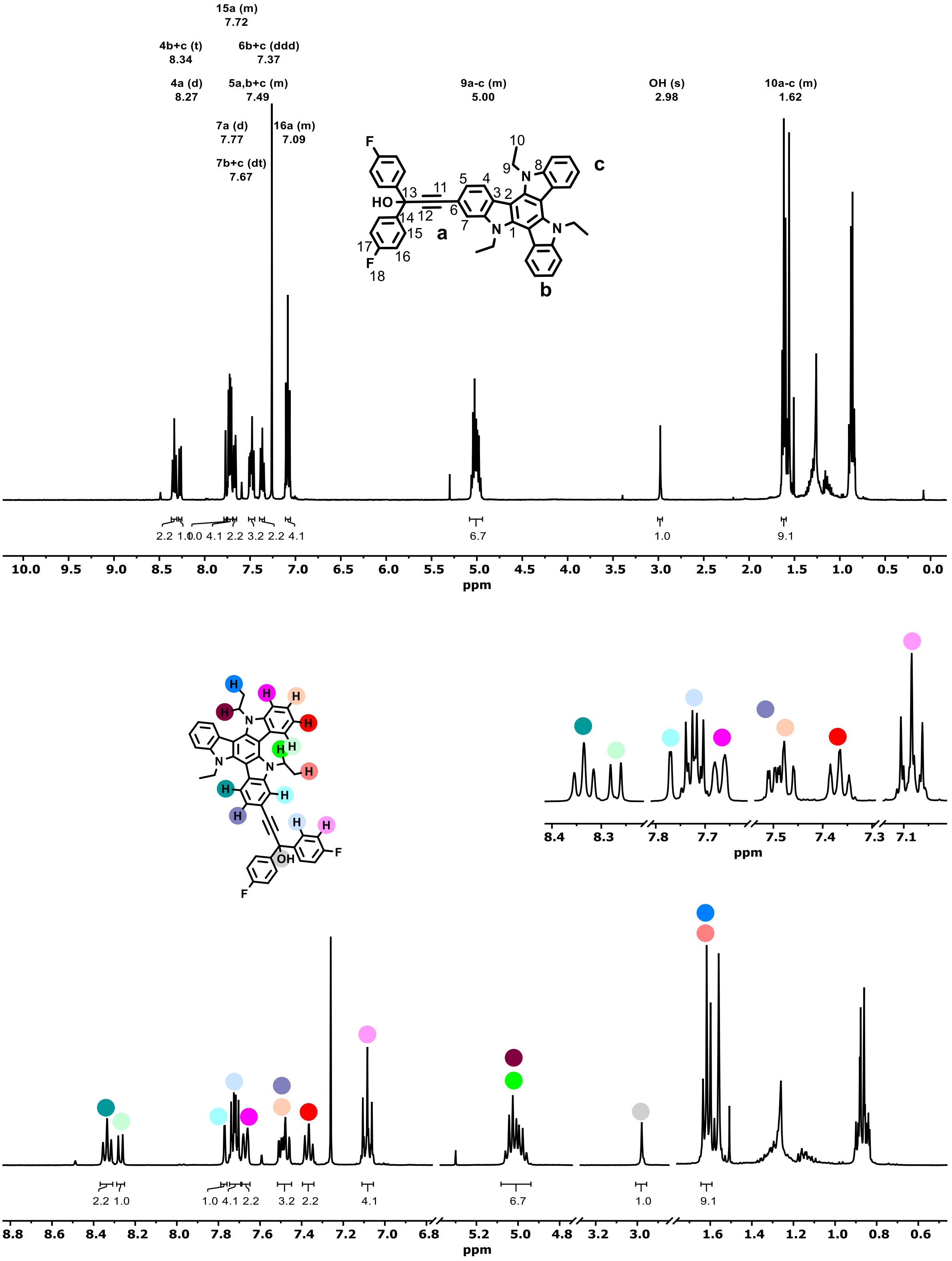

Figure S7. ^{1}H-NMR spectrum of **2-OH** in $CDCl_3$ (400 MHz, 300 K).

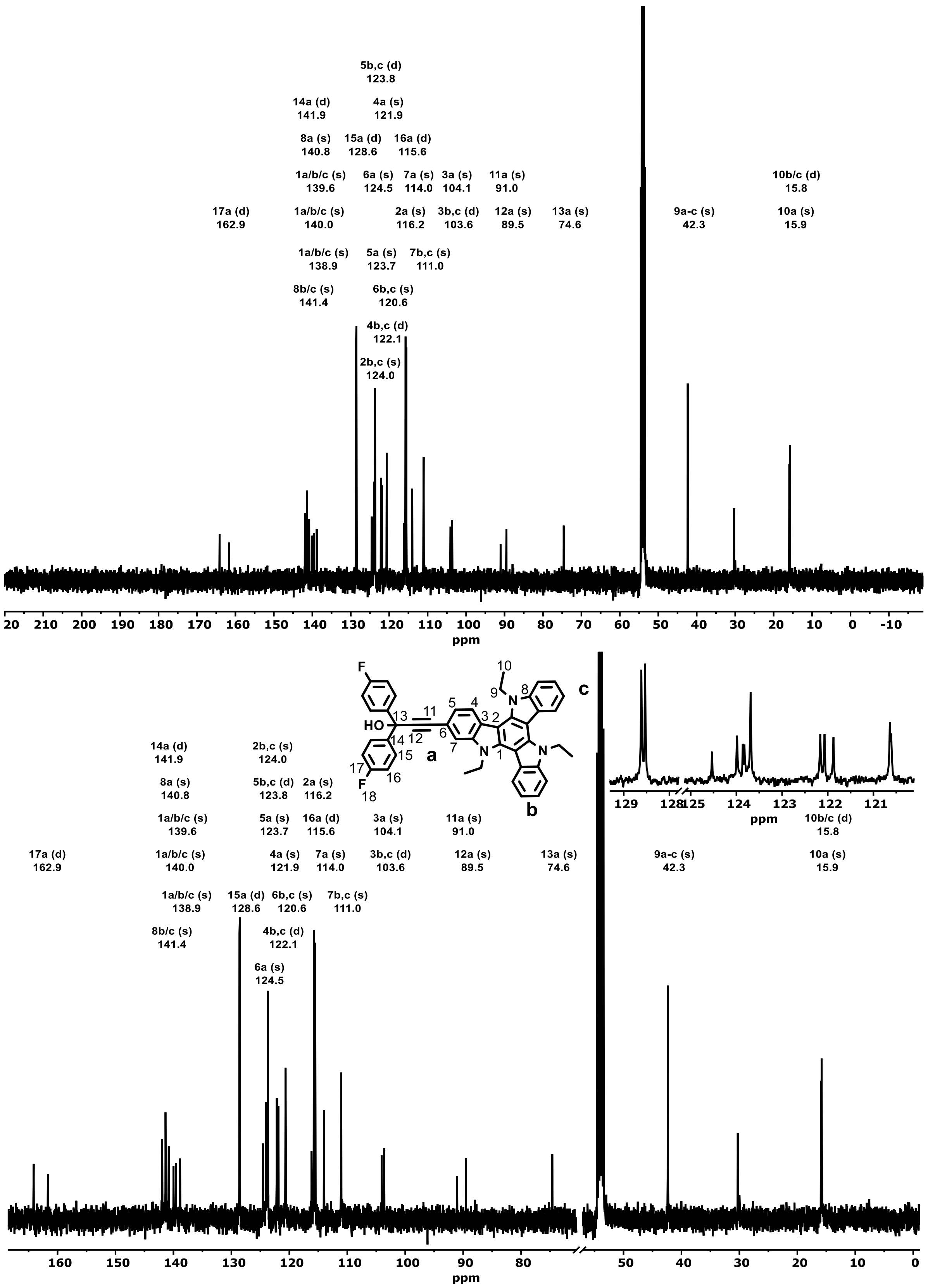

Figure S8. $^{13}C\{^{1}H\}$-NMR spectrum of **2-OH** in $CDCl_3$ (101 MHz, 300 K).

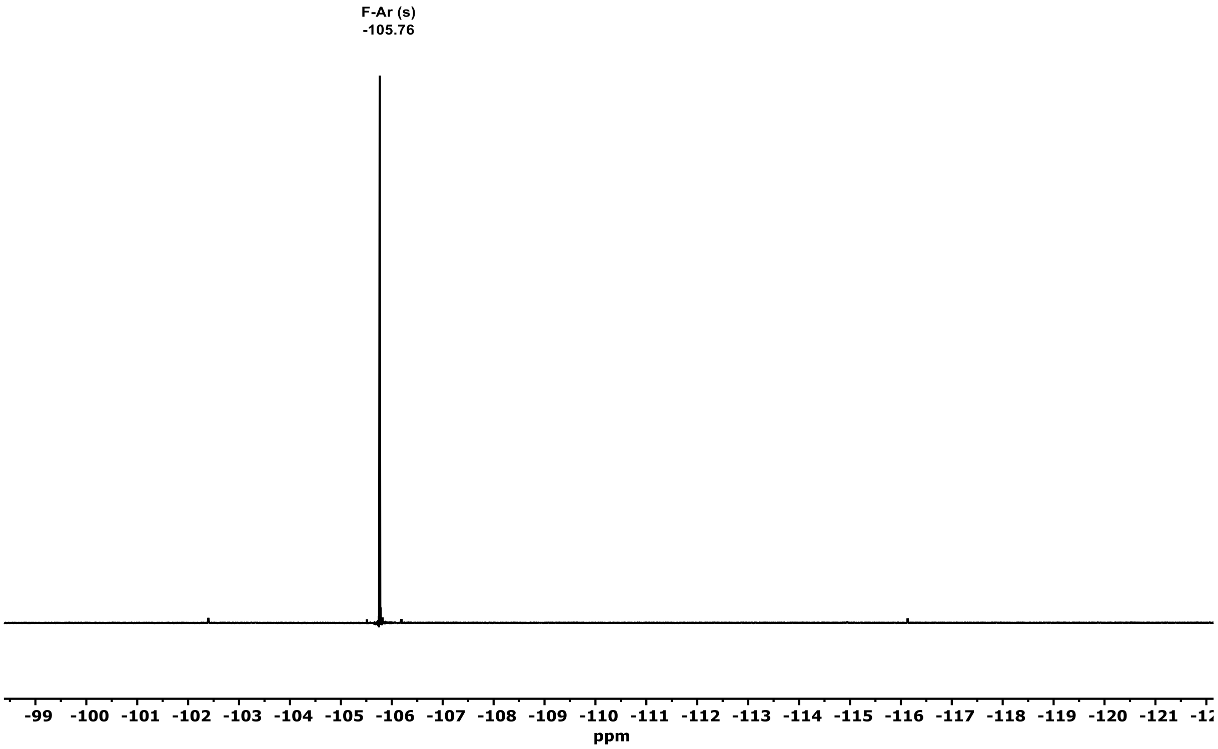

Figure S9. ^{19}F-NMR spectrum of **2-OH** in $CDCl_3$ (376 MHz, 300 K).

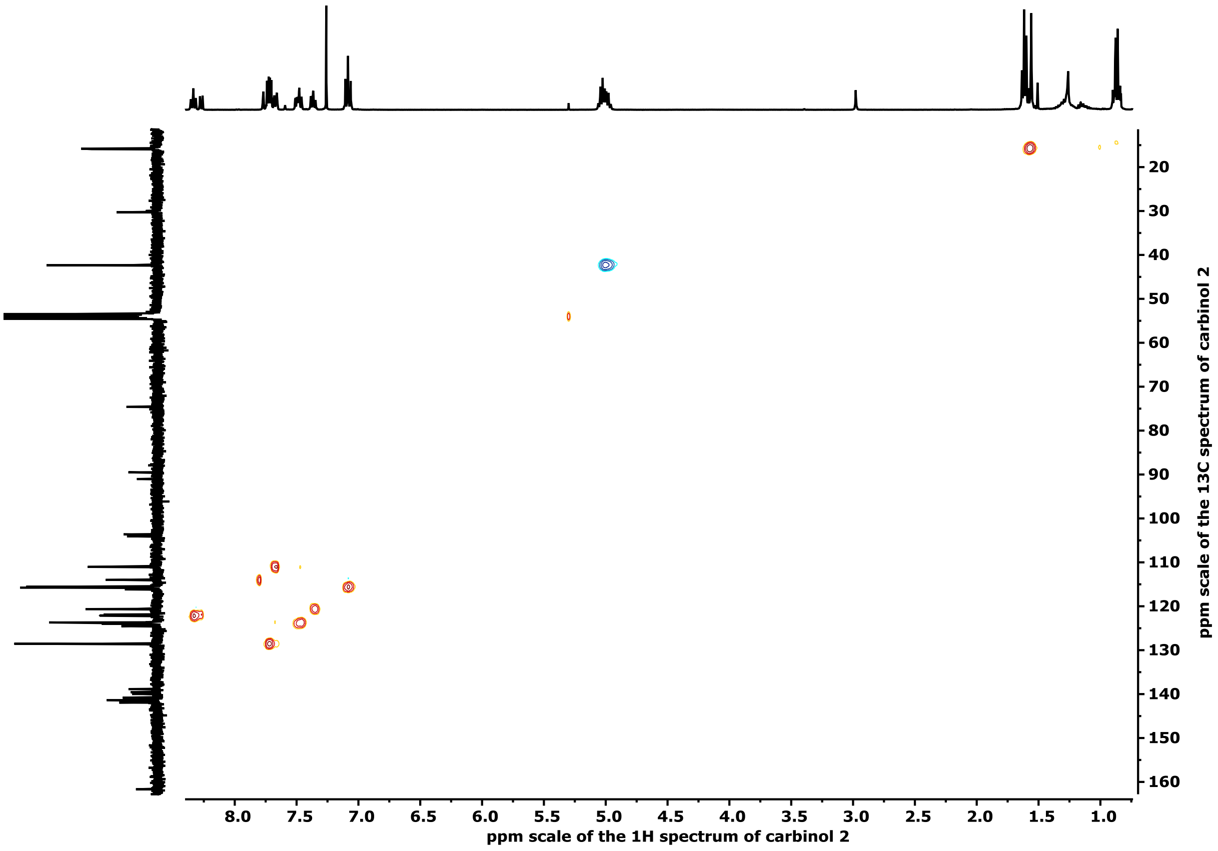

Figure S10. 2D HSQC spectrum of **2-OH** in $CDCl_3$ (^{1}H: 400 MHz, ^{13}C{^{1}H}: 101 MHz, 300 K).

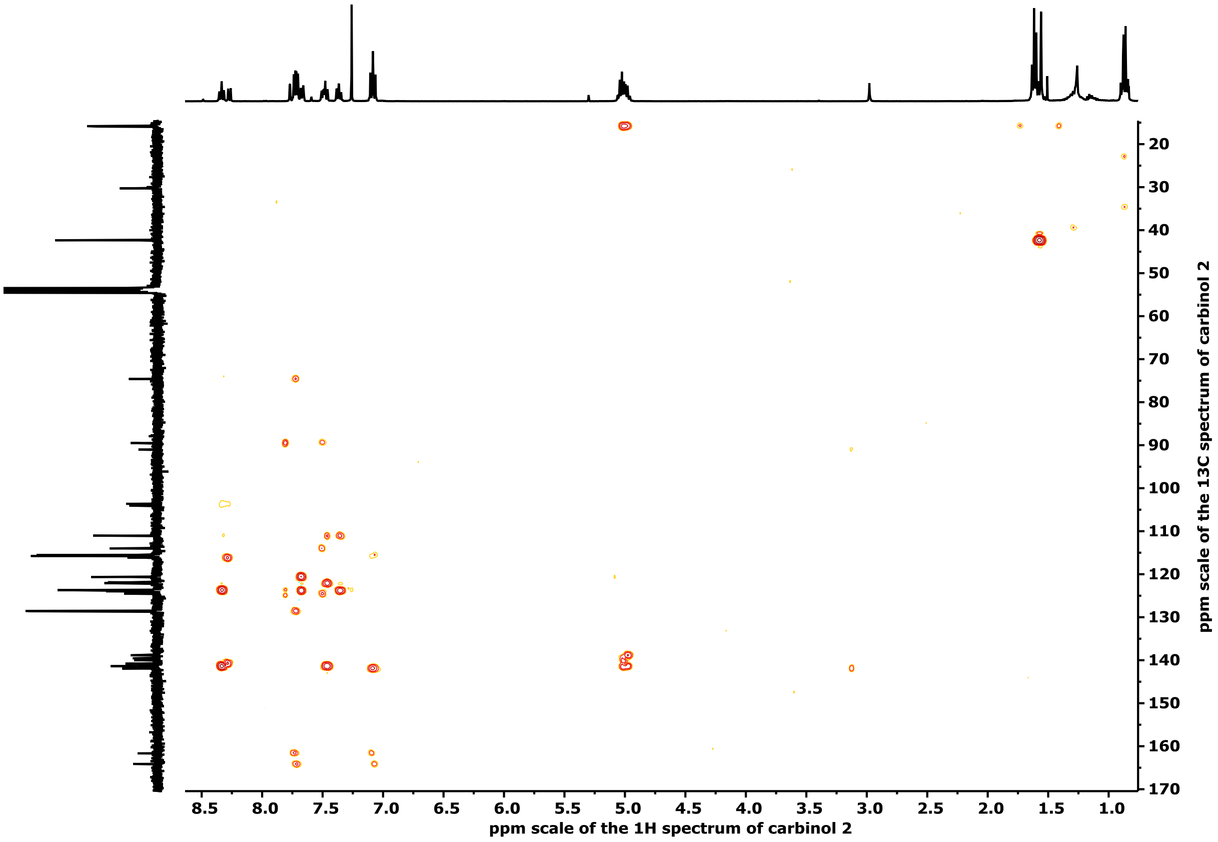

Figure S11. 2D HMBC spectrum of **2-OH** in $CDCl_3$ (^1H: 400 MHz, $^{13}C\{^1H\}$: 101 MHz, 300 K).

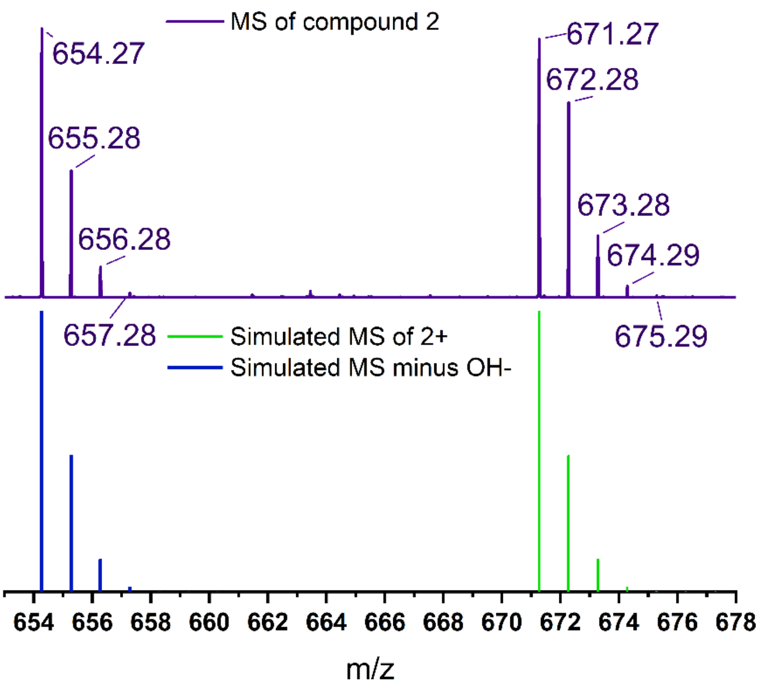

Figure 12. Molecular ion peaks of compound $\{\mathbf{2}+H\}^+$ and $\{\mathbf{2}–OH\}^+$ in the ESI mass spectrum (CH_2Cl_2). The bottom panel displays the calculated masses and their isotopic distributions.

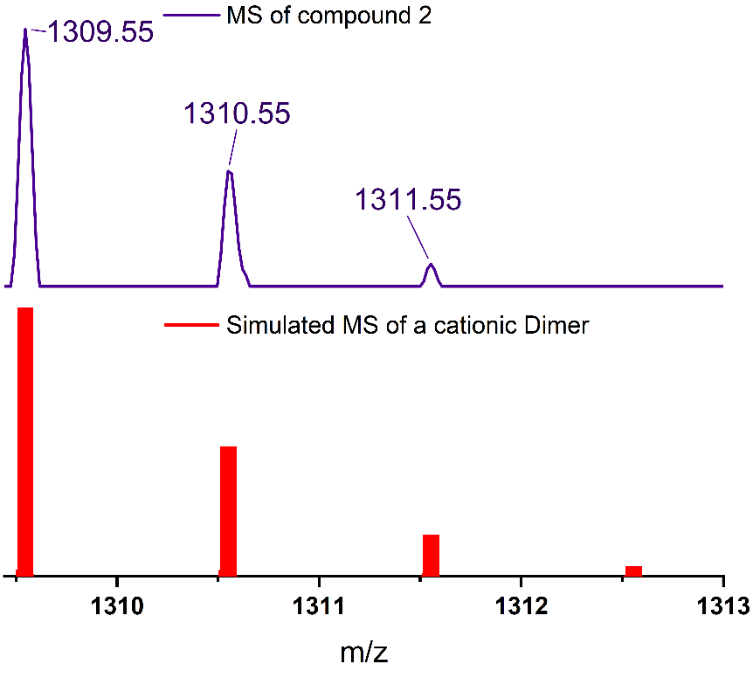

Figure 13. Molecular ion peaks of dimer {**2-2**+H}$^+$ in the ESI mass spectrum (CH_2Cl_2). The calculated mass and the isotopic distributions are shown as red bars in the bottom panel.

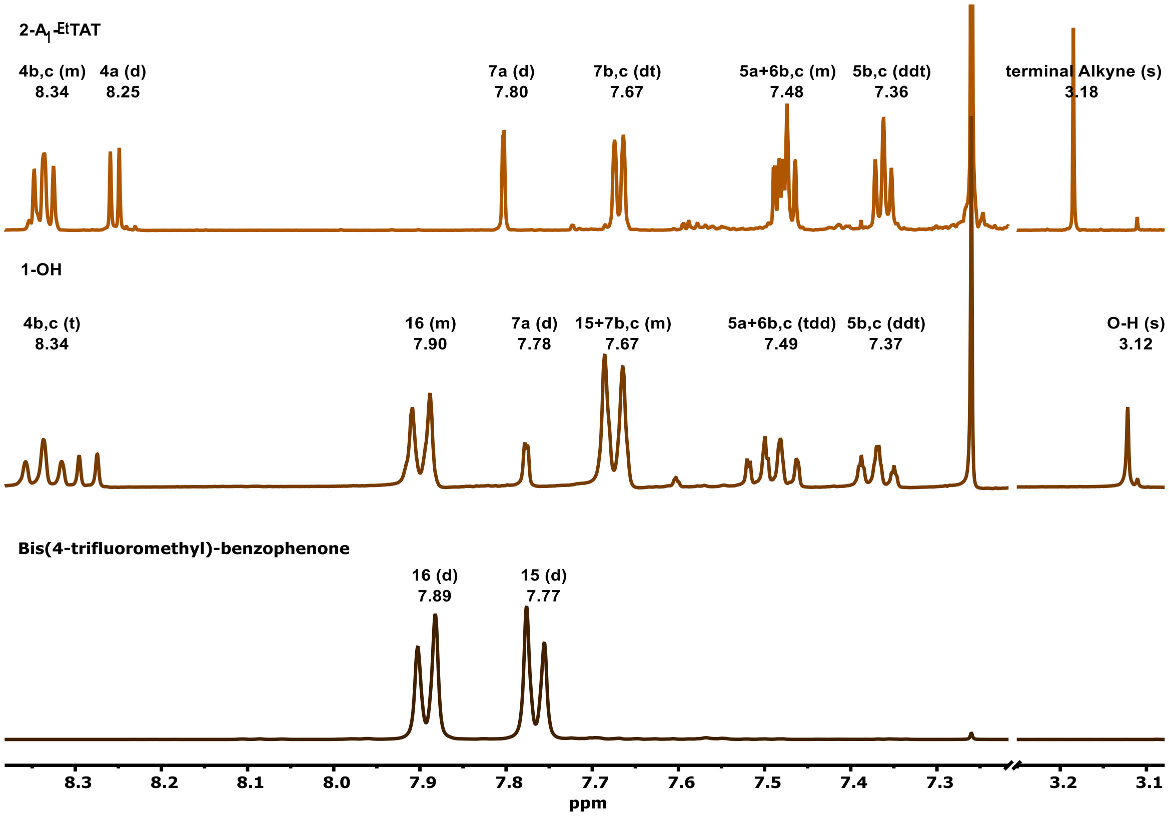

Figure S14. ^1H-NMR ($CDCl_3$, 400 MHz, 300 K) signals in the region of aryl protons and the characteristic alkyne and O–H proton resonances of the precursor molecules **2-A$_1$-^{Et}TAT** (top row), Bis-(4-trifluoromethyl)-benzophenone (bottom row) and compound **1-OH** (middle row).

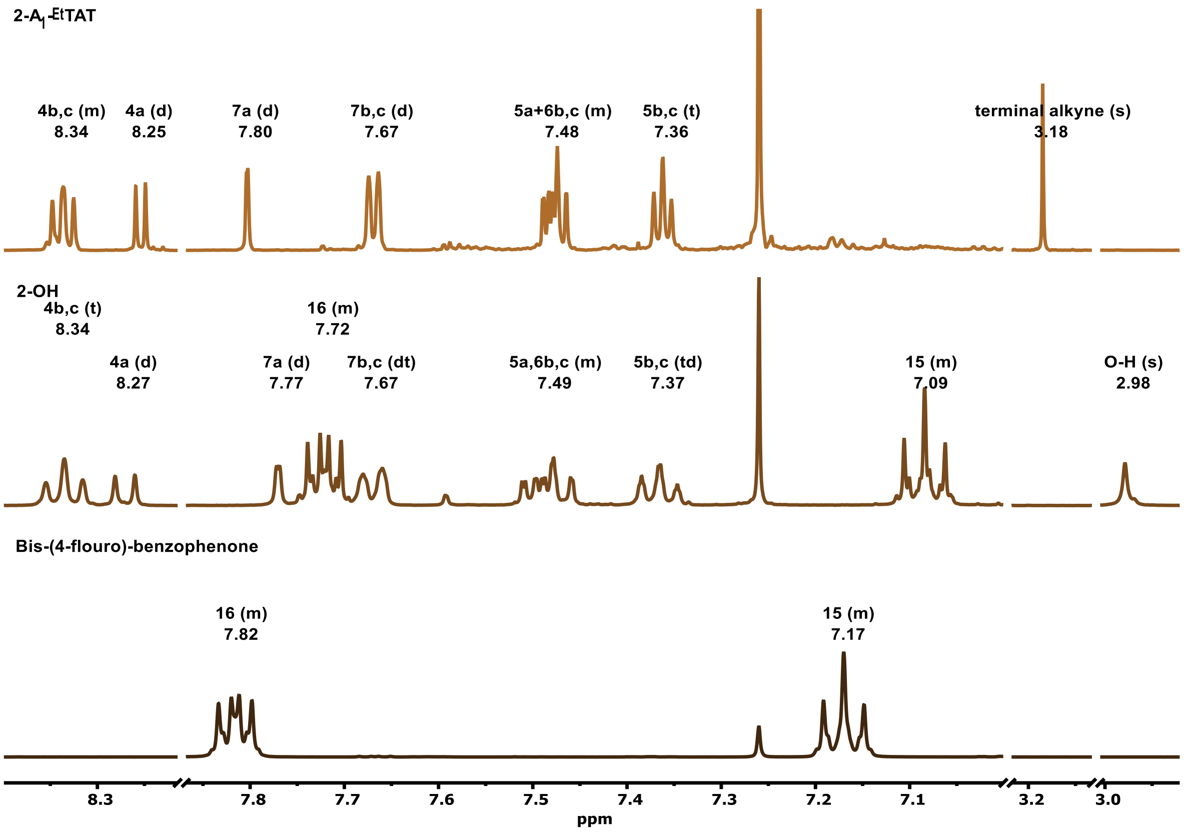

Figure S15. ^{1}H-NMR ($CDCl_3$, 400 MHz, 300 K) signals in the region of aryl protons and the characteristic alkyne and O–H proton resonances of the precursor molecules **2-A$_1$-^{Et}TAT** (top row), bis(4-fluoro)-benzophenone (bottom row), and compound **2-OH** (middle row).

Voltammetric measurements

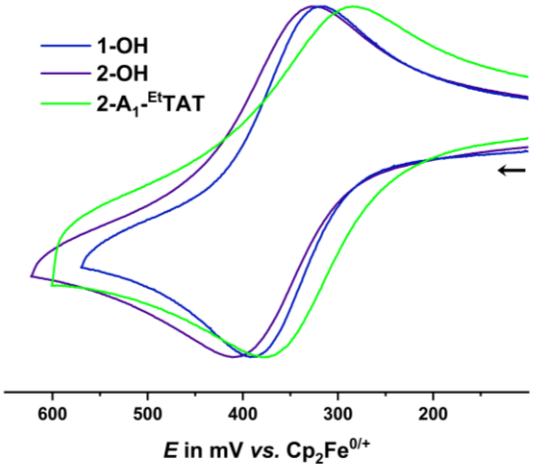

Figure S16. Cyclic voltammograms of the first oxidation of **1-OH** (c = 0.4 mM), **2-OH** (c = 0.8 mM) in CH_2Cl_2/NBu_4^+ $[BAr^{F24}]^-$ (0.04 M) and **2-A$_1$-^{Et}TAT** (c = 0.4 mM) in CH_2Cl_2/NBu_4^+ $[PF_6]^-$ (0.06 M) at a scan rate v of 100 mV/s at r. t. (**1-OH**: starting at –0.2 V, switching at 0.57 V, **2-OH**: starting at 0 V, switching at 0.62 V, **2-A$_1$-^{Et}TAT**: starting at 0 V, switching at 0.6 V; all scans in oxidative direction).

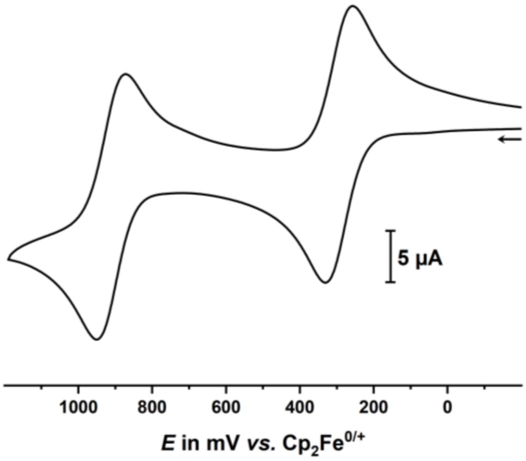

Figure S17. Cyclic voltammogram of **^{Et}TAT** (c = 4.0 mM, v = 100 mV/s, CH_2Cl_2, 0.06 M NBu_4PF_6, r. t., starting at –0.2 V in oxidative direction, switching at 1.15 V).[10]

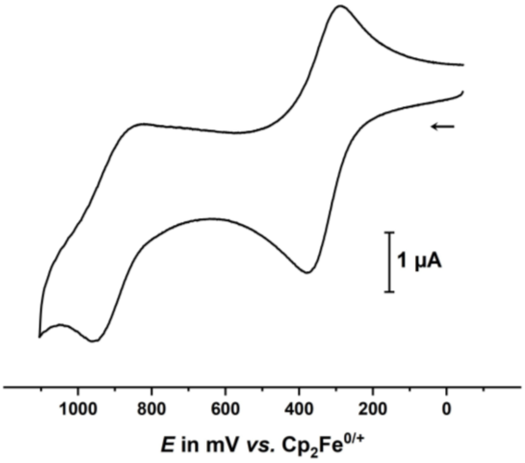

Figure S18. Cyclic voltammogram of **2-A$_1$-^{Et}TAT** (c = 0.4 mM, v = 100 mV/s, CH_2Cl_2, 0.06 M NBu_4PF_6, r. t.,starting at –0.02 V in oxidative direction, switching at 1.12 V).[10]

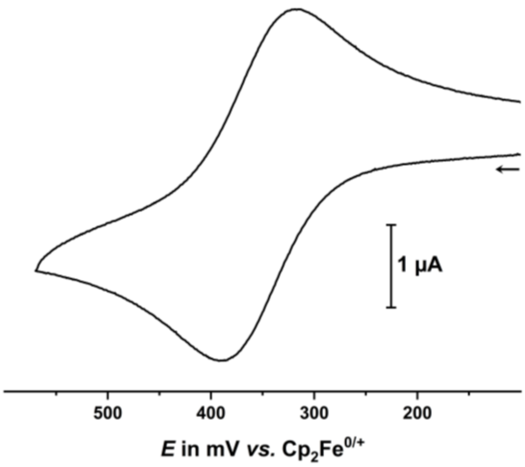

Figure S19. Cyclic voltammogram of **1-OH** (c = 0.4 mM, v = 100 mV/s, CH_2Cl_2, 0.04 M NBu_4^+ $[BArF^{24}]^-$, r. t., starting at –0.2 V in oxidative direction, switching at 0.57 V).

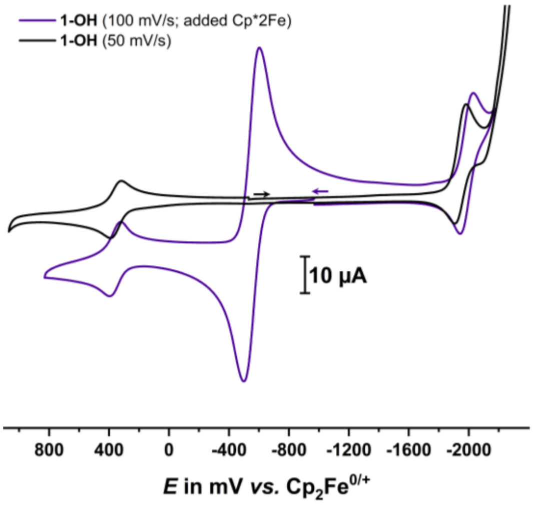

Figure S20. Full cyclic voltammograms of **1-OH** (c = 1.0 mM). Purple: v = 100 mV/s, CH_2Cl_2, 0.04 M NBu_4^+ $[BArF^{24}]^-$, r. t., with the added redox standard Cp_2^*Fe ($E_{1/2}$ = –550 mV, starting at –0.97 V in oxidative direction, switching at 0.83 V , starting at –0.97 V in reductive direction, switching at –2.17 V and at –2.170 V; black without the standard at v = 50 mV/s, in CH_2Cl_2, 0.04 M NBu_4^+ $[BArF^{24}]^-$, r. t., starting at –0.53 V in reductive direction, switching at –2.17 V and at 1.07 mV.

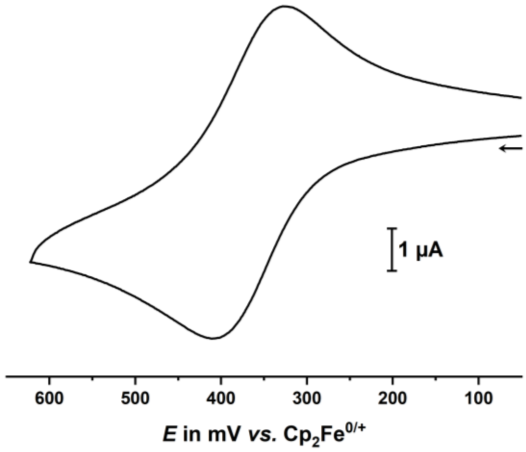

Figure S21. Cyclic voltammogram of the first oxidation of **2-OH** (c = 0.8 mM, v = 100 mV/s, CH_2Cl_2, 0.04 M NBu_4^+ $[BAr^{F24}]^-$, r. t., starting at 0 V in oxidative direction, switching at 0.62 V).

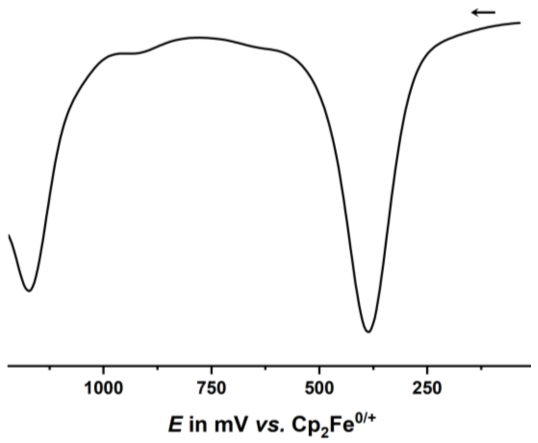

Figure S22. Square wave voltammogram of **2-OH** in CH_2Cl_2/NBu_4^+ $[BAr^{F24}]^-$ (0.04 M) at r. t. (*c* = 0.8 mM, frequency = 15 Hz, step height = 4 mV, square wave amplitude = 25 mV, starting at 0 V in oxidative direction and a final potential of 1.4 V).

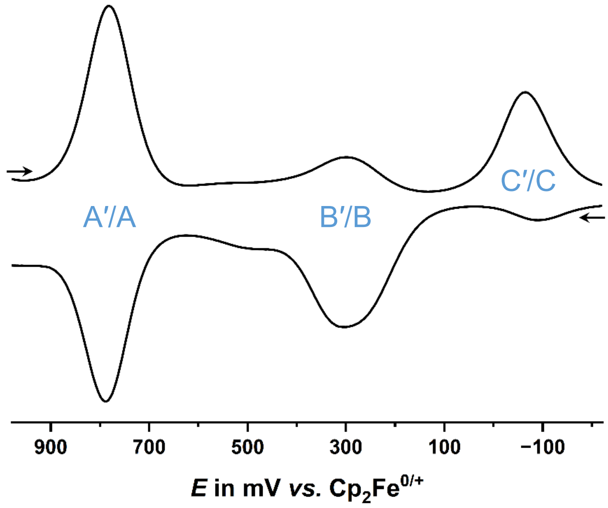

Figure S23. Oxidative and reductive scan of the square wave voltammogram of cation **1⁺** (*c* = 0.4 mM) in CH_2Cl_2/NBu_4^+ $[BAr^{F24}]^-$ (0.04 M) at r. t. (frequency = 15 Hz, step height = 4 mV, square wave amplitude = 25 mV, top: starting at 0.87 V in reductive direction with a final potential of –1.11 V, top: starting at –1.11 V in oxidative direction with a final potential of 0.87 V).

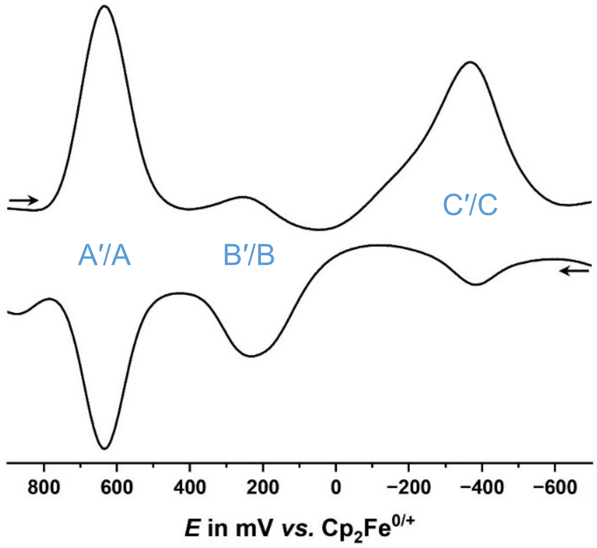

Figure S24. Oxidative and reductive scan of the square wave voltammogram of cation **2⁺** (*c* = 0.8 mM) in CH_2Cl_2/NBu_4^+ $[BAr^{F24}]^-$ (0.04 M) at r. t. (frequency = 15 Hz, step height = 4 mV, square wave amplitude = 25 mV, top: starting at 1.03 V in reductive direction with a final potential of –1.11 V, top: starting at –1.11 V in oxidative direction with a final potential of 1.03 V).

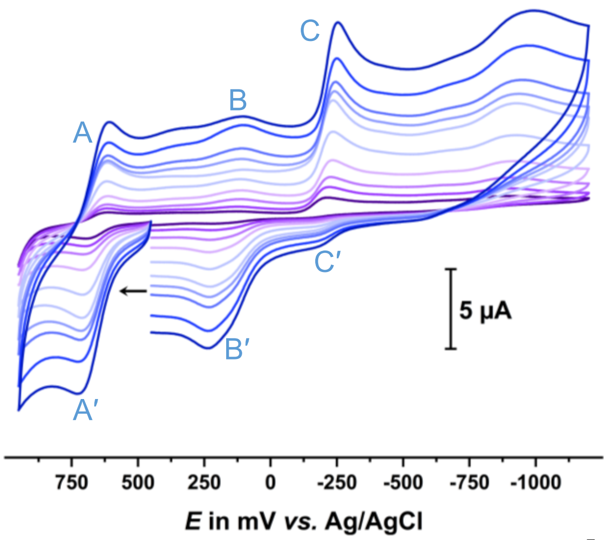

Figure S25. Cyclic voltammograms of cation **1⁺** (c = 0.9 mM) in CH_2Cl_2/NBu_4^+ $[BAr^{F24}]^-$ (0.04 M) at r. t. at different scan rates initiated from a starting potential slightly negative of the onset of the TAT oxidation, peak A' (v = 25, 50, 100, 200, 400, 600, 800, 1000, 1500 and 2000 mV/s, from purple to blue, starting at 0.45 V in oxidative direction, switching at 0.95 V and at –1.2 V). For the assignment of peaks, see Figure 6 of the manuscript.

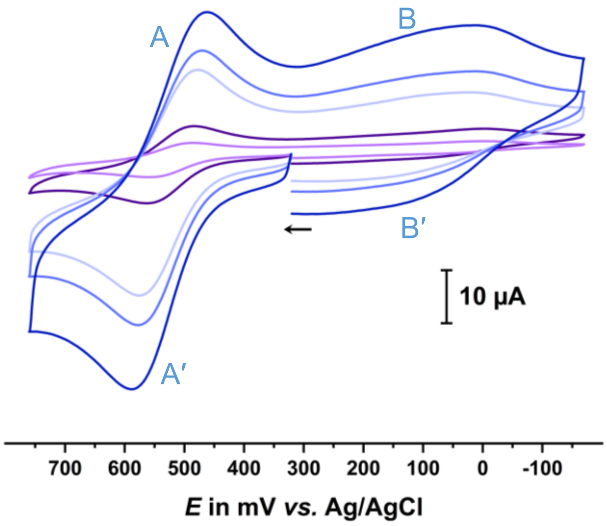

Figure S26. Cyclic voltammograms of cation **1⁺** (c = 0.9 mM) in CH_2Cl_2/NBu_4^+ $[BAr^{F24}]^-$ (0.04 M) at r. t. at different scan rates initiated from a starting potential slightly negative of the onset of TAT oxidation, peak A', with clipping of the reverse scan after traversing peak B (v = 25, 50, 100, 600, 1000, 1500 and 2000 mV/s, from purple to blue, starting at 0.32 V in oxidative direction, switching at 0.76 V and at –0.17 V). For the assignment of peaks, see Figure 6 of the manuscript.

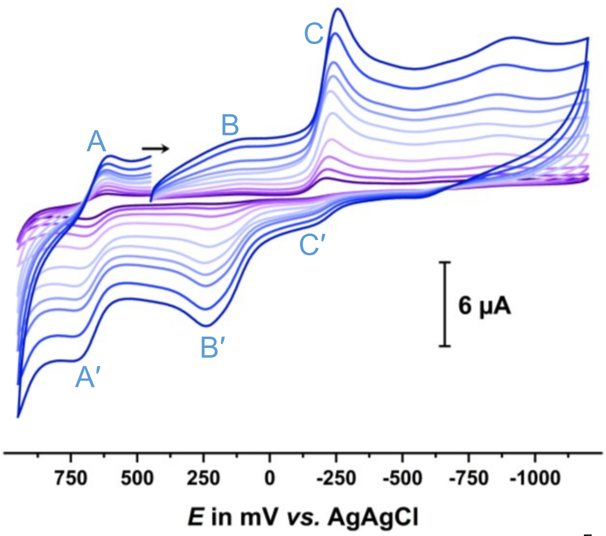

Figure S27. Cyclic voltammograms of cation $\mathbf{1}^+$ (c = 0.9 mM) in CH_2Cl_2/NBu_4^+ $[BAr^{F24}]^-$ (0.04 M) at r. t. at different scan rates. The sweep is initiated slightly positive of peak B for reduction of dimer $\mathbf{1}^{+\bullet}$-$\mathbf{1}^{+\bullet}$ (v = 25, 50, 100, 200, 400, 600, 800, 1000, 1500 and 2000 mV/s, from purple to blue, starting at 0.45 V in reductive direction, switching at –1.2 V and at 0.95 V). For the assignment of peaks, see Figure 6 of the manuscript.

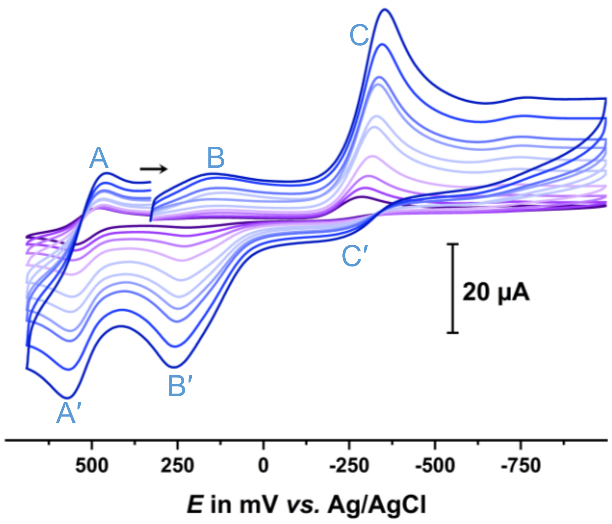

Figure S28. Cyclic voltammograms of cation $\mathbf{2}^+$ (c = 0.6 mM) in CH_2Cl_2/NBu_4^+ $[BAr^{F24}]^-$ (0.04 M) at r. t. at different scan rates, with the sweep initiated slightly positive of peak B for reduction of dimer $\mathbf{2}^{+\bullet}$-$\mathbf{2}^{+\bullet}$ (v = 25, 50, 100, 200, 400, 600, 800, 1000, 1500 and 2000 mV/s, from purple to blue, starting at 0.33 V in reductive direction, switching at –1.0 V and at 0.69 V). For the assignment of peaks, see Figure 6 of the manuscript.

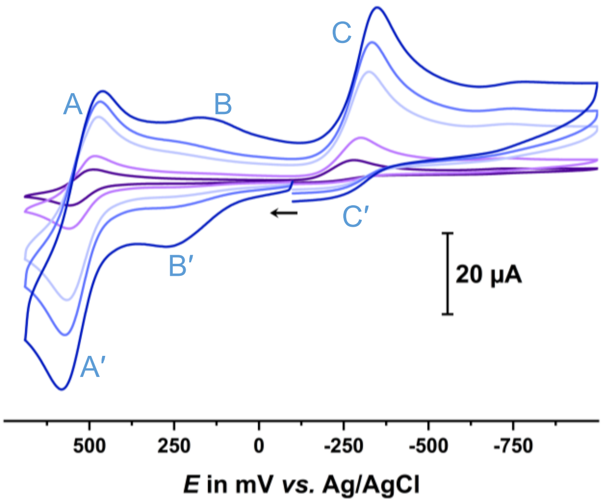

Figure S29. Cyclic voltammograms of cation $\mathbf{2^+}$ (c = 0.6 mM) in CH_2Cl_2/NBu_4^+ $[BAr^{F24}]^-$ (0.04 M) at r. t. at different scan rates, with the anodic sweep initiated slightly negative of peak B′ for oxidation of dimer $\mathbf{2^{+\bullet}}$-$\mathbf{2^{+\bullet}}$ (v = 25, 100, 600, 1000 and 2000 mV/s, from purple to blue, starting at –0.1 V in oxidative direction, switching at 0.69 V and at –1.0 V). For the assignment of peaks, see Figure 6 of the manuscript.

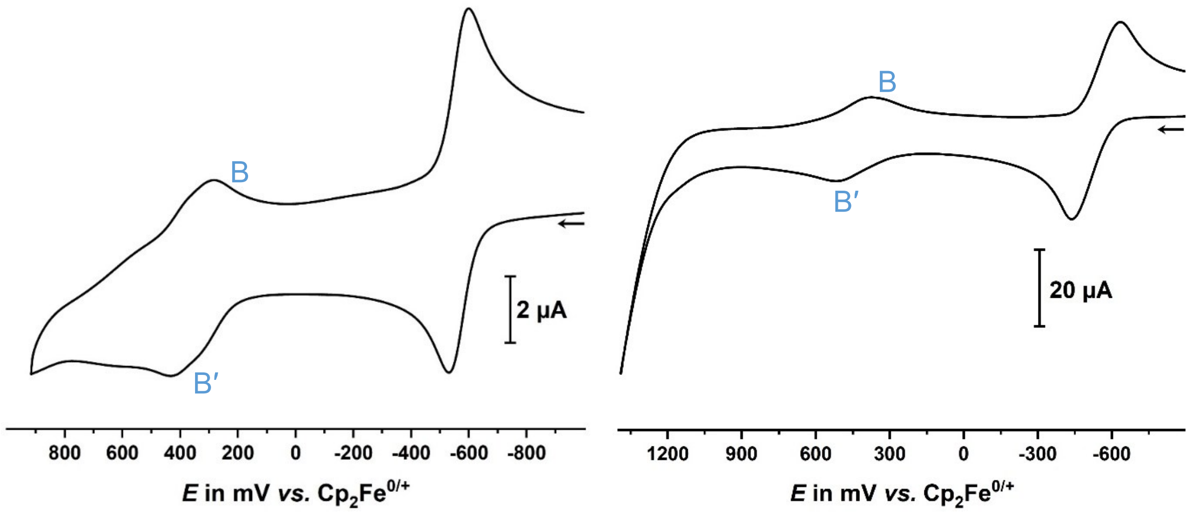

Figure S30. Cyclic voltammograms of chemically generated **1-1** obtained subsequent to adding an excess of $FeCp^*_2$ ($E_{1/2}$ = –550 mV) to $\mathbf{1^+}$ (c = 0.9 mM). The sweep was initiated from negative of the oxidation potential of $FeCp^*_2$ and shows, apart from the redox wave of Cp^*_2Fe, exclusively wave B′/B assigned to the oxidation of formed dimer **1-1**. (v = 600 mV/s, CH_2Cl_2, 0.04 M NBu_4^+ $[BAr^{F24}]^-$, r. t., left: starting at –1.78 V in oxidative direction, switching at 0.92 V; right: starting at –1.0 V in oxidative direction, switching at 1.40 V). For the assignment of peaks, see Figure 6 of the manuscript.

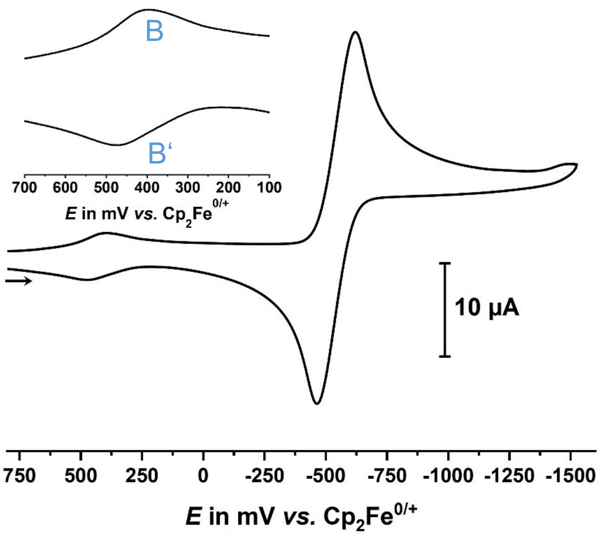

Figure S31. Cyclic voltammogram (v = 50 mV/s, CH_2Cl_2, 0.04 M NBu_4^+ $[BAr^{F24}]^-$, r. t., starting at 1.03 V in reductive direction, switching at –1.53 V) of chemically generated **1-1** obtained by adding an excess of $FeCp^*_2$ ($E_{1/2}$ = -550 mV) to **1⁺** (c = 0.9 mM). The sweep was initiated from positive of the oxidation of the dimer to the associated dication **1⁺-1⁺**. An equilibration time of 300 s after applying the starting potential to the working electrode and before the sweep was initiated. The voltammogram shows wave B/B′ for the reduction of the oxidized dimer. This shows, that the oxidized dimer persists over longer periods of time. For the assignment of peaks, see Figure 6 of the manuscript.

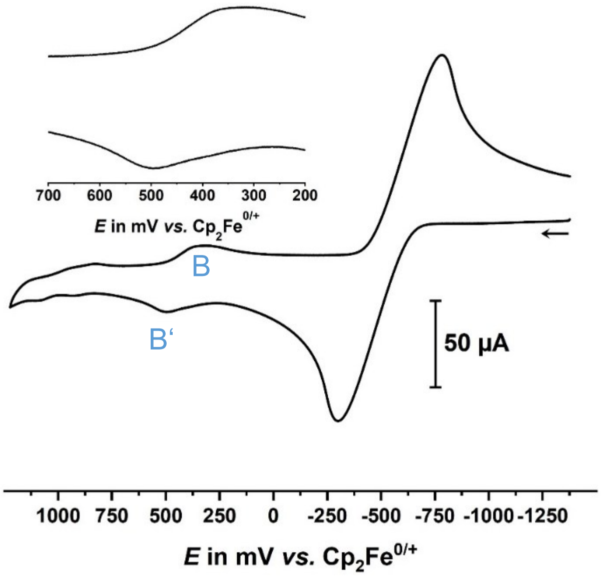

Figure S32. Cyclic voltammogram of chemically generated **2-2** obtained by adding an excess of $FeCp^*_2$ ($E_{1/2}$ = -550 mV) to **2⁺** (c = 0.8 mM). The sweep was initiated from negative of the oxidation potential of $FeCp^*_2$ and shows, apart from the redox wave of Cp^*_2Fe, wave B′/B assigned to the oxidation of formed dimer **2-2**. (v = 600 mV/s, CH_2Cl_2, 0.04 M NBu_4^+ $[BAr^{F24}]^-$, r. t., starting from –1.37 V in oxidative direction, switching at 1.22 V). For the assignment of peaks, see Figure 6 of the manuscript.

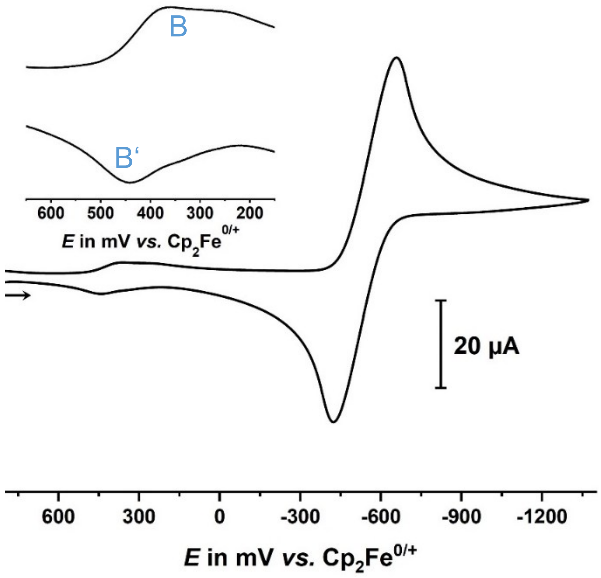

Figure S33. Cyclic voltammogram (v = 50 mV/s, CH_2Cl_2, 0.04 M NBu_4^+ $[BAr^{F24}]^-$, r. t., starting from 1.08 V in reductive direction, switching at –1.37 V) of chemically generated **2-2** obtained by adding an excess of $FeCp^*_2$ ($E_{1/2}$ = -550 mV) to **2^+** (c = 0.8 mM). The sweep was initiated from positive of the oxidation of the dimer to the associated dication **2^+-2^+**. An equilibration time of 300 s after applying the starting potential to the working electrode and before the sweep was initiated. The voltammogram shows wave B/B′ for the reduction of the oxidized dimer. This shows that the oxidized dimer persists over longer periods of time. For the assignment of peaks, see Figure 6 of the manuscript.

UV/vis/NIR and IR Spectroelectrochemistry

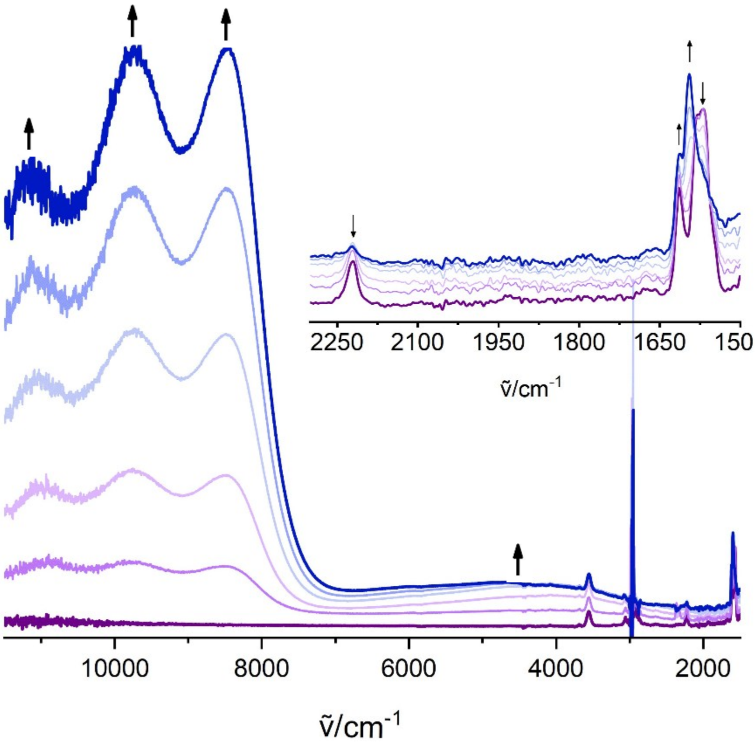

Figure S34. Changes in the IR/NIR spectrum during electrolysis of **1-OH** to **1-OH$^{+•}$** in an OTTLE cell in 1,2-$C_2H_4Cl_2$/ NBu_4^+ [BAr^{F24}]$^-$ (0.14 м) at r. t.

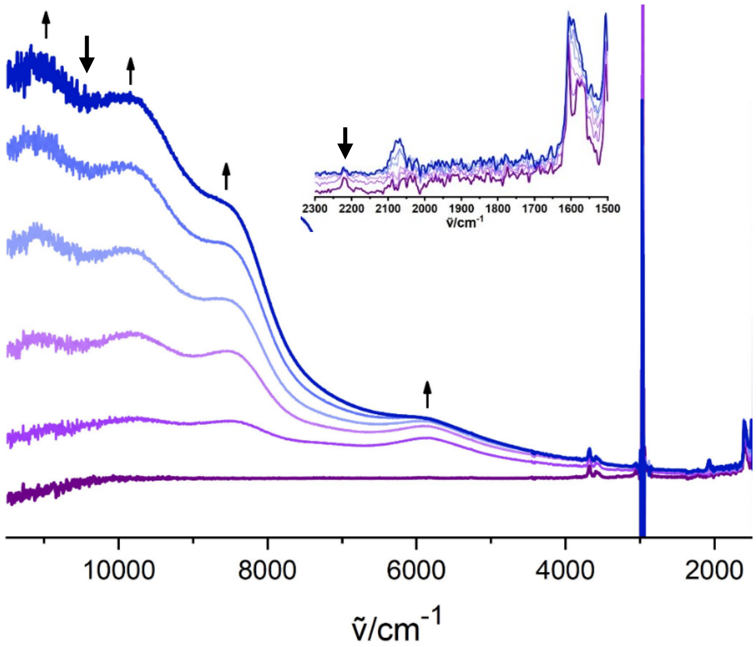

Figure S35. Changes in the IR/NIR spectrum during electrolysis of **2-OH** to **2-OH$^{+•}$** in an OTTLE cell in 1,2-$C_2H_4Cl_2$/ NBu_4^+ [BAr^{F24}]$^-$ (0.14 м) at r. t.

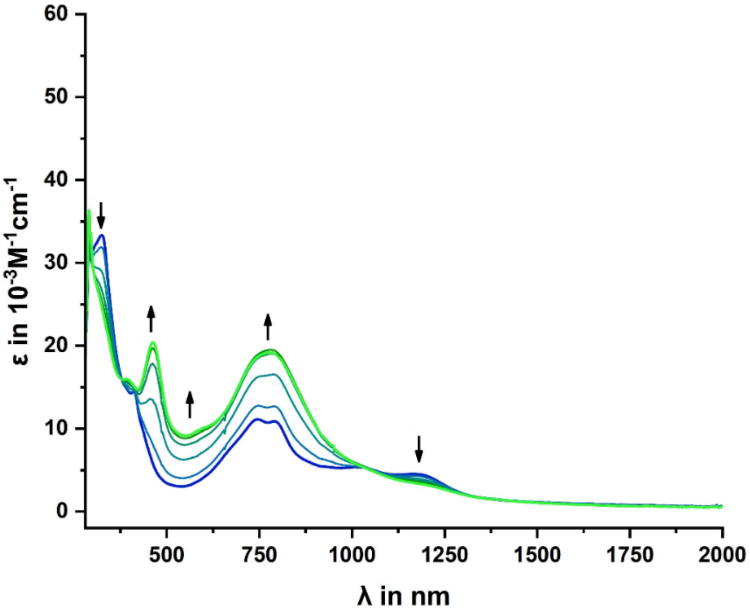

Figure S36. Changes in the UV/vis/NIR spectrum during electrolysis of **2-OH**$^{+•}$ to **2-OH**$^{2+••}$ in an OTTLE cell in $C_2H_4Cl_2$/ NBu_4^+ [BAr^{F24}]$^-$ (0.14 M) at r. t.

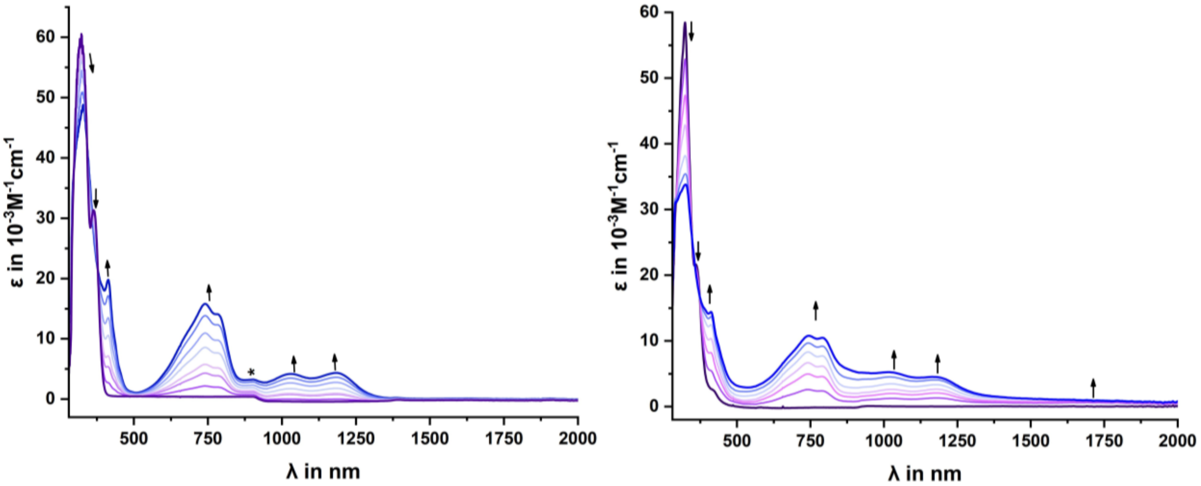

Figure S37. Changes in the UV/vis/NIR spectra of **1-OH** (left) and **2-OH** (right, purple spectra) during the first oxidation to **1-OH**$^{+•}$ and **2-OH**$^{+•}$ (blue spectra). The asterisk marks the step due to the detector switch from UV/vis to NIR detection.

EPR spectroscopy

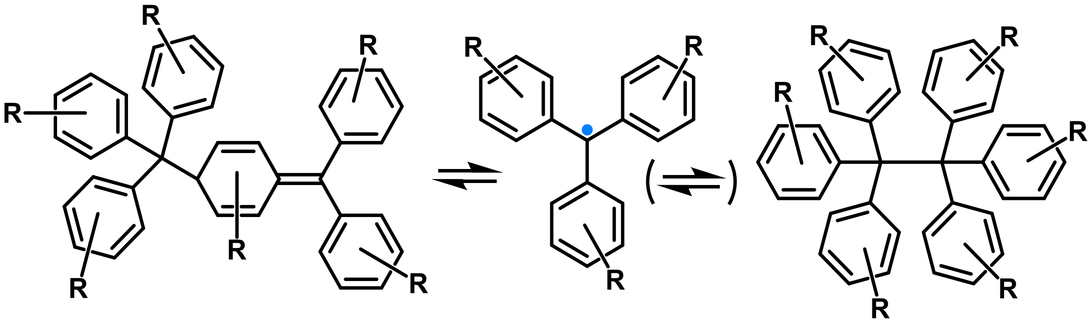

Figure S38. Possible dimerization pathways for trityl radicals. The structure on the left is the so-called Jacobsen-Nauta-dimer and the one on the right the hexaphenylethane- (HPE-) dimer. Radicals with no substituent at a *para* position usually form the Jacobsen-Nauta dimer, whereas derivatives derivatives in which all three para positions are blocked may form HPE-type dimers.

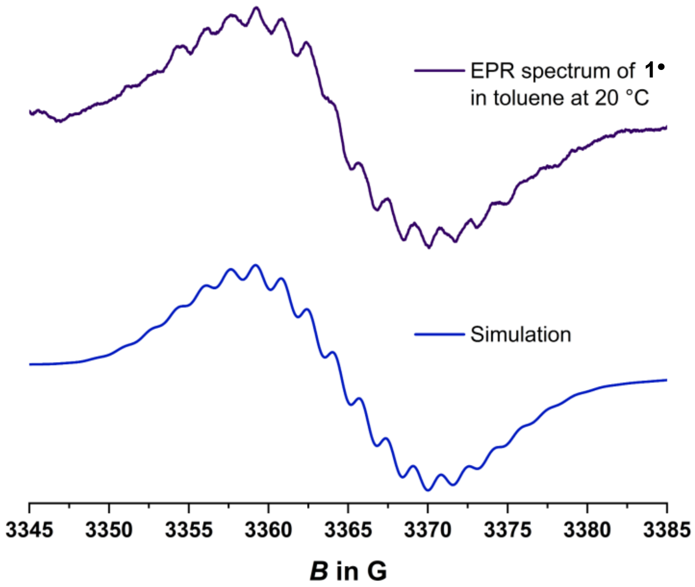

Figure S39. Experimental (purple) and simulated (blue) EPR spectra of compound **1•** in CH_2Cl_2 at r. t. The following parameters were used in the simulation: TAT(3N)-(aryl)C•(C_6H_4-4-CF_3)$_2$: *g* = 2.0038, *A*(^{19}F,six nuclei) = 11.4 MHz, *A*(^{1}H, four nuclei each) = 8.4 MHz, 1.6 MHz, *A*(^{14}N, three nuclei) = 19.0 MHz.

Quantum Chemistry

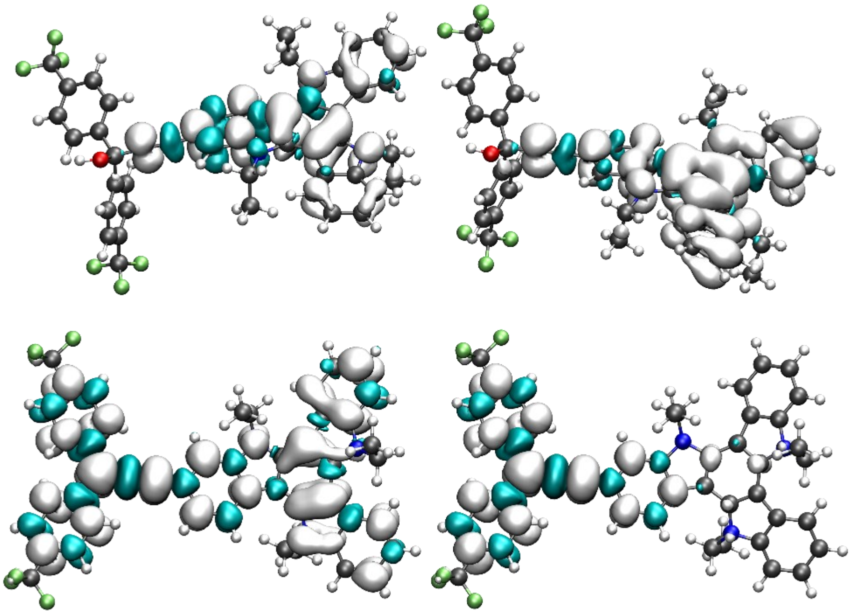

Figure S40. Computed spin density distributions of compounds **1-OH**$^{+\bullet}$ (top left), **1-OH**$^{2+\bullet\bullet}$ (triplet state, top right), **1**$^{+\bullet\bullet}$ (triplet state, bottom left), and **1**$^{\bullet}$ (bottom right).

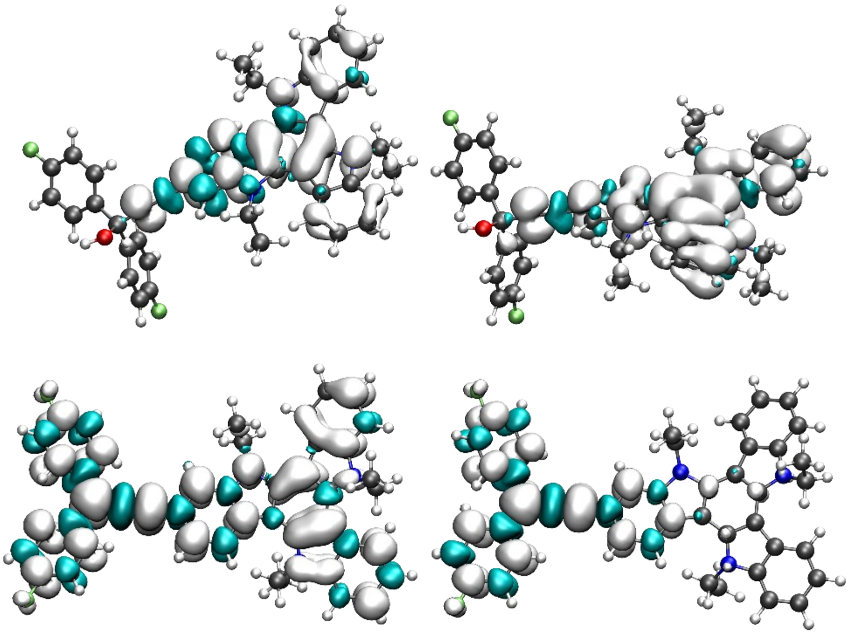

Figure S41. Computed spin density distributions of compounds **2-OH**$^{+\bullet}$ (top left), **2-OH**$^{2+\bullet\bullet}$ (triplet state, top right), **2**$^{+\bullet\bullet}$ (triplet state, bottom left), and **2**$^{\bullet}$ (bottom right)..

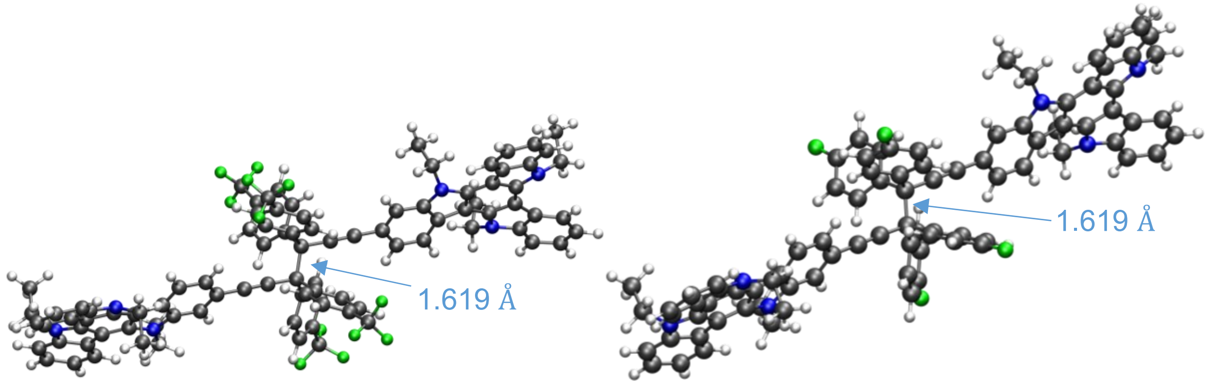

Figure S42. Calculated structures of dimers **1-1** (left) and **2-2** (right).

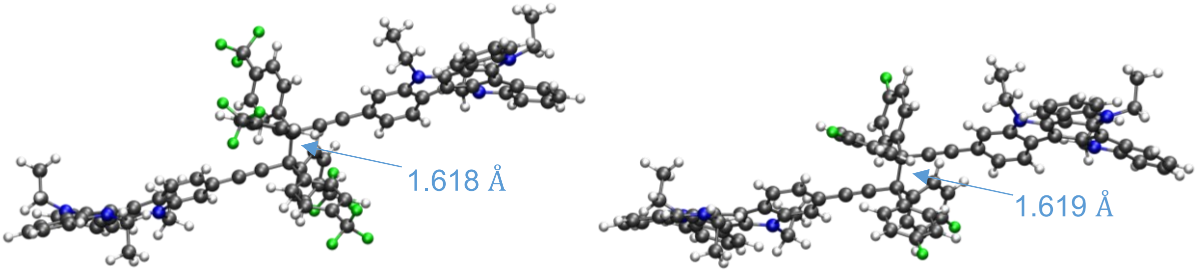

Figure S43. Calculated structures of **$1^{+\bullet}$-$1^{+\bullet}$** (left) and **$2^{+\bullet}$-$2^{+\bullet}$** (right).

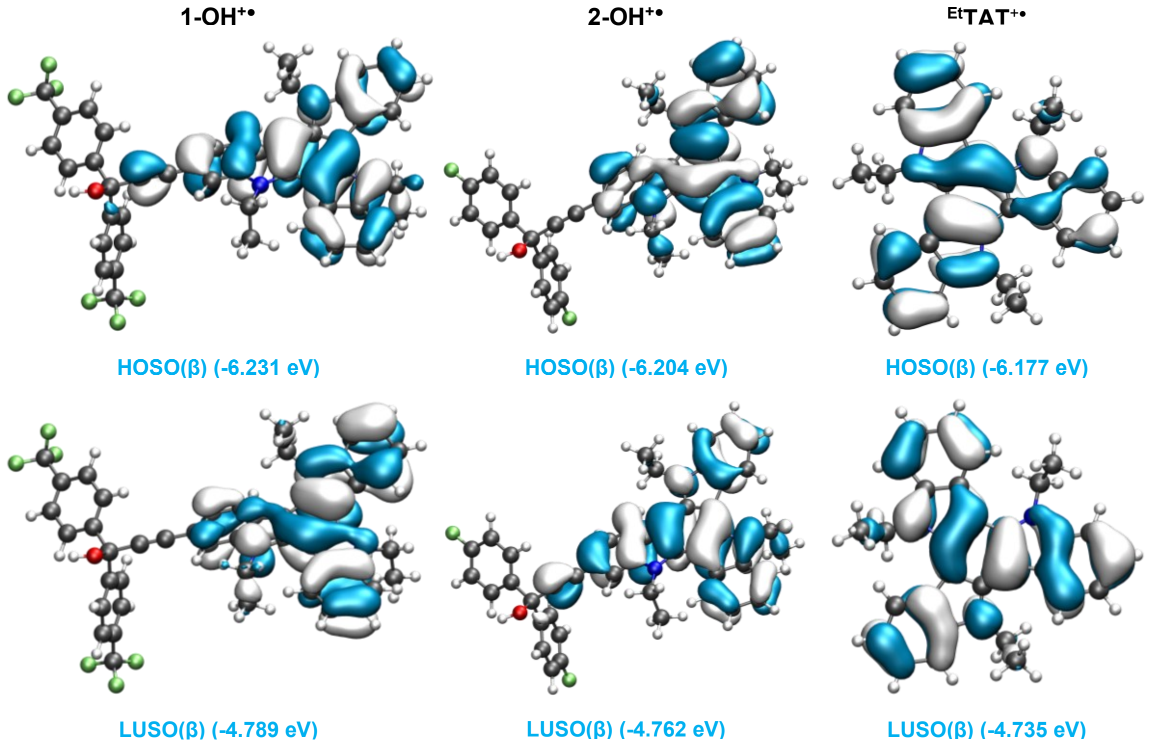

Figure S44. DFT-calculated molecular frontier orbitals of **1-OH$^{+\bullet}$** (left), **2-OH$^{+\bullet}$** (middle), and **^{Et}TAT$^{+\bullet}$** (right) with their energies.

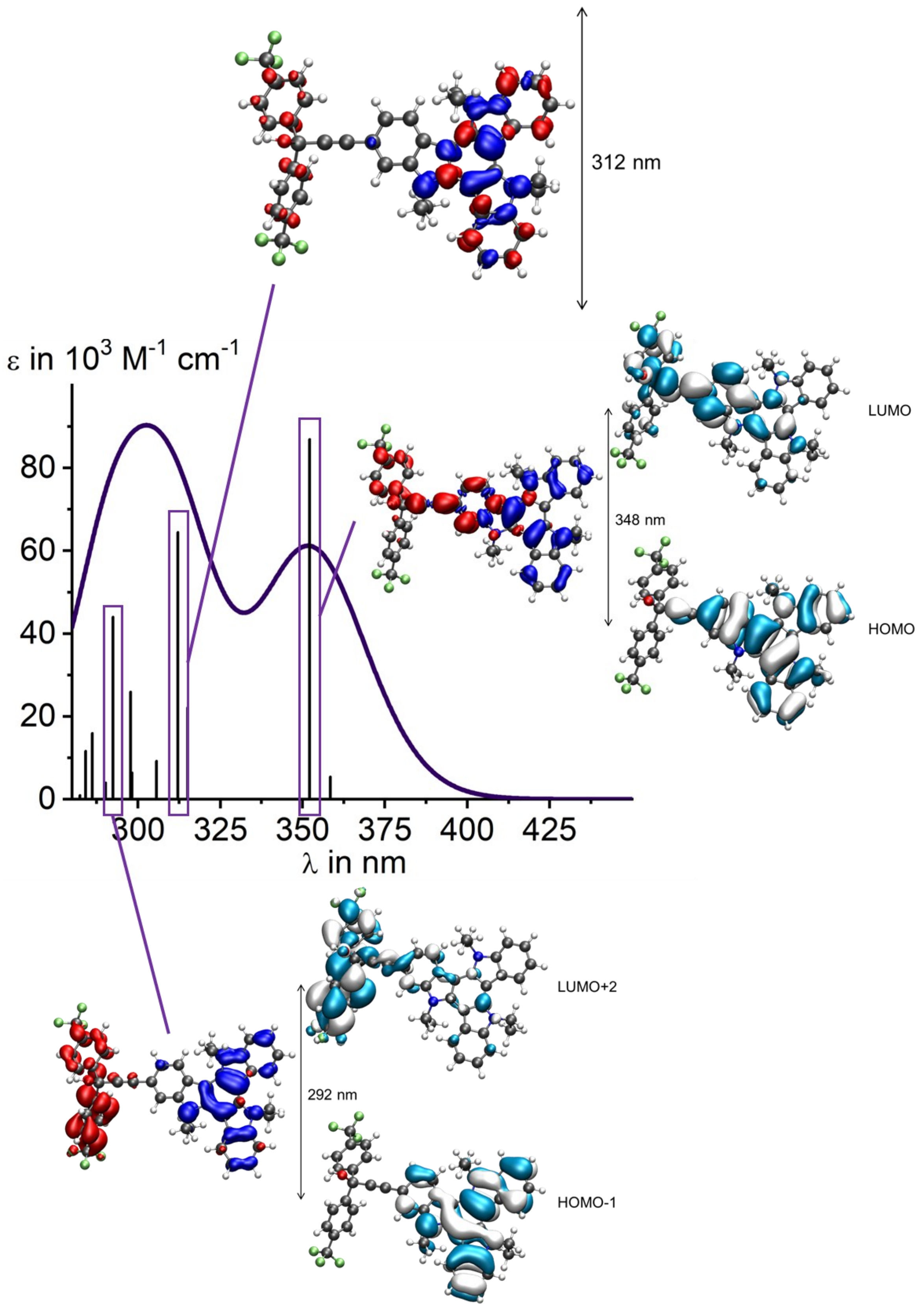

Figure S45. TD-DFT calculated UV/vis/NIR spectrum of **1-OH** with corresponding electron density difference maps (EDDMs; blue color: loss of electron density, red color: gain of electron density) for individual electronic transitions. Contributing MOs and band assignments are also provided.

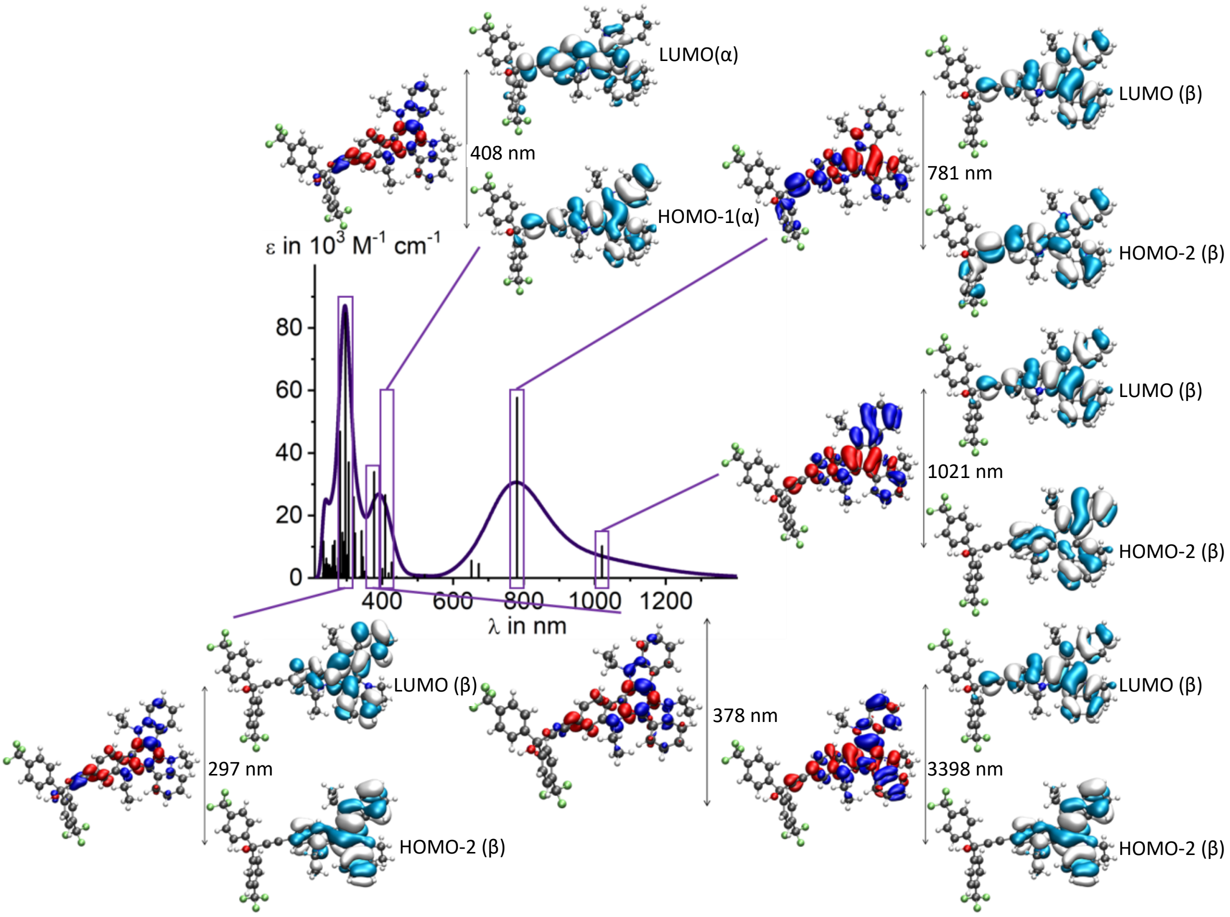

Figure S46. TD-DFT calculated UV/vis/NIR spectrum of **1-OH$^{+\bullet}$** with corresponding electron density difference maps (EDDMs; blue color: loss of electron density, red color: gain of electron density) for individual electronic transitions. Contributing MOs and band assignments are also provided.

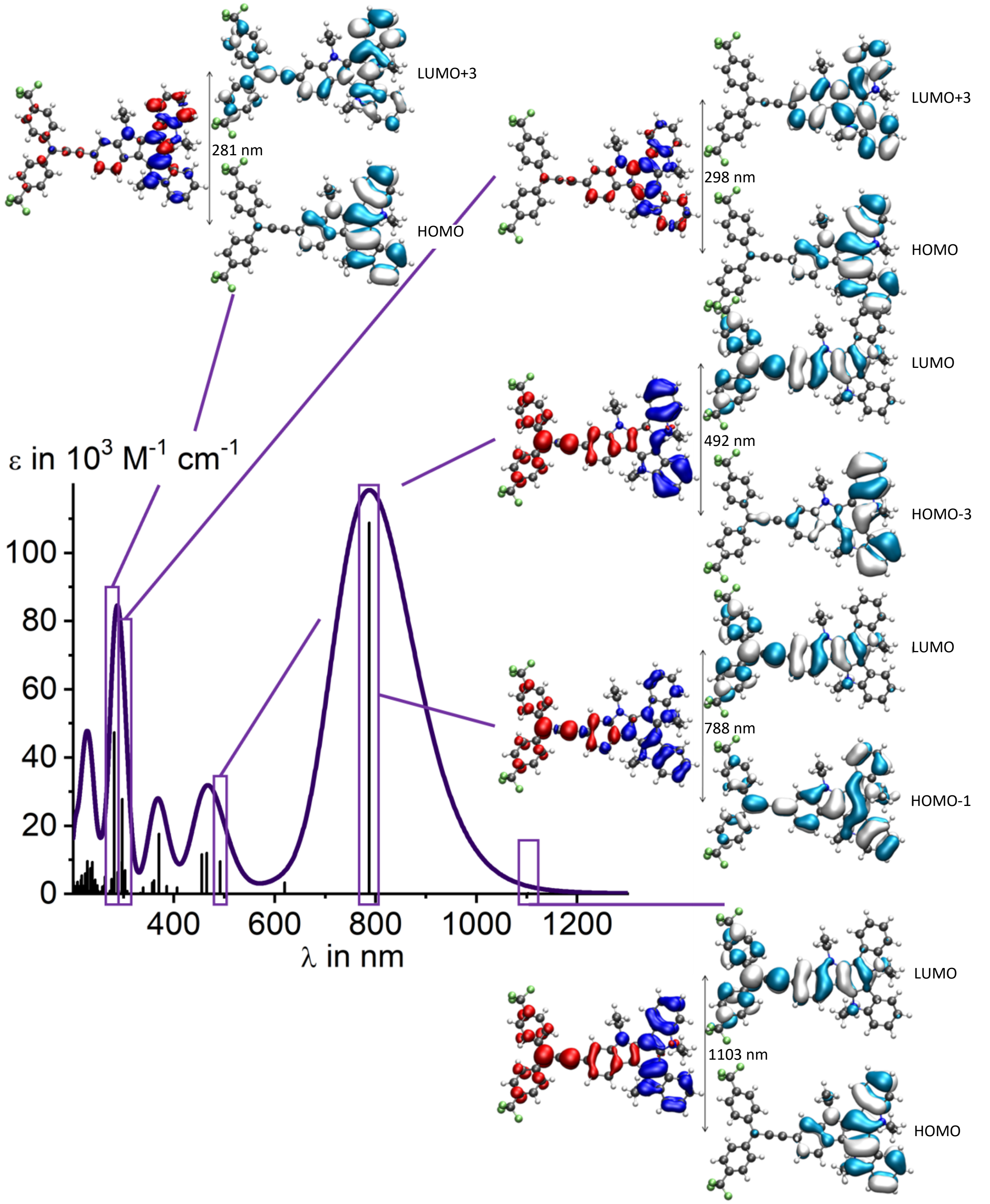

Figure S47. TD-DFT calculated UV/vis/NIR spectrum of **1**$^{+}$ with corresponding electron density difference maps (EDDMs; blue color: loss of electron density, red color: gain of electron density) for individual electronic transitions. Contributing MOs and band assignments are also provided.

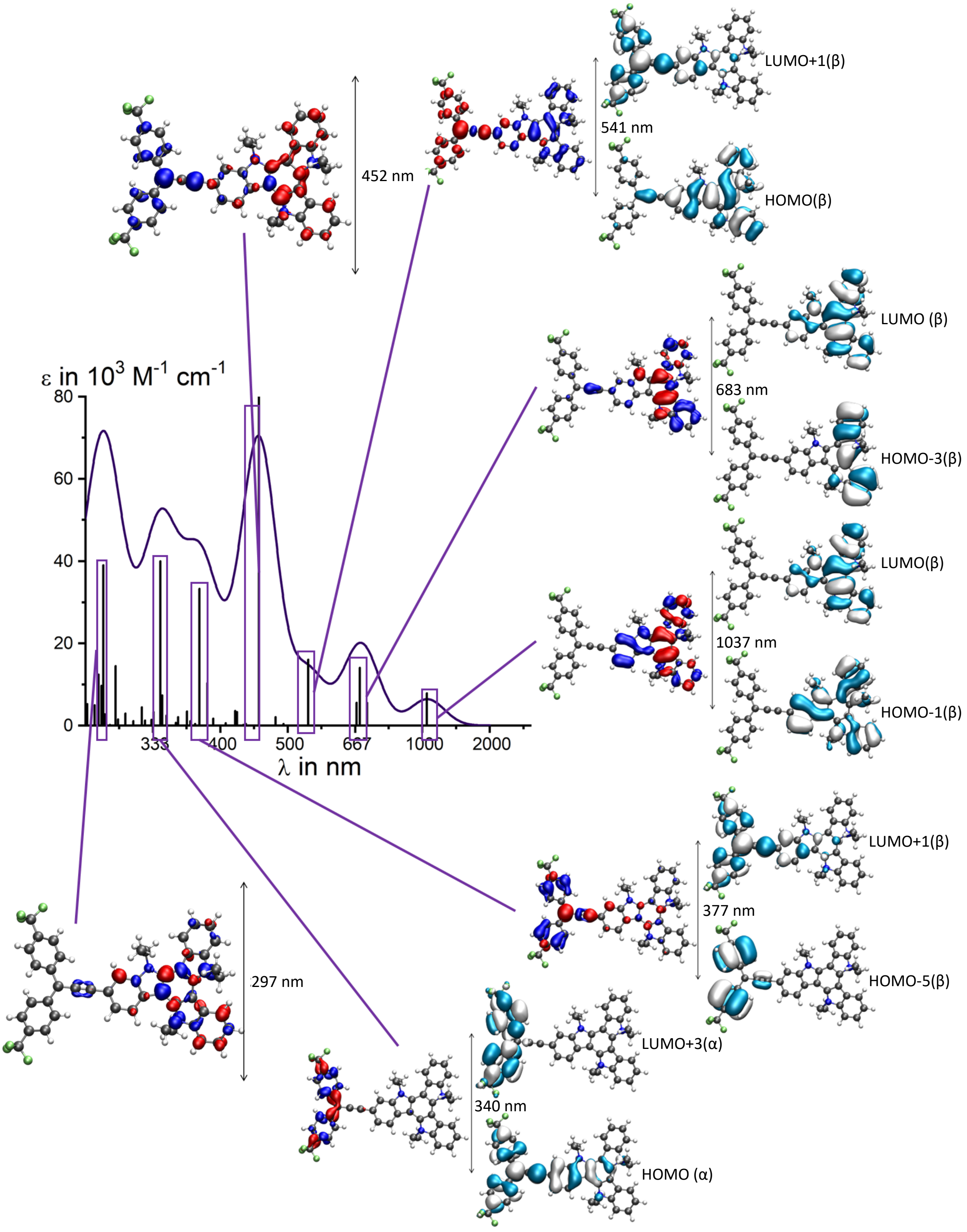

Figure S48. TD-DFT calculated UV/vis/NIR spectrum of the valence tautomer **1**$^{+••}$ with corresponding electron density difference maps (EDDMs; blue color: loss of electron density, red color: gain of electron density) for individual electronic transitions. Contributing MOs and band assignments are also provided.

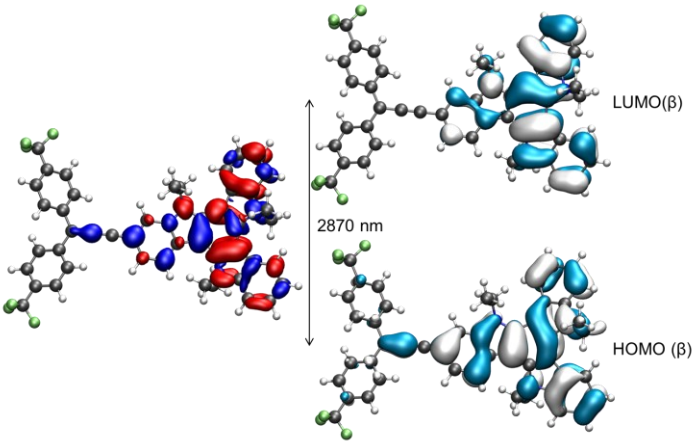

Figure S49. Molecular orbitals involved in the HOMO(β) to LUMO(β) transition of **1**$^{+\bullet\bullet}$ with the associated electron density difference map (blue color: loss of electron density, red color: gain of electron density).

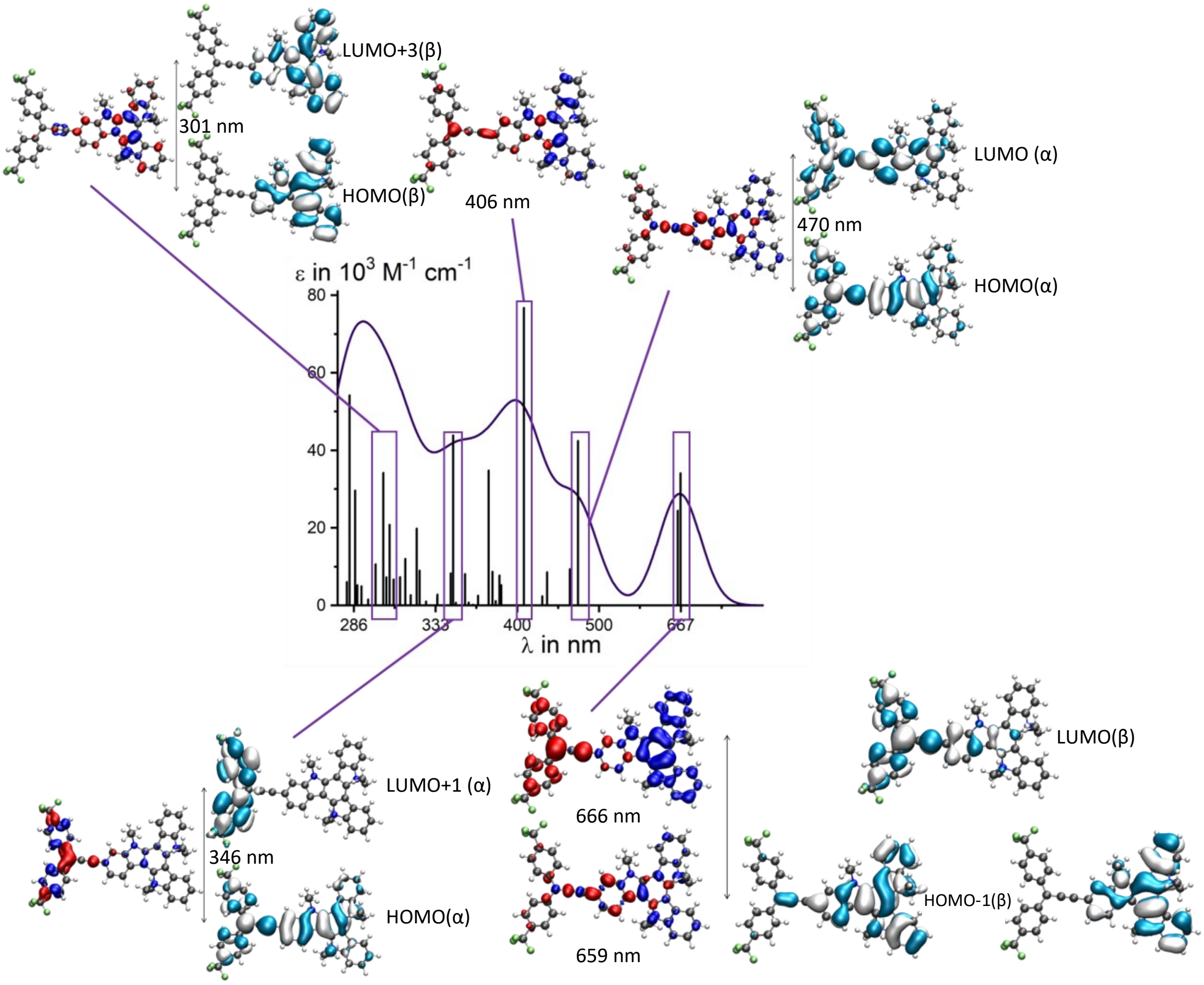

Figure S50. TD-DFT calculated UV/vis/NIR spectrum of **1**$^{\bullet}$ with corresponding electron density difference maps (EDDMs; blue color: loss of electron density, red color: gain of electron density) for individual electronic transitions. Contributing MOs and band assignments are also provided

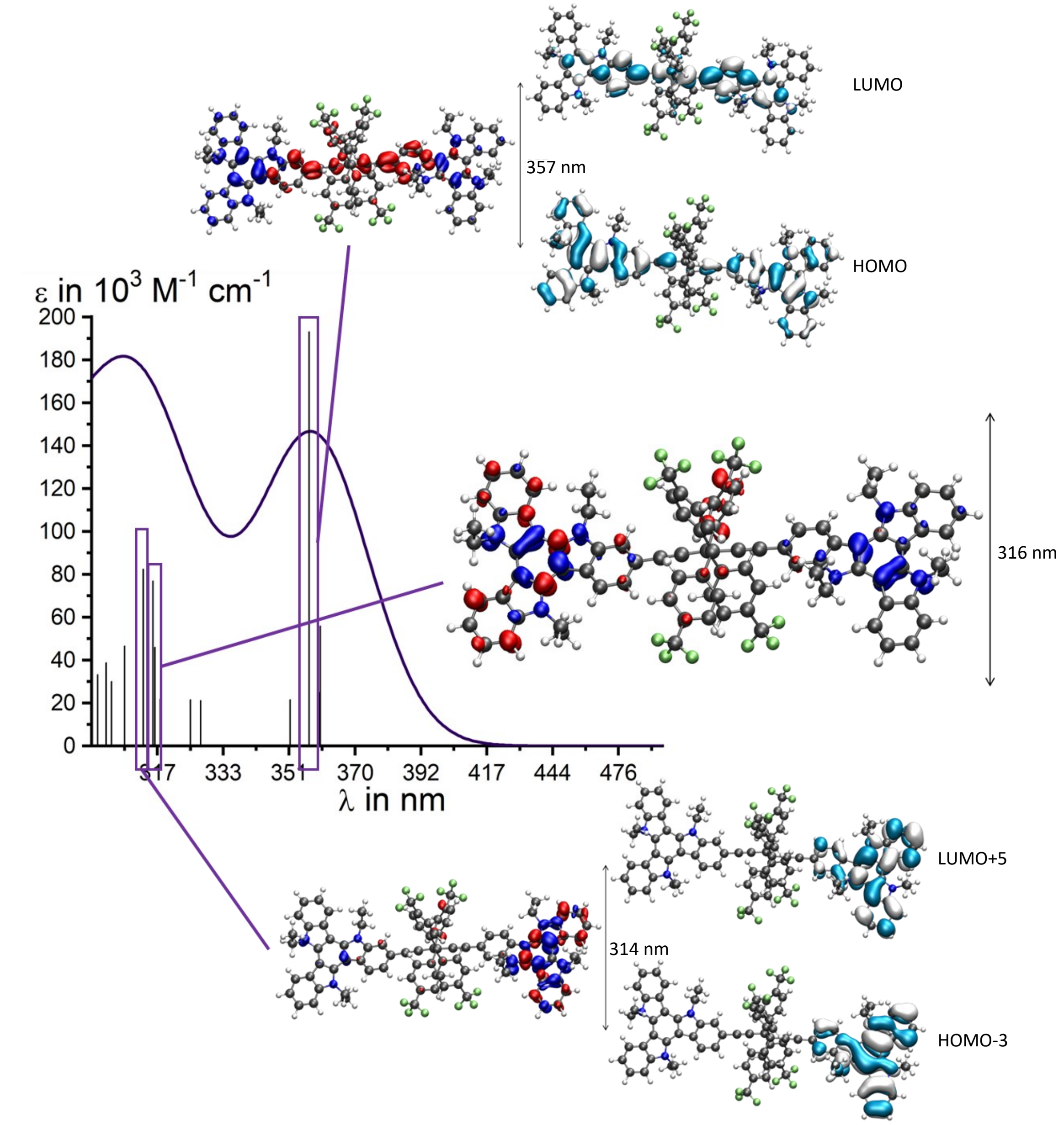

Figure S51. TD-DFT calculated UV/vis/NIR spectrum of **1-1** with corresponding electron density difference maps (EDDMs; blue color: loss of electron density, red color: gain of electron density) for the individual electronic transitions. Contributing MOs and band assignments are also provided

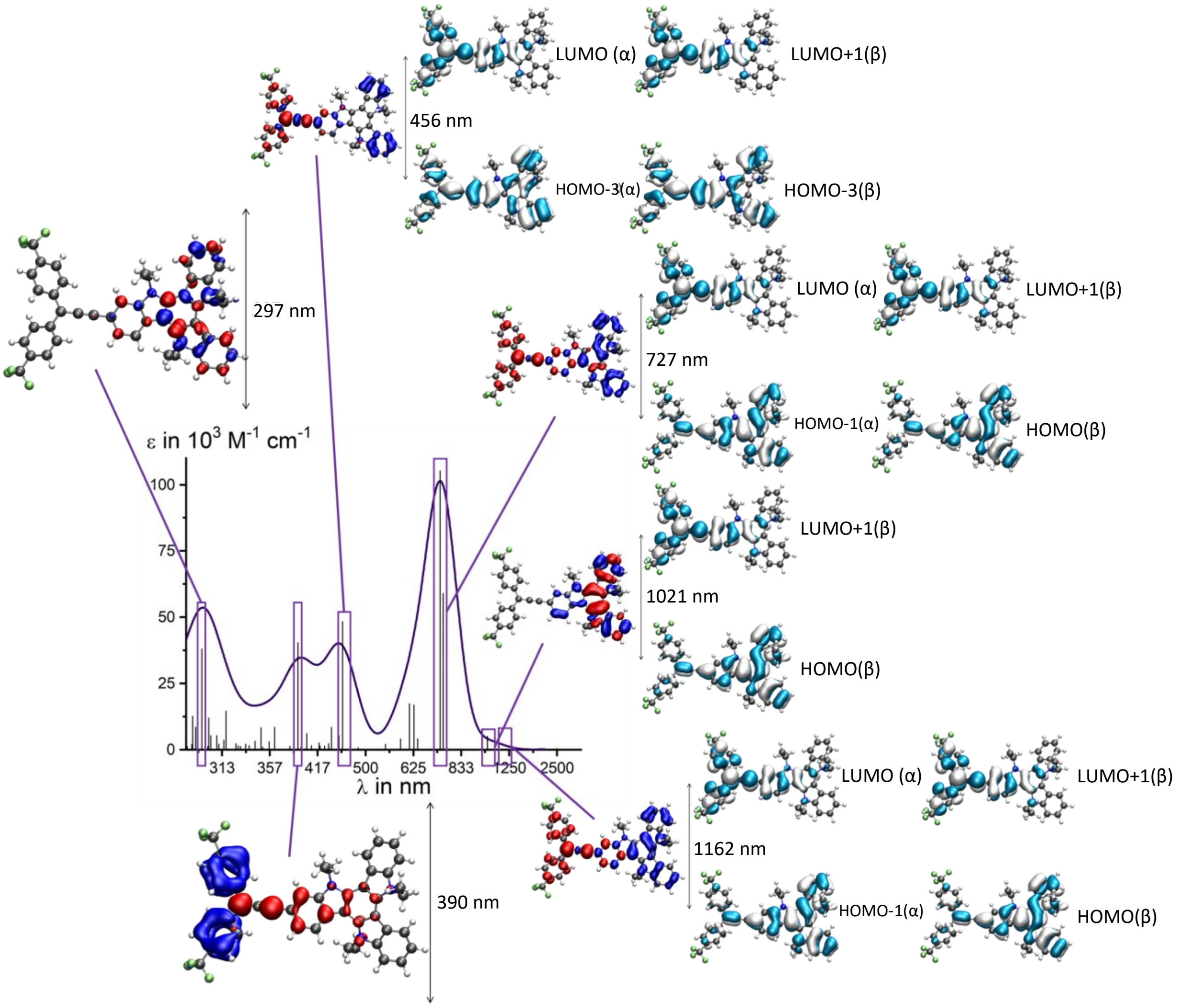

Figure S52. TD-DFT calculated UV/vis/NIR spectrum of the one-electron oxidized tritylium cation $\mathbf{1}^{2+\bullet}$ with EDDMs (EDDMs; blue color: loss of electron density, red color: gain of electron density) for the individual transitions. Contributing MOs and band assignments are also provided.

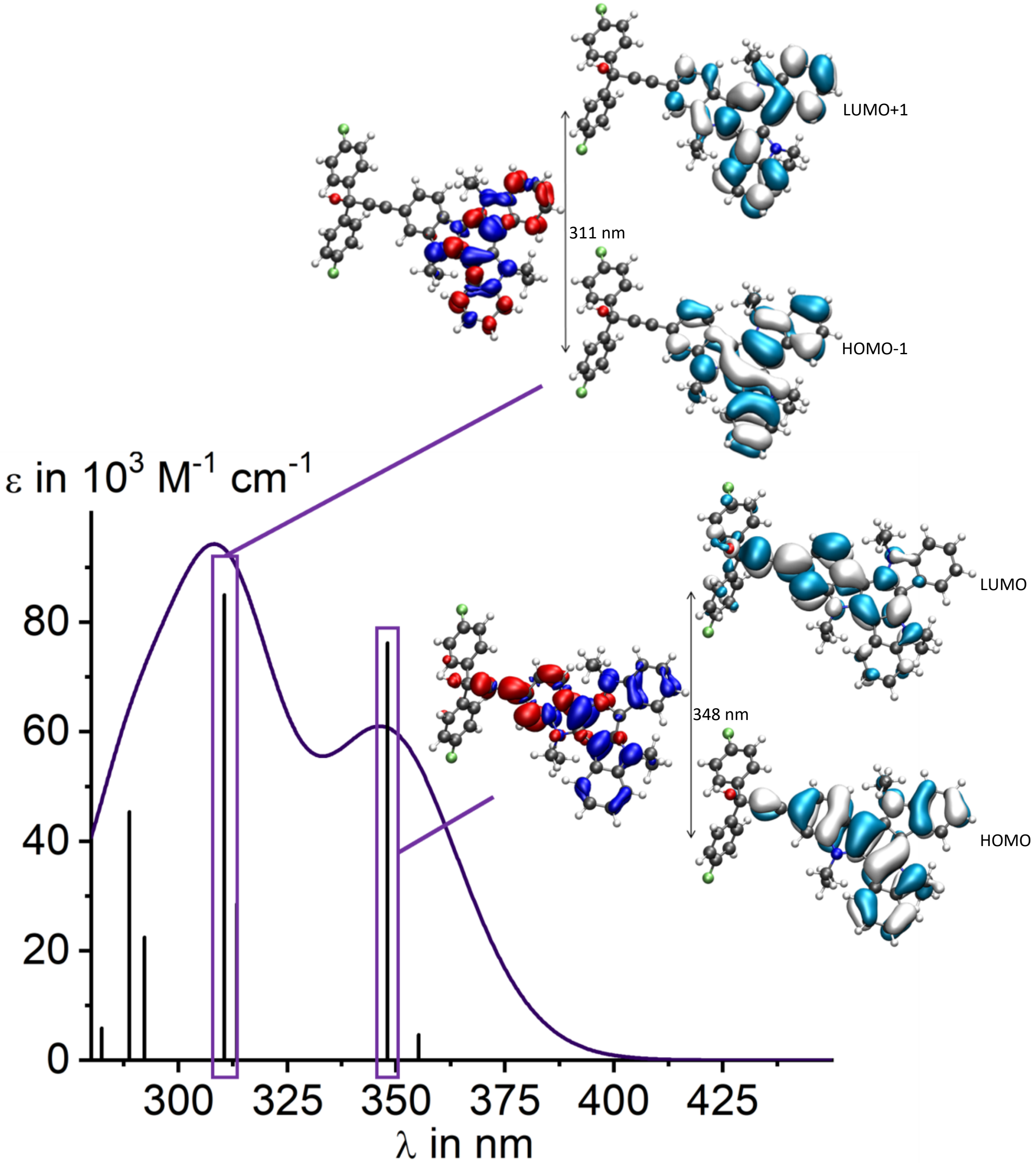

Figure S53. TD-DFT calculated UV/vis/NIR spectrum of **2-OH** with corresponding electron density difference maps (EDDMs; blue color: loss of electron density, red color: gain of electron density) for individual electronic transitions. Contributing MOs and band assignments are also provided.

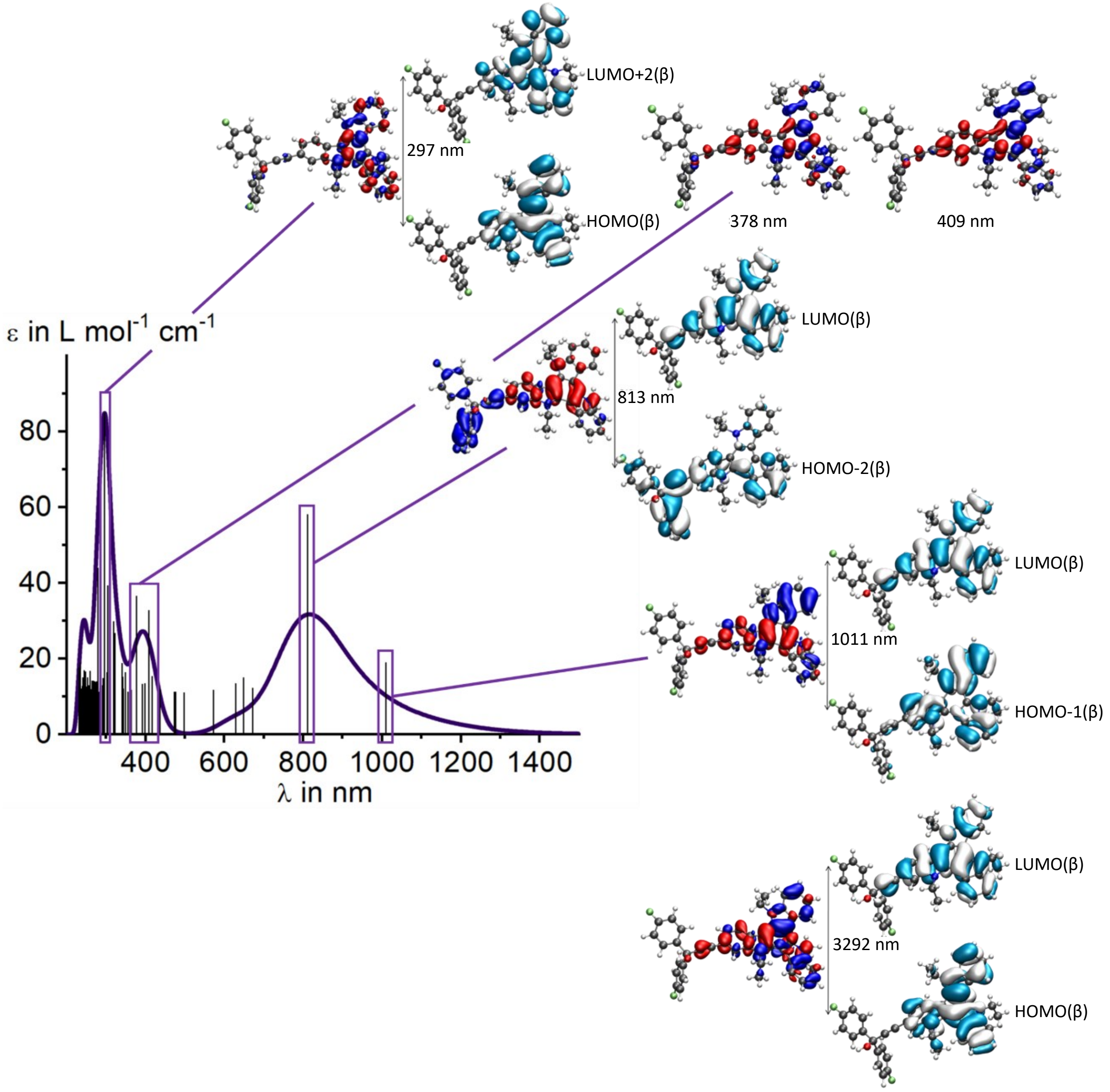

Figure S54. TD-DFT calculated UV/vis/NIR spectrum of **2-OH$^{+•}$** with corresponding electron density difference maps (EDDMs; blue color: loss of electron density, red color: gain of electron density) for individual electronic transitions. Contributing MOs and band assignments are also provided.

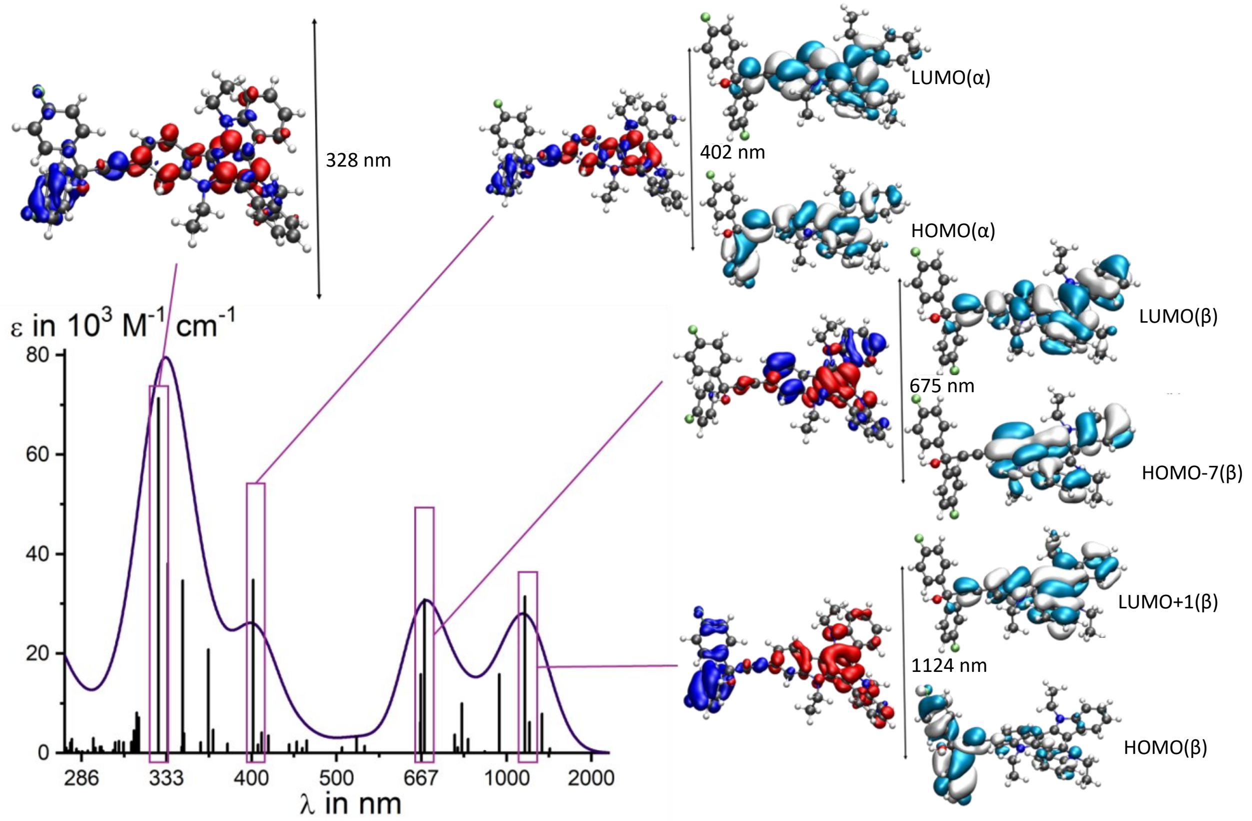

Figure S55. TD-DFT calculated UV/vis/NIR spectrum of **2-OH$^{2+••}$** with corresponding electron density difference maps (EDDMs; blue color: loss of electron density, red color: gain of electron density) for individual electronic transitions. Contributing MOs and band assignments are also provided.

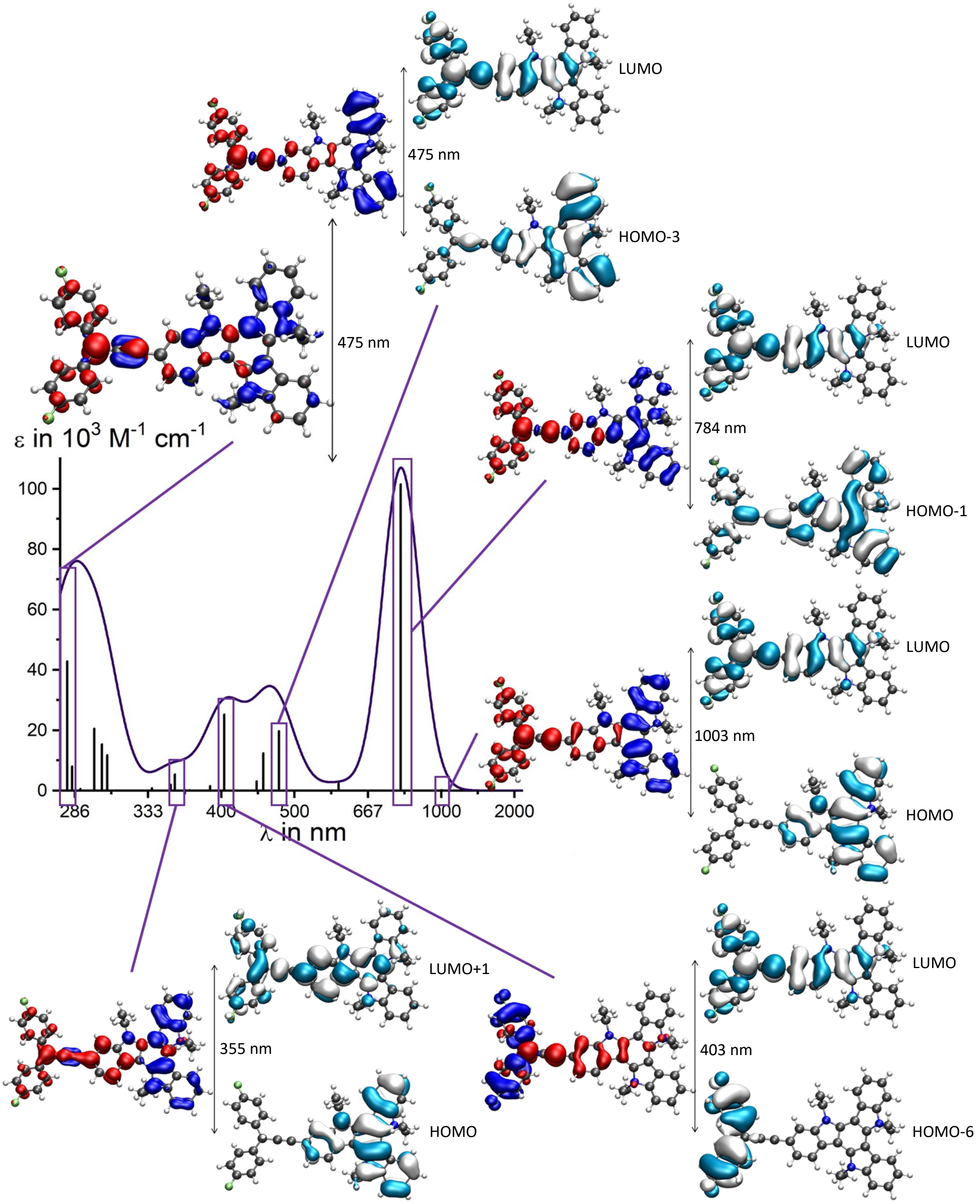

Figure S56. TD-DFT calculated UV/vis/NIR spectrum of the diamagnetic form of **2**$^{+}$ with corresponding electron density difference maps (EDDMs; blue color: loss of electron density, red color: gain of electron density) for individual electronic transitions. Contributing MOs and band assignments are also provided.

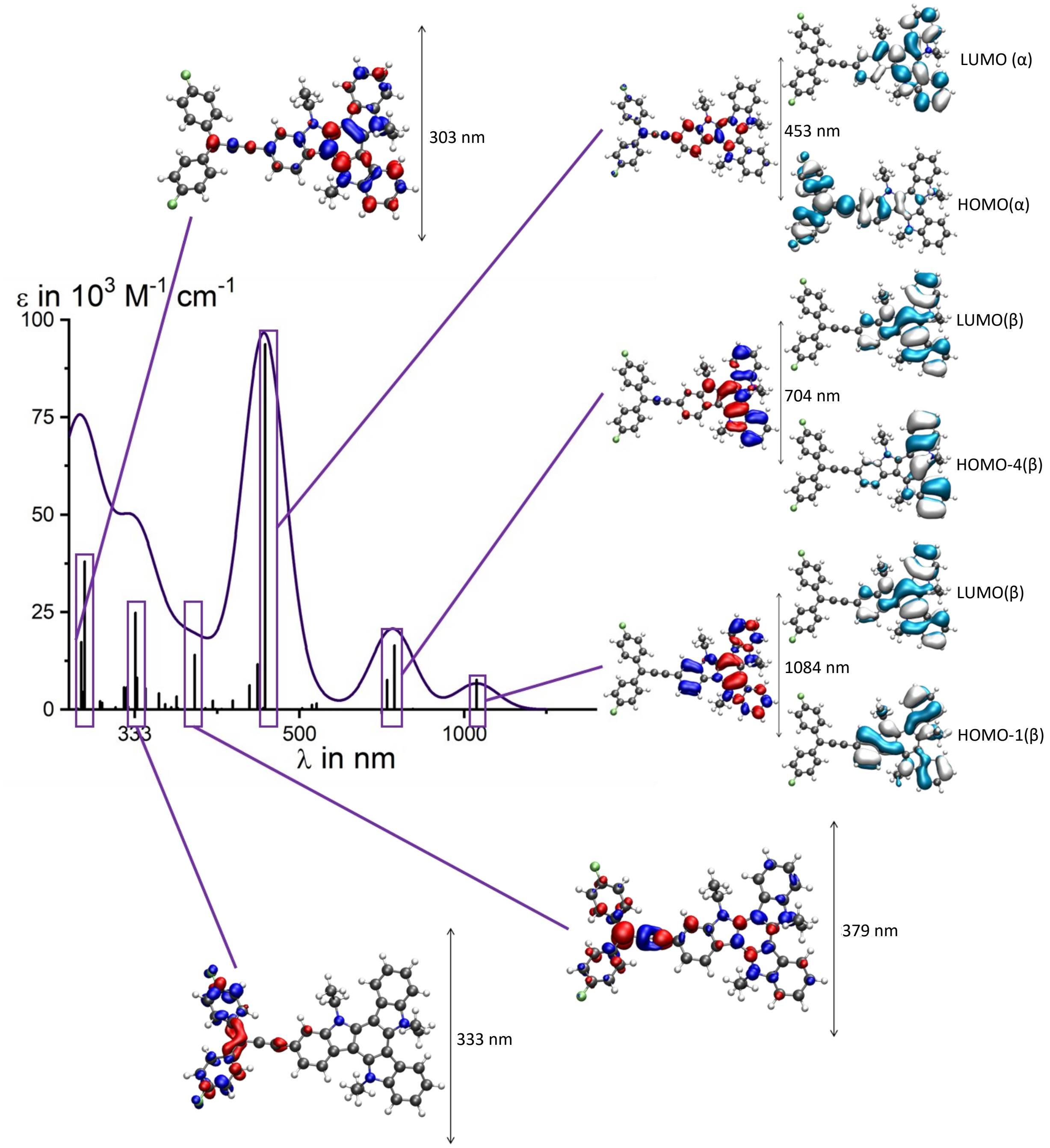

Figure S57. TD-DFT calculated UV/vis/NIR spectrum of $\mathbf{2}^{+\bullet\bullet}$, the paramagnetic valence tautomer of $\mathbf{2}^{+}$, with corresponding electron density difference maps (EDDMs; blue color: loss of electron density, red color: gain of electron density) for individual electronic transitions. Contributing MOs and band assignments are also provided.

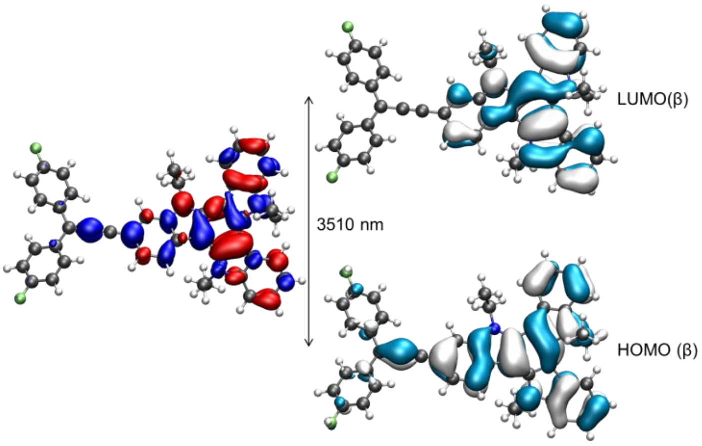

Figure S58. Molecular orbitals involved in the HOMO(β) to LUMO(β) transition of **2**$^{+••}$ with the associated electron density difference map (blue color: loss of electron density, red color: gain of electron density).

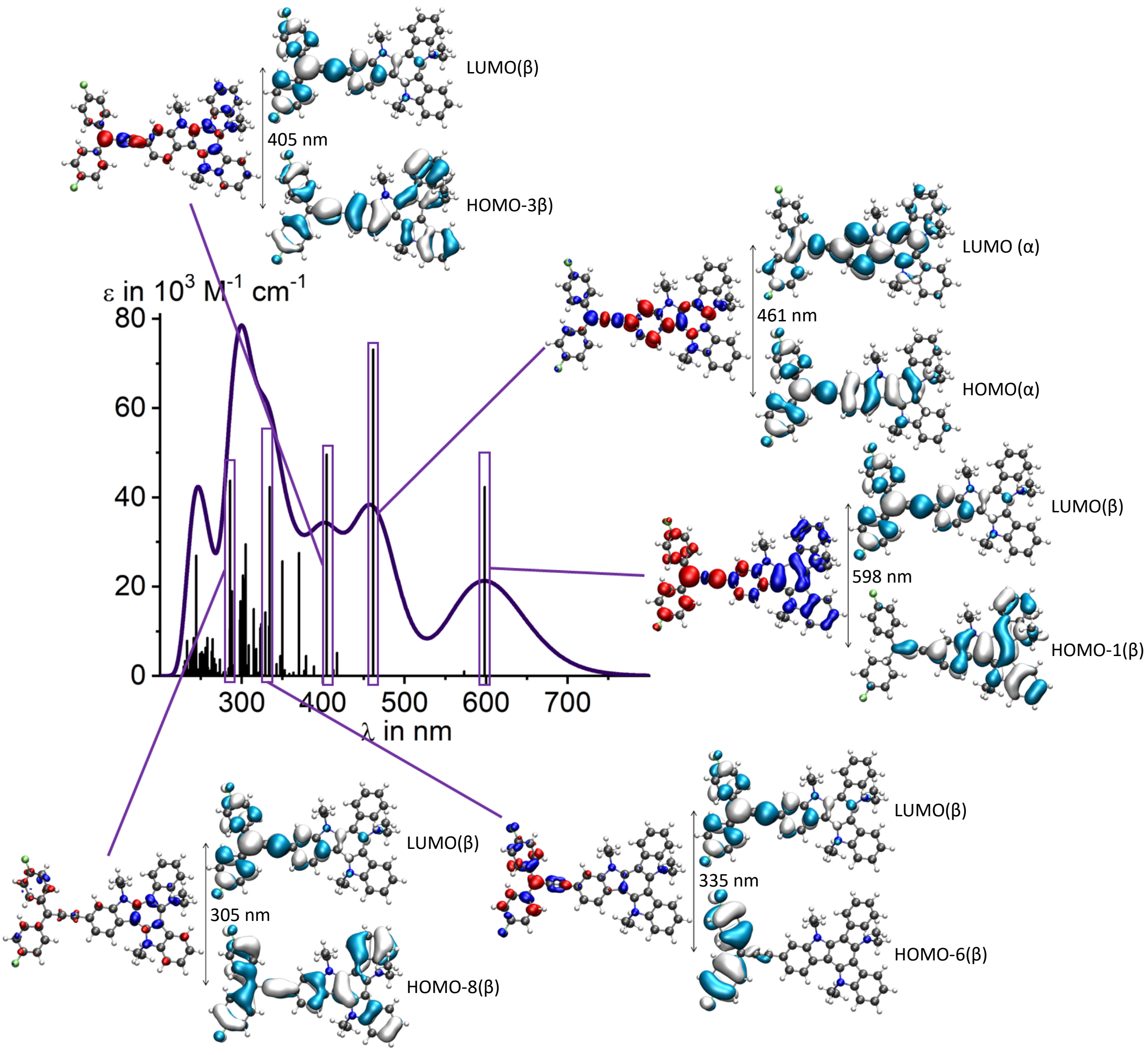

Figure S59. TD-DFT calculated UV/vis/NIR spectrum of **2**$^{•}$ with corresponding electron density difference maps (EDDMs; blue color: loss of electron density, red color: gain of electron density) for individual electronic transitions. Contributing MOs and band assignments are also provided.

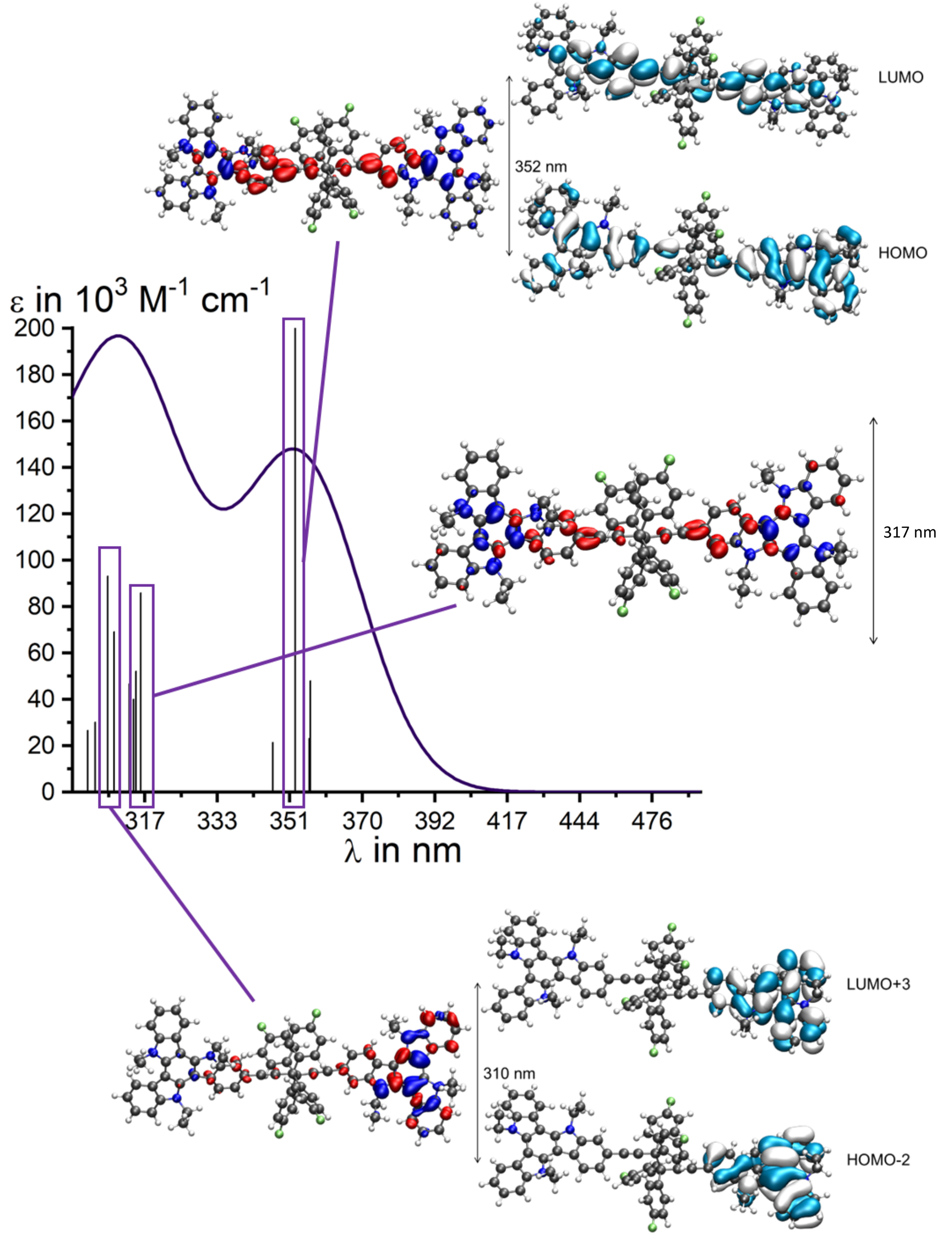

Figure S60. TD-DFT calculated UV/vis/NIR spectrum of neutral dimer **2-2** with corresponding electron density difference maps (EDDMs; blue color: loss of electron density, red color: gain of electron density) for individual electronic transitions. Contributing MOs and band assignments are also provided.

Computational data of the neutral form **^{Et}TAT**:

Sum of electronic and thermal Free Energies in Hartree/particle = –1321.795405

Imaginary frequencies: none

Coodinates (Z-matrix):

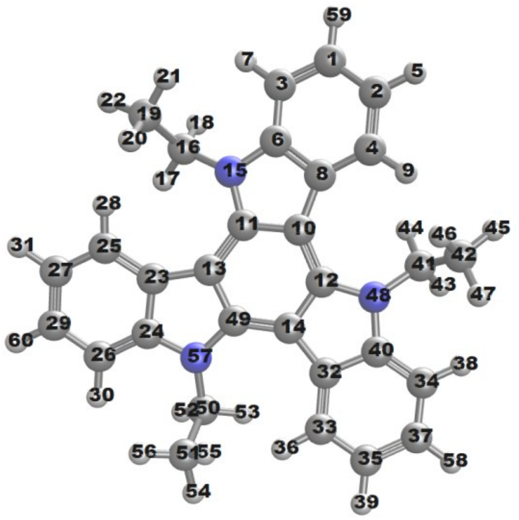

C

C 1 1.39890

C 1 1.38723 2 120.19243

C 2 1.38898 1 121.11202 3 2.38125

H 2 1.08644 1 119.81764 3 182.68511

C 3 1.39625 1 118.19620 2 359.10285

H 3 1.08518 1 120.45114 2 178.11201

C 4 1.40480 2 120.41291 1 0.25379

H 4 1.07974 2 117.81681 1 181.98470

C 8 1.45570 4 137.15021 2 179.64944

C 10 1.42316 8 106.51184 4 171.76243

C 10 1.41999 8 135.91782 4 350.54230

C 11 1.41990 10 122.24068 8 185.27834

C 12 1.42314 10 122.22163 8 175.10137

N 6 1.37764 3 127.41261 1 176.98594

C 15 1.45133 6 120.81665 3 10.70197

H 16 1.08922 15 108.04983 6 153.02313

H 16 1.09632 15 107.32044 6 39.29377

C 16 1.52456 15 113.38796 6 277.87135

H 19 1.09440 16 111.52834 15 303.90707

H 19 1.09381 16 111.19563 15 63.63714

H 19 1.09522 16 110.12427 15 183.26756

C 13 1.45555 11 135.90310 10 175.04293

C 23 1.41581 13 105.87942 11 173.97969

C 23 1.40471 13 137.12485 11 350.45739

C 24 1.39621 23 122.94028 13 183.08289

C 25 1.38897 23 120.40727 13 179.65920

H 25 1.07976 23 121.73340 13 357.86288

C 26 1.38731 24 118.19692 23 356.72353

H 26 1.08517 24 121.34548 23 177.72736

H 27 1.08644 25 119.07443 23 179.95554

C 14 1.45545 12 106.52595 10 185.35906

C 32 1.40471 14 137.11858 12 171.64506

C 32 2.47064 14 134.13999 12 353.23494

C 33 1.38899 32 120.40167 14 179.75231

H 33 1.07977 32 121.72579 14 357.91689

C 34 1.38728 32 89.48662 14 181.15566

H 34 1.08522 32 150.05629 14 0.16954

H 35 1.08644 33 119.07850 32 179.93485

C 34 1.39625 32 28.74179 14 4.02339

C 40 2.46020 34 97.38733 32 185.45568

C 41 1.52458 40 106.69063 34 296.59886

H 41 1.09628 40 84.58713 34 45.68040

H 41 1.08922 40 132.77630 34 152.27470

H 42 1.09524 41 110.14442 40 153.34561

H 42 1.09442 41 111.51581 40 273.98617

H 42 1.09383 41 111.16728 40 33.71019

N 40 1.37763 34 127.43723 32 180.16846

C 14 1.41980 12 117.59072 10 6.26881

C 24 2.46051 23 139.28965 13 354.41828

C 50 1.52463 24 106.70242 23 124.26674

H 50 1.09632 24 84.61313 23 233.34525

H 50 1.08925 24 132.78818 23 339.98531

H 51 1.09523 50 110.12369 24 153.58890

H 51 1.09443 50 111.52257 24 274.22389

H 51 1.09381 50 111.21224 24 33.95919

N 24 1.37772 23 109.62409 13 2.88814

H 37 1.08636 34 119.64142 32 178.97718

H 1 1.08636 2 120.14406 3 178.55756

H 29 1.08637 26 119.64038 24 180.53925

Computational data of the monocation of **^{Et}TAT** (**$^{Et}TAT^{+\bullet}$**):

Sum of electronic and thermal Free Energies in Hartree/particle = – 1321.614387

Imaginary frequencies: none

Coordinates (Z-matrix):

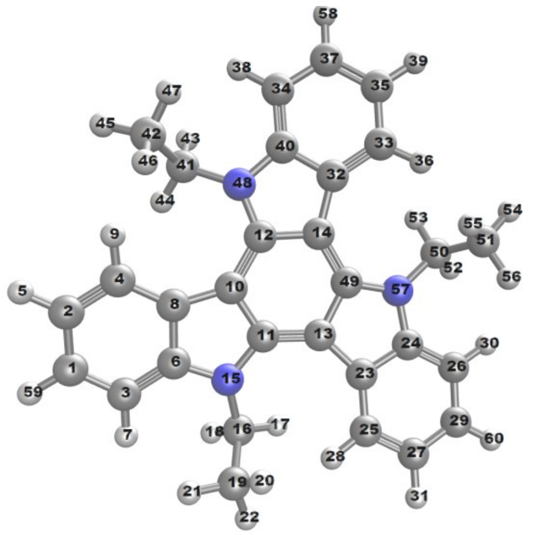

C

C 1 1.40990

C 1 1.38079 2 120.88466

C 2 1.37779 1 121.20865 3 2.96597

H 2 1.08539 1 119.47286 3 183.45581

C 3 1.39933 1 117.79484 2 358.96684

H 3 1.08395 1 120.67318 2 177.83176

C 4 1.41442 2 119.89867 1 0.17568

H 4 1.07899 2 118.37650 1 182.63168

C 8 1.43129 4 136.73426 2 178.55921

C 10 1.43440 8 106.48788 4 170.76297

C 10 1.43515 8 135.87782 4 349.97778

C 11 1.39995 10 121.62853 8 188.50875

C 12 1.42434 10 122.45581 8 172.52479

N 6 1.36665 3 127.44281 1 176.60488

C 15 1.45748 6 121.30470 3 9.27656

H 16 1.08923 15 107.86407 6 154.53856

H 16 1.09518 15 107.00993 6 41.13043

C 16 1.52356 15 113.27127 6 279.52866

H 19 1.09423 16 111.81628 15 305.40043

H 19 1.09340 16 111.40962 15 65.33420

H 19 1.09456 16 109.72623 15 184.73690

C 13 1.44969 11 135.77850 10 174.31400

C 23 1.41050 13 105.94219 11 170.65373

C 23 1.40210 13 136.29346 11 347.42242

C 24 1.38610 23 123.02618 13 182.68422

C 25 1.39072 23 119.81874 13 179.69144

H 25 1.07926 23 121.80834 13 357.41279

C 27 1.39465 25 120.89213 23 0.24535

H 26 1.08432 24 121.93811 23 178.09094

H 27 1.08524 25 119.19862 23 179.84869

C 14 1.45711 12 106.55466 10 183.87238

C 32 1.40152 14 137.21854 12 170.82588

C 32 2.47244 14 133.65775 12 353.34354

C 33 1.38962 32 119.87329 14 180.32525

H 33 1.07973 32 122.09544 14 358.36359

C 34 1.38575 32 89.25462 14 180.63331

H 34 1.08445 32 150.18973 14 359.50400

H 35 1.08580 33 118.89653 32 180.08829

C 34 1.39544 32 28.48712 14 3.29479

C 40 2.46832 34 96.90647 32 182.96080

C 41 1.52423 40 105.20079 34 295.58051

H 41 1.09431 40 84.63046 34 45.16355

H 41 1.08874 40 133.31218 34 152.25047

H 42 1.09443 41 109.52443 40 154.86373

H 42 1.09414 41 111.82300 40 275.41001

H 42 1.09310 41 111.31909 40 35.49656

N 12 1.36484 10 128.87562 8 350.56989

C 14 1.40823 12 117.30861 10 7.71988

C 24 2.47702 23 139.80341 13 358.69174

C 50 1.52367 24 105.57849 23 118.04925

H 50 1.09462 24 84.38275 23 227.64304

H 50 1.08851 24 133.09905 23 334.16565

H 51 1.09446 50 109.64066 24 152.96452

H 51 1.09419 50 111.68509 24 273.45845

H 51 1.09347 50 111.31212 24 33.60599

N 49 1.35709 14 130.16984 12 171.07648

H 37 1.08545 34 119.68230 32 178.95372

H 1 1.08572 2 119.60157 3 178.26271

H 29 1.08560 27 120.02281 25 180.79296

Computational data of the neutral carbinol **1-OH**:

Sum of electronic and thermal Free Energies in Hartree/particle = –2647.096973

Imaginary frequencies: none

Coordinates (Z-matrix):

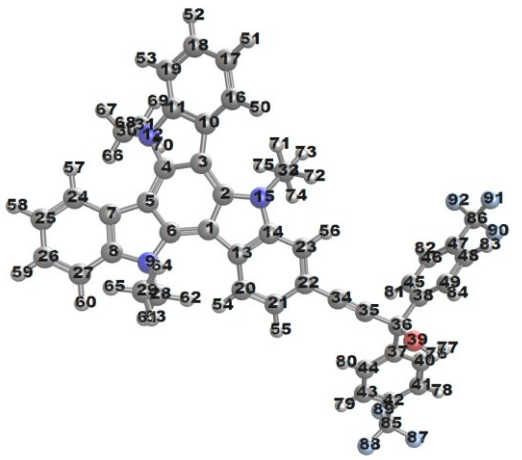

C

C 1 1.41979

C 2 1.41806 1 121.48111

C 3 1.41741 2 116.73125 1 349.13060

C 4 1.41614 3 121.86497 2 18.65198

C 5 1.41240 4 117.53443 3 349.78593

C 5 1.45002 4 135.17300 3 158.96411

C 7 1.41645 5 105.86164 4 188.40319

N 6 1.38412 5 109.68687 4 173.39352

C 3 1.45176 2 137.12477 1 173.68252

C 10 1.41799 3 106.04218 2 168.67745

N 4 1.38369 3 109.53572 2 191.58873

C 1 1.44162 2 106.85274 3 171.74768

C 13 1.41744 1 106.04658 2 2.96663

N 2 1.38589 1 109.03144 3 184.72108

C 10 1.40297 3 135.93305 2 341.53236

C 16 1.39004 10 119.82887 3 182.32002

C 17 1.39988 16 121.04702 10 1.05877

C 18 1.38957 17 120.55838 16 2.46871

C 13 1.40417 1 135.61911 2 189.83472

C 20 1.38435 13 120.00631 1 176.52464

C 21 1.41130 20 121.12522 13 358.91625

C 14 1.38954 13 122.44927 1 181.31745

C 7 1.40317 5 135.94885 4 2.66544

C 24 1.38947 7 119.74343 5 183.04376

C 25 1.40025 24 121.06532 7 0.86982

C 26 1.38896 25 120.58932 24 1.59338

C 9 1.45985 6 124.56113 5 210.11658

C 28 1.52404 9 112.97262 6 83.58542

C 12 1.45876 4 124.56594 3 147.54860

C 30 1.52415 12 112.85839 4 266.91693

C 15 1.45704 2 125.67288 1 154.46881

C 32 1.52302 15 112.84489 2 283.26259

C 22 1.42504 21 120.17320 20 179.39301

C 2 7.11419 1 73.79797 3 188.53648

C 2 8.53504 1 76.49562 3 188.53571

C 36 1.53506 35 112.05309 34 233.41500

C 36 1.53721 35 107.18339 34 355.92371

O 36 1.42630 35 106.42228 34 115.00959

C 37 1.39860 36 119.17096 35 163.98588

C 40 1.38923 37 120.64707 36 175.06101

C 41 1.39353 40 119.74909 37 0.27109

C 42 1.39292 41 120.08071 40 359.79870

C 43 1.39016 42 119.89885 41 359.91300

C 38 1.39823 36 120.23351 35 288.12557

C 45 1.38728 38 120.43661 36 178.46572

C 46 1.39607 45 119.80232 38 359.87847

C 47 1.39128 46 120.09158 45 359.78910

C 38 1.39236 36 120.31556 35 105.98578

H 16 1.08470 10 121.38896 3 358.85291

H 17 1.08633 16 119.26402 10 179.90878

H 18 1.08635 17 119.88157 16 180.32277

H 19 1.08606 18 120.84604 17 176.59353

H 20 1.08431 13 121.30305 1 359.46711

H 21 1.08551 20 119.82963 13 179.89182

H 23 1.08481 14 121.91769 13 180.35508

H 24 1.08375 7 121.44124 5 0.77686

H 25 1.08640 24 119.26994 7 180.15897

H 26 1.08637 25 119.88268 24 180.29689

H 27 1.08601 26 120.71791 25 177.86816

H 28 1.09494 9 108.17756 6 206.97896

H 28 1.08672 9 108.33318 6 322.59832

H 29 1.09496 28 109.65029 9 180.87465

H 29 1.09481 28 110.49606 9 300.05823

H 29 1.09389 28 111.71937 9 60.86726

H 30 1.08808 12 107.63366 4 27.00511

H 30 1.09615 12 108.89592 4 143.22006

H 31 1.09491 30 109.55363 12 178.70273

H 31 1.09370 30 111.90944 12 298.66044

H 31 1.09478 30 110.48111 12 59.50357

H 32 1.08522 15 108.79762 2 45.43816

H 32 1.09370 15 107.57608 2 160.39423

H 33 1.09486 32 109.84150 15 180.50020

H 33 1.09450 32 111.33064 15 300.42879

H 33 1.09477 32 110.60821 15 61.13391

H 39 0.97012 36 107.06712 35 168.50821

H 40 1.08692 37 120.12443 36 354.58047

H 41 1.08533 40 120.03915 37 180.23079

H 43 1.08543 42 120.12109 41 180.28060

H 44 1.08567 43 119.84806 42 180.15278

H 45 1.08663 38 119.91418 36 358.60113

H 46 1.08558 45 120.07571 38 180.21580

H 48 1.08539 47 120.12702 46 180.61533

H 49 1.08471 38 119.36095 36 0.88736

C 42 1.49956 41 119.90614 40 177.16701

C 47 1.49871 46 119.64793 45 177.43733

F 85 1.34336 42 111.94157 41 30.99685

F 85 1.34319 42 111.95280 41 151.72595

F 85 1.34690 42 111.67672 41 271.37011

F 86 1.34760 47 111.73101 46 275.32397

F 86 1.34312 47 112.11957 46 155.52794

F 86 1.34417 47 111.89045 46 34.76900

Computational data of the monocation of carbinol **1-OH** (**1-OH**$^{+\bullet}$):

Sum of electronic and thermal Free Energies in Hartree/particle = –2646.910745

Imaginary frequencies: none

Coordinates (Z-matrix):

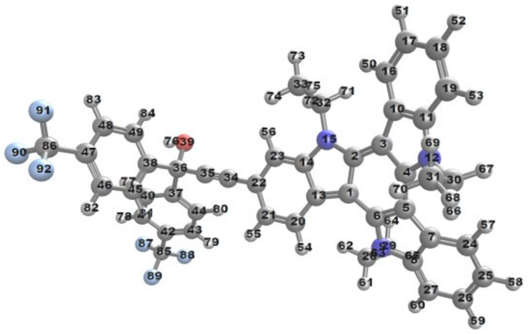

C

C 1 1.45694

C 2 1.39995 1 120.66047

C 3 1.44724 2 116.07227 1 358.63351

C 4 1.42237 3 122.88117 2 17.24575

C 5 1.40377 4 117.07016 3 344.57206

C 5 1.44027 4 135.48068 3 155.31942

C 7 1.42003 5 105.52000 4 186.61913

N 6 1.37567 5 109.70280 4 174.74465

C 3 1.46403 2 137.54074 1 191.12637

C 10 1.41201 3 105.88503 2 164.25726

N 4 1.34850 3 109.31556 2 195.20266

C 1 1.41711 2 106.96072 3 165.01929

C 13 1.42234 1 106.20385 2 6.78762

N 2 1.37547 1 107.40518 3 186.44330

C 10 1.39864 3 137.00820 2 342.41063

C 16 1.39397 10 119.93419 3 181.29175

C 17 1.39351 16 121.38074 10 0.27844

C 11 1.38673 10 123.72898 3 179.29262

C 13 1.41214 1 134.88433 2 192.71927

C 20 1.37597 13 119.48467 1 178.27645

C 21 1.41952 20 120.89026 13 359.33071

C 14 1.38566 13 122.19612 1 178.88855

C 7 1.40588 5 136.11571 4 0.53718

C 24 1.38437 7 119.28477 5 183.12155

C 25 1.40669 24 121.39688 7 0.68296

C 26 1.38294 25 120.83766 24 2.04107

C 9 1.46275 6 126.73324 5 203.38679

C 28 1.52331 9 112.46288 6 88.64453

C 12 1.46159 4 126.32853 3 156.68557

C 30 1.52406 12 111.99003 4 259.67423

C 15 1.45394 2 128.96851 1 185.05647

C 32 1.52111 15 113.41749 2 268.45123

C 22 1.41818 21 119.63368 20 179.15073

C 2 7.09790 1 72.81507 3 193.37965

C 2 8.52561 1 75.36048 3 193.57772

C 36 1.53338 35 111.97499 34 227.80229

C 36 1.53784 35 106.47924 34 350.32369

O 36 1.42253 35 105.95211 34 109.12850

C 37 1.39818 36 119.17688 35 162.24156

C 40 1.38938 37 120.53407 36 174.85354

C 41 1.39333 40 119.77284 37 0.25602

C 42 1.39285 41 120.13158 40 359.71804

C 43 1.38995 42 119.86549 41 359.96789

C 38 1.39813 36 120.31961 35 287.58925

C 45 1.38742 38 120.33243 36 178.54493

C 46 1.39575 45 119.80237 38 359.88932

C 47 1.39144 46 120.14552 45 359.74186

C 48 1.39204 47 119.87870 46 0.03723

H 16 1.07970 10 122.29283 3 0.26365

H 17 1.08557 16 118.84714 10 179.74529

H 18 1.08543 17 120.16948 16 179.57361

H 19 1.08501 11 121.44711 10 180.75731

H 20 1.08423 13 121.15115 1 1.47763

H 21 1.08444 20 120.19713 13 180.51665

H 23 1.08375 14 122.40381 13 180.88843

H 24 1.08355 7 121.60867 5 0.78020

H 25 1.08577 24 119.24002 7 180.03139

H 26 1.08567 25 119.61811 24 180.69871

H 27 1.08517 26 120.90819 25 177.93802

H 28 1.09394 9 107.79603 6 211.95587

H 28 1.08554 9 108.44927 6 327.55910

H 29 1.09432 28 109.17269 9 180.58498

H 29 1.09442 28 110.61654 9 299.61421

H 29 1.09338 28 111.93365 9 60.78457

H 30 1.08653 12 107.64193 4 19.38451

H 30 1.09482 12 108.52651 4 136.06814

H 31 1.09412 30 108.71801 12 178.59760

H 31 1.09265 30 112.43512 12 298.34641

H 31 1.09402 30 110.50263 12 59.84414

H 32 1.08975 15 107.44492 2 34.07298

H 32 1.09366 15 106.74286 2 146.53476

H 33 1.09462 32 109.74764 15 179.80354

H 33 1.09429 32 110.80827 15 299.10030

H 33 1.09375 32 111.55625 15 59.68438

H 39 0.97013 36 107.05134 35 168.01840

H 40 1.08674 37 120.18620 36 354.41958

H 41 1.08524 40 120.01623 37 180.21871

H 43 1.08532 42 120.13834 41 180.36872

H 44 1.08581 43 119.69422 42 180.28905

H 45 1.08657 38 120.04131 36 358.48662

H 46 1.08550 45 120.06008 38 180.15580

H 48 1.08529 47 120.13926 46 180.54201

H 49 1.08465 48 120.30018 47 180.80620

C 42 1.50019 41 119.88926 40 177.11871

C 47 1.49949 46 119.62398 45 177.38732

F 85 1.34275 42 111.91093 41 31.10151

F 85 1.34290 42 111.91208 41 151.85614

F 85 1.34654 42 111.58510 41 271.47193

F 86 1.34685 47 111.66550 46 275.25605

F 86 1.34280 47 112.06648 46 155.44784

F 86 1.34384 47 111.83412 46 34.71405

Computational data of the cation **1⁺**:

Sum of electronic and thermal Free Energies in Hartree/particle = –2571.192521

Imaginary frequencies: none

Coordinates (Z-matrix):

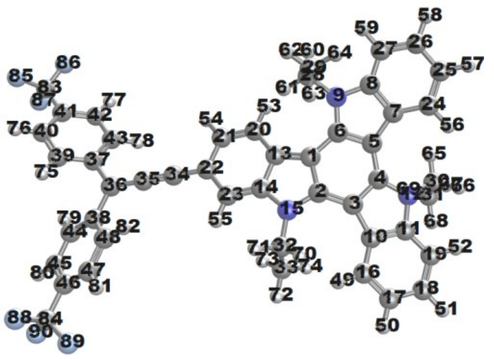

C

C 1 1.44852

C 2 1.41069 1 120.98008

C 3 1.43287 2 115.95528 1 358.27514

C 4 1.42114 3 123.07087 2 16.63819

C 5 1.40270 4 117.39153 3 345.04977

C 5 1.44623 4 135.27412 3 155.57980

C 7 1.41615 5 105.60384 4 186.73903

N 6 1.37581 5 109.91274 4 174.87246

C 3 1.46221 2 137.65814 1 189.79735

C 10 1.41522 3 105.85049 2 165.67775

N 4 1.36086 3 109.65863 2 193.38633

C 1 1.40556 2 107.24214 3 165.15099

C 13 1.43397 1 106.40364 2 6.90731

N 2 1.37539 1 107.61904 3 186.29777

C 10 1.40229 3 137.26060 2 343.95102

C 16 1.39027 10 120.17322 3 180.88539

C 17 1.39737 16 121.38457 10 0.28527

C 18 1.38820 17 120.15914 16 0.31907

C 13 1.41961 1 134.76720 2 192.96579

C 20 1.36738 13 119.70992 1 178.84047

C 21 1.43332 20 120.89630 13 359.23196

C 14 1.37056 13 122.40806 1 178.23853

C 7 1.40454 5 136.02350 4 0.46670

C 24 1.38700 7 119.47302 5 183.42603

C 25 1.40259 24 121.19902 7 0.81579

C 26 1.38654 25 120.73308 24 1.81769

C 9 1.46095 6 126.21428 5 205.91584

C 28 1.52331 9 112.62005 6 84.58298

C 12 1.46049 4 125.69511 3 154.21093

C 30 1.52393 12 112.41074 4 262.61419

C 15 1.45273 2 129.08180 1 184.96510

C 32 1.52143 15 113.40488 2 268.89016

C 22 1.37372 21 119.86635 20 178.77610

C 2 7.08855 1 72.35423 3 192.77572

C 2 8.41450 1 74.56671 3 192.60537

C 36 1.46521 35 119.04433 34 242.31546

C 36 1.46531 35 118.95293 34 62.22129

C 37 1.40591 36 120.84172 35 211.07068

C 39 1.38703 37 120.41067 36 177.97391

C 40 1.39355 39 119.87238 37 0.44469

C 41 1.39574 40 120.41154 39 359.28853

C 42 1.38446 41 119.78026 40 0.10064

C 38 1.40566 36 120.83881 35 211.48184

C 44 1.38724 38 120.41374 36 177.77927

C 45 1.39334 44 119.85559 38 0.42919

C 46 1.39599 45 120.42145 44 359.39041

C 47 1.38429 46 119.78456 45 0.00058

H 16 1.07989 10 122.18089 3 359.96211

H 17 1.08599 16 118.90779 10 179.75345

H 18 1.08582 17 120.11253 16 179.61197

H 19 1.08548 18 120.92356 17 178.78802

H 20 1.08392 13 120.83306 1 2.01558

H 21 1.08465 20 120.54700 13 180.42865

H 23 1.08426 14 122.84327 13 181.26077

H 24 1.08390 7 121.50071 5 1.08167

H 25 1.08606 24 119.25634 7 180.07618

H 26 1.08601 25 119.76122 24 180.41261

H 27 1.08554 26 120.76971 25 177.66121

H 28 1.09414 9 107.79976 6 207.73501

H 28 1.08619 9 108.71923 6 323.14526

H 29 1.09459 28 109.48585 9 180.17016

H 29 1.09463 28 110.60182 9 299.37371

H 29 1.09365 28 111.68461 9 60.28522

H 30 1.08695 12 107.66228 4 22.44844

H 30 1.09515 12 108.72035 4 138.84564

H 31 1.09447 30 109.06537 12 178.60189

H 31 1.09305 30 112.28328 12 298.47064

H 31 1.09442 30 110.47774 12 59.69853

H 32 1.08954 15 107.48798 2 34.34221

H 32 1.09391 15 106.80286 2 146.90337

H 33 1.09472 32 109.80897 15 179.71575

H 33 1.09439 32 110.81904 15 299.10859

H 33 1.09362 32 111.40904 15 59.69663

H 39 1.08484 37 120.06875 36 0.30127

H 40 1.08483 39 119.91553 37 181.20708

H 42 1.08492 41 120.16886 40 180.26324

H 43 1.08552 42 119.89908 41 179.51062

H 44 1.08484 38 120.04658 36 0.15771

H 45 1.08475 44 119.93987 38 181.24725

H 47 1.08504 46 120.15235 45 180.17947

H 48 1.08558 47 119.95327 46 179.55961

C 41 1.50299 40 119.84369 39 177.12799

C 46 1.50295 45 119.98552 44 177.29665

F 83 1.34099 41 111.80236 40 29.83315

F 83 1.34140 41 111.72809 40 150.59854

F 83 1.34448 41 111.21225 40 270.16329

F 84 1.34445 46 111.22799 45 267.86050

F 84 1.34176 46 111.64804 45 148.39570

F 84 1.34073 46 111.86963 45 27.64887

Computational data of the valence tautomer of cation **1**$^{+}$ (**1**$^{+\bullet\bullet}$):

Sum of electronic and thermal Free Energies in Hartree/particle = –2571.178369

Imaginary frequencies: none

Coordinates (Z-matrix):

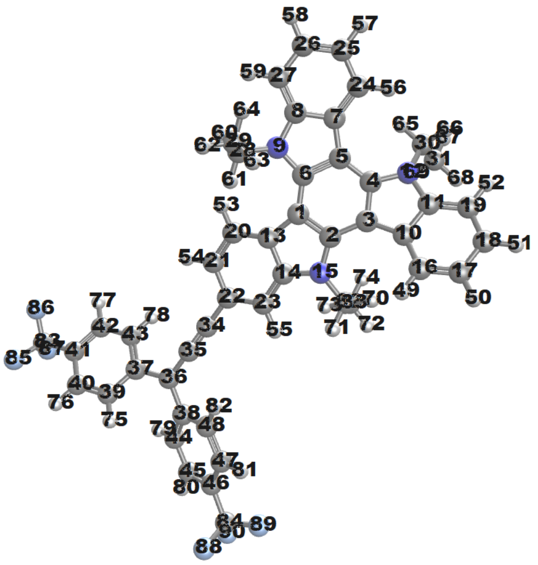

C

C 1 1.41676

C 2 1.43324 1 121.55903

C 3 1.43410 2 116.95175 1 345.62605

C 4 1.39470 3 120.68969 2 22.59021

C 1 1.41288 2 117.31790 3 356.07179

C 5 1.44767 4 135.28608 3 157.85963

C 7 1.41111 5 105.95333 4 187.95812

N 6 1.35146 1 129.81104 2 195.47588

C 3 1.42528 2 137.02744 1 167.99082

C 10 1.42905 3 106.15864 2 169.33349

N 11 1.37409 10 109.88556 3 4.23353

C 1 1.44088 2 106.90615 3 171.60563

C 13 1.41728 1 105.72102 2 2.65025

N 2 1.36662 1 109.19305 3 186.02832

C 10 1.41287 3 135.37443 2 343.58821

C 16 1.37854 10 119.25401 3 180.81975

C 17 1.41121 16 121.07757 10 0.86429

C 18 1.38417 17 121.38630 16 2.56068

C 13 1.40500 1 136.15230 2 189.47423

C 20 1.38129 13 119.74714 1 176.85685

C 21 1.41929 20 121.65084 13 358.95657

C 14 1.38864 13 123.15678 1 180.74578

C 7 1.39989 5 135.52456 4 2.73339

C 24 1.39252 7 119.28020 5 181.69043

C 25 1.39496 24 120.97805 7 0.95118

C 8 1.38455 7 122.84240 5 180.35074

C 9 1.46178 6 126.41771 1 21.95363

C 28 1.52402 9 112.23876 6 94.72869

C 12 1.46383 11 122.70059 10 204.35478

C 30 1.52295 12 112.58818 11 57.77909

C 15 1.46064 2 127.62734 1 161.74271

C 32 1.52314 15 112.25539 2 271.69754

C 22 1.40865 21 120.11698 20 179.32053

C 2 7.11232 1 74.02990 3 188.48769

C 2 8.46615 1 76.23204 3 188.44958

C 36 1.46325 35 118.43775 34 323.96840

C 36 1.46330 35 118.47424 34 143.94949

C 37 1.40828 36 121.70769 35 208.49886

C 39 1.38673 37 120.92812 36 178.31916

C 40 1.39471 39 120.10577 37 359.90270

C 41 1.39573 40 119.86932 39 359.43373

C 42 1.38498 41 119.99012 40 0.33575

C 38 1.40812 36 121.71303 35 208.89381

C 44 1.38687 38 120.93276 36 178.36631

C 45 1.39453 44 120.10236 38 359.91134

C 46 1.39590 45 119.86692 44 359.39445

C 47 1.38490 46 119.99402 45 0.36674

H 16 1.08395 10 121.29536 3 357.18035

H 17 1.08527 16 119.56971 10 179.50410

H 18 1.08575 17 119.29482 16 180.25267

H 19 1.08477 18 120.97336 17 176.42774

H 20 1.08354 13 121.71511 1 359.54791

H 21 1.08501 20 119.66719 13 179.81457

H 23 1.08425 14 122.07645 13 180.52807

H 24 1.08256 7 121.74985 5 359.83241

H 25 1.08528 24 119.26524 7 180.49770

H 26 1.08557 25 119.87181 24 180.57350

H 27 1.08494 8 121.82433 7 178.76924

H 28 1.09425 9 108.21734 6 218.35796

H 28 1.08562 9 107.91066 6 334.59701

H 29 1.09416 28 108.81246 9 181.10830

H 29 1.09417 28 110.56413 9 299.92183

H 29 1.09290 28 112.29053 9 61.38589

H 30 1.08803 12 107.37890 11 177.51881

H 30 1.09519 12 108.68123 11 293.79962

H 31 1.09428 30 109.13462 12 178.67984

H 31 1.09339 30 112.16913 12 298.37294

H 31 1.09432 30 110.61745 12 59.64833

H 32 1.08400 15 108.53324 2 33.30109

H 32 1.09350 15 107.49907 2 148.64184

H 33 1.09414 32 109.11291 15 180.37790

H 33 1.09353 32 111.82011 15 300.10705

H 33 1.09421 32 110.68279 15 61.32918

H 39 1.08503 37 119.84540 36 0.44349

H 40 1.08541 39 119.81178 37 180.97864

H 42 1.08540 41 120.06267 40 180.47699

H 43 1.08580 42 119.70565 41 179.88610

H 44 1.08505 38 119.85102 36 0.47209

H 45 1.08535 44 119.81083 38 180.96994

H 47 1.08548 46 120.05962 45 180.47019

H 48 1.08586 47 119.71899 46 179.84040

C 41 1.49824 40 120.02644 39 177.27253

C 46 1.49822 45 120.13101 44 177.18383

F 83 1.34342 41 111.96181 40 31.45348

F 83 1.34356 41 111.96058 40 152.21440

F 83 1.34739 41 111.71715 40 271.83579

F 84 1.34732 46 111.71356 45 270.12858

F 84 1.34387 46 111.90928 45 150.58341

F 84 1.34319 46 112.01399 45 29.82419

Computational data of the oxidized cation **1**$^{+}$ (**1**$^{2+\bullet}$):

Sum of electronic and thermal Free Energies in Hartree/particle = –2570.986269

Imaginary frequencies: none

Coordinates (Z-matrix):

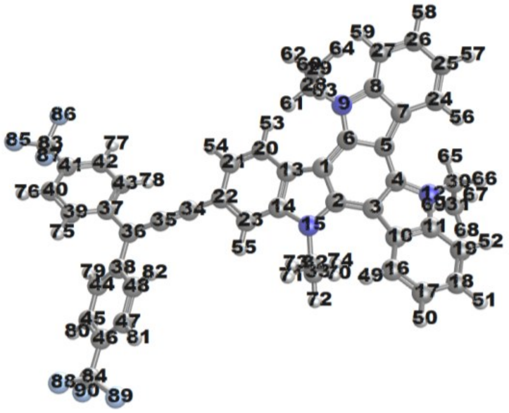

C

C 1 1.42501

C 2 1.42961 1 121.11579

C 3 1.43924 2 116.82918 1 344.84057

C 4 1.39843 3 120.93653 2 23.99792

C 1 1.42189 2 117.39529 3 355.40443

C 5 1.44785 4 135.41510 3 155.82888

C 7 1.41104 5 105.87659 4 189.98406

N 6 1.34438 1 129.66206 2 198.05323

C 3 1.42313 2 136.96584 1 169.18038

C 10 1.42935 3 106.05740 2 167.57689

N 4 1.37414 3 108.97616 2 193.39468

C 1 1.42123 2 107.09687 3 170.31895

C 13 1.42694 1 105.78501 2 3.36664

N 2 1.36115 1 109.29011 3 186.57597

C 10 1.41265 3 135.38791 2 341.68523

C 16 1.37800 10 119.11410 3 181.22205

C 17 1.41189 16 121.15103 10 0.98836

C 18 1.38436 17 121.44484 16 2.36827

C 13 1.41285 1 135.45181 2 189.29753

C 20 1.37269 13 119.48655 1 178.09238

C 21 1.42639 20 121.07387 13 359.04828

C 14 1.37686 13 122.79241 1 179.58976

C 7 1.39986 5 135.72955 4 4.93892

C 24 1.39284 7 119.20455 5 181.50896

C 25 1.39457 24 121.11393 7 0.91131

C 8 1.38397 7 123.07003 5 180.53696

C 9 1.46333 6 126.81022 1 19.77393

C 28 1.52400 9 112.05355 6 97.05537

C 12 1.46456 4 125.70722 3 152.26669

C 30 1.52282 12 112.33183 4 260.63915

C 15 1.46276 2 128.00160 1 162.69108

C 32 1.52242 15 112.23429 2 272.59765

C 22 1.38646 21 120.00275 20 178.85065

C 2 7.07130 1 73.49745 3 188.77203

C 2 8.40555 1 75.78110 3 188.53166

C 36 1.45746 35 118.75006 34 113.78340

C 36 1.45754 35 118.77147 34 293.89623

C 37 1.40790 36 120.77580 35 210.50750

C 39 1.38625 37 120.24011 36 178.16544

C 40 1.39349 39 119.78195 37 0.53269

C 41 1.39683 40 120.68180 39 359.04845

C 42 1.38278 41 119.70663 40 0.10547

C 38 1.40805 36 120.84225 35 209.72334

C 44 1.38618 38 120.24242 36 178.44725

C 45 1.39375 44 119.78868 38 0.50108

C 46 1.39660 45 120.67227 44 358.93281

C 47 1.38283 46 119.70033 45 0.24049
H 16 1.08371 10 121.35175 3 357.37039
H 17 1.08511 16 119.55731 10 179.49267
H 18 1.08554 17 119.25617 16 179.98665
H 19 1.08448 18 120.96739 17 176.23873
H 20 1.08319 13 121.49094 1 0.71638
H 21 1.08441 20 120.20306 13 179.89092
H 23 1.08417 14 122.65736 13 181.07116
H 24 1.08219 7 121.92167 5 359.81569
H 25 1.08511 24 119.17550 7 180.53745
H 26 1.08532 25 119.89767 24 180.64265
H 27 1.08478 8 121.97239 7 178.62083
H 28 1.09394 9 108.12317 6 220.68461
H 28 1.08577 9 107.93153 6 337.06769
H 29 1.09396 28 108.56780 9 181.24493
H 29 1.09396 28 110.59163 9 299.97594
H 29 1.09250 28 112.41311 9 61.61607
H 30 1.08774 12 107.25942 4 20.05591
H 30 1.09491 12 108.71352 4 136.55277
H 31 1.09406 30 108.91704 12 178.43516
H 31 1.09305 30 112.35331 12 298.07104
H 31 1.09412 30 110.58730 12 59.50873
H 32 1.08394 15 108.44179 2 34.44355
H 32 1.09321 15 107.34167 2 149.45280
H 33 1.09390 32 108.99319 15 180.54291
H 33 1.09371 32 111.88822 15 300.17784
H 33 1.09401 32 110.81509 15 61.52765
H 39 1.08458 37 120.16238 36 1.12908
H 40 1.08441 39 119.97323 37 181.36879
H 42 1.08483 41 120.18874 40 180.34436
H 43 1.08531 42 119.99099 41 179.44513
H 44 1.08457 38 120.18193 36 1.28112
H 45 1.08455 44 119.96817 38 181.28250

H 47 1.08473 46 120.18698 45 180.44802

H 48 1.08536 47 119.97616 46 179.44810

C 41 1.50558 40 119.95685 39 177.19636

C 46 1.50559 45 119.84567 44 176.94501

F 83 1.33911 41 111.82356 40 26.07313

F 83 1.34054 41 111.46660 40 146.85249

F 83 1.34275 41 110.88728 40 266.23542

F 84 1.34275 46 110.87075 45 268.55898

F 84 1.34041 46 111.54234 45 149.09848

F 84 1.33923 46 111.75686 45 28.30678

Computational data of the neutral radical **1•**:

Sum of electronic and thermal Free Energies in Hartree/particle = –2571.365862

Imaginary frequencies: none

Coordinates (Z-matrix):

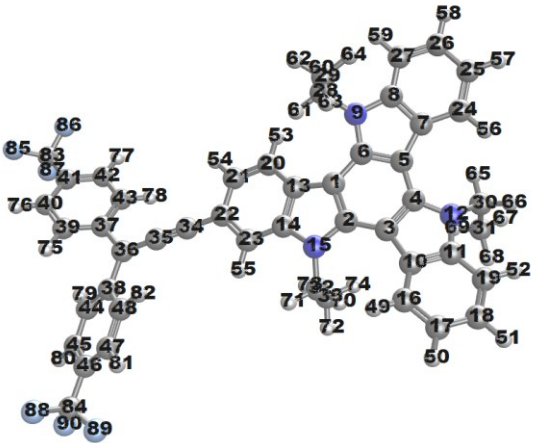

C

C 1 1.42168

C 2 1.41714 1 121.44149

C 3 1.41837 2 116.69793 1 349.10027

C 4 1.41667 3 121.90107 2 18.88456

C 5 1.41182 4 117.50544 3 349.61931

C 5 1.44997 4 135.21145 3 158.76399

C 7 1.41648 5 105.85066 4 188.46510

N 6 1.38333 5 109.72560 4 173.32084

C 3 1.45193 2 137.13998 1 174.15490

C 10 1.41782 3 106.03234 2 168.19902

N 4 1.38225 3 109.50282 2 191.98788

C 1 1.43785 2 106.90065 3 171.42370

C 13 1.41953 1 106.11644 2 3.07201

N 2 1.38535 1 108.98506 3 184.90140

C 10 1.40284 3 135.95102 2 341.04415

C 16 1.39007 10 119.82503 3 182.33298

C 17 1.39978 16 121.05372 10 1.09078

C 18 1.38956 17 120.55829 16 2.43894

C 13 1.40667 1 135.51303 2 190.03233

C 20 1.38114 13 120.07214 1 176.53524

C 21 1.41762 20 121.16451 13 358.92180

C 14 1.38654 13 122.50620 1 181.28815

C 7 1.40327 5 135.96882 4 2.74173

C 24 1.38929 7 119.73316 5 183.02816

C 25 1.40038 24 121.07840 7 0.86429

C 26 1.38877 25 120.59395 24 1.59986

C 9 1.45998 6 124.64747 5 209.81436

C 28 1.52401 9 112.96831 6 84.13443

C 12 1.45869 4 124.67457 3 148.11886

C 30 1.52414 12 112.81834 4 266.27409

C 15 1.45706 2 125.69054 1 154.70640

C 32 1.52292 15 112.82636 2 283.92970

C 22 1.40837 21 120.13755 20 179.35530

C 2 7.12101 1 73.58813 3 188.85179

C 2 8.47332 1 75.83696 3 188.79401

C 36 1.46312 35 118.47858 34 312.81653

C 36 1.46330 35 118.45418 34 132.80239

C 37 1.40869 36 121.85549 35 208.19159

C 39 1.38666 37 121.01178 36 178.25611

C 40 1.39487 39 120.14356 37 359.86027

C 41 1.39592 40 119.78060 39 359.44282

C 42 1.38495 41 120.02522 40 0.35797

C 38 1.40851 36 121.85207 35 208.63192

C 44 1.38680 38 121.01757 36 178.25596

C 45 1.39469 44 120.13490 38 359.86928

C 46 1.39606 45 119.78217 44 359.42934

C 47 1.38487 46 120.02992 45 0.36595

H 16 1.08463 10 121.40274 3 358.83626

H 17 1.08630 16 119.25790 10 179.91521

H 18 1.08631 17 119.88415 16 180.29915

H 19 1.08603 18 120.84823 17 176.58677

H 20 1.08431 13 121.21360 1 359.45545

H 21 1.08534 20 119.97163 13 179.94024

H 23 1.08471 14 122.01524 13 180.21501

H 24 1.08370 7 121.45753 5 0.78035

H 25 1.08637 24 119.26618 7 180.16471

H 26 1.08635 25 119.87613 24 180.31624

H 27 1.08595 26 120.73577 25 177.88135

H 28 1.09494 9 108.19905 6 207.55507

H 28 1.08677 9 108.30608 6 323.17526

H 29 1.09494 28 109.62263 9 180.86627

H 29 1.09479 28 110.50758 9 300.04920

H 29 1.09383 28 111.73437 9 60.87463

H 30 1.08801 12 107.60757 4 26.30730

H 30 1.09617 12 108.91936 4 142.55311

H 31 1.09486 30 109.52353 12 178.61027

H 31 1.09367 30 111.94246 12 298.56015

H 31 1.09475 30 110.47761 12 59.42909

H 32 1.08522 15 108.81392 2 46.18933

H 32 1.09358 15 107.54056 2 161.07683

H 33 1.09483 32 109.84176 15 180.53629

H 33 1.09452 32 111.31117 15 300.46569

H 33 1.09474 32 110.61330 15 61.17122

H 39 1.08507 37 119.81515 36 0.39465

H 40 1.08552 39 119.79391 37 180.98740

H 42 1.08550 41 120.05303 40 180.50663

H 43 1.08583 42 119.71369 41 179.96303

H 44 1.08509 38 119.81020 36 0.38600

H 45 1.08544 44 119.80237 38 180.98169

H 47 1.08558 46 120.05085 45 180.48664

H 48 1.08585 47 119.72208 46 179.93148

C 41 1.49725 40 120.05253 39 177.25785

C 46 1.49726 45 120.16540 44 177.21044

F 83 1.34402 41 112.00109 40 31.36444

F 83 1.34384 41 112.01970 40 152.14210

F 83 1.34814 41 111.81273 40 271.77456

F 84 1.34809 46 111.81042 45 270.00338

F 84 1.34412 46 111.96872 45 150.44639

F 84 1.34379 46 112.04983 45 29.67338

Computational data of the neutral HPE-dimer **1-1**:

Sum of electronic and thermal Free Energies in Hartree/particle = –5142.714727

Imaginary frequencies: none

Coordinates (Z-matrix):

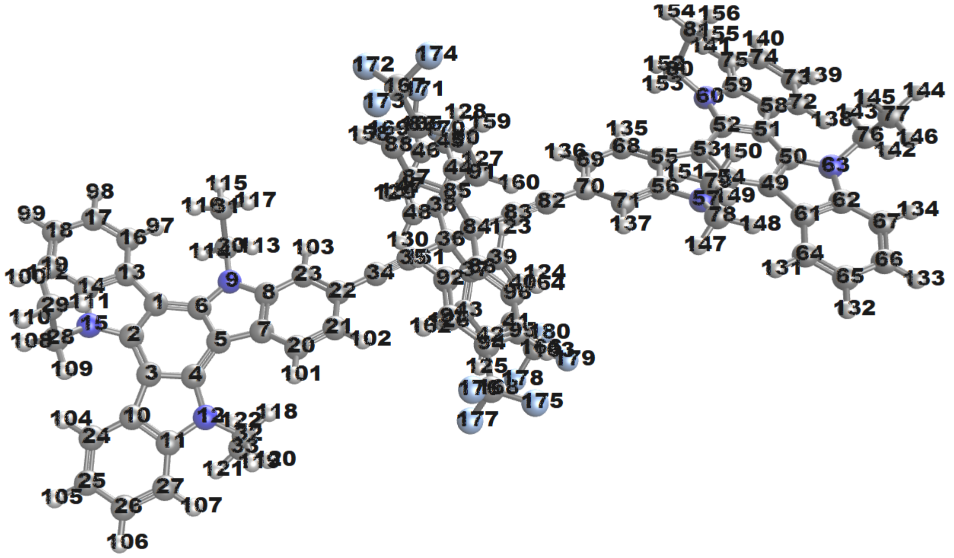

C

C 1 1.42062

C 2 1.41392 1 122.81008

C 3 1.40875 2 117.62890 1 13.34287

C 4 1.41493 3 121.03744 2 0.74926

C 5 1.42544 4 118.28171 3 346.34836

C 5 1.43914 4 134.58716 3 167.10230

C 7 1.41682 5 106.01128 4 173.53069

N 8 1.37875 7 109.28097 5 2.28449

C 3 1.44861 2 135.19177 1 201.06892

C 10 1.41566 3 105.79073 2 175.11158

N 4 1.38563 3 109.62060 2 183.29459

C 1 1.46111 2 105.65846 3 172.63697

C 13 1.41882 1 105.99967 2 2.91486

N 2 1.38015 1 110.15103 3 183.51299

C 13 1.40476 1 137.32026 2 183.51128

C 16 1.38940 13 120.46344 1 180.36544

C 17 1.39875 16 121.30220 13 359.95296

C 18 1.38715 17 120.06803 16 359.57661

C 7 1.40415 5 135.66162 4 346.56284

C 20 1.38441 7 119.98566 5 182.60285

C 21 1.41216 20 121.04435 7 0.95486

C 8 1.39060 7 122.49790 5 180.41771

C 10 1.40312 3 135.77722 2 1.54346

C 24 1.38949 10 119.67219 3 176.41738

C 25 1.40022 24 120.99715 10 359.10567

C 26 1.38935 25 120.65903 24 358.25148

C 15 1.46018 2 124.24266 1 212.01733

C 28 1.52407 15 112.90895 2 89.10917

C 9 1.44905 8 121.63246 7 182.66414

C 30 1.52260 9 113.43337 8 273.53986

C 12 1.45960 4 124.92880 3 150.21399

C 32 1.52372 12 112.91724 4 280.41407

C 22 1.42485 21 119.86495 20 180.54384

C 2 9.42283 1 45.68799 3 25.60978

C 4 9.53567 1 89.08355 2 195.36203

C 36 1.54702 35 108.85115 34 311.56910

C 36 1.54694 35 107.56545 34 68.12451

C 37 1.39891 36 120.46166 35 155.00370

C 39 1.38914 37 120.86778 36 179.30467

C 40 1.39333 39 119.94751 37 0.40762

C 41 1.39176 40 119.84258 39 0.00486

C 42 1.39039 41 119.89600 40 359.81910

C 38 1.39565 36 124.76745 35 132.77022

C 44 1.39326 38 120.77482 36 177.99814

C 45 1.38987 44 120.26333 38 359.95368

C 46 1.39448 45 119.66318 44 0.00377

C 47 1.38664 46 119.77696 45 0.04154

C 40 9.10386 39 94.23747 37 234.88441

C 49 1.41730 40 124.98161 39 263.98039

C 50 1.41362 49 121.90456 40 33.16884

C 51 1.41557 50 117.84434 49 8.47742

C 52 1.41423 51 121.71669 50 351.65562

C 49 1.41185 40 35.99555 39 354.19834

C 53 1.44661 52 135.73040 51 192.23284

C 55 1.41875 53 106.11319 52 182.74487

N 56 1.38408 55 109.71308 53 356.64486

C 51 1.45097 50 135.80281 49 192.16035

C 58 1.41648 51 106.01852 50 182.86044

N 59 1.38607 58 109.73851 51 356.75669

C 49 1.45112 40 113.82104 39 130.86699

C 61 1.41633 49 106.00056 40 147.76862

N 50 1.38627 49 109.36346 40 216.84653

C 61 1.40261 49 135.72636 40 334.04714

C 64 1.39027 61 119.65448 49 178.03640

C 65 1.39986 64 121.02082 61 359.21266

C 66 1.38983 65 120.65764 64 357.74737

C 55 1.40342 53 135.76026 52 9.02907

C 68 1.38565 55 119.95112 53 178.31817

C 69 1.41110 68 121.18814 55 359.17253

C 56 1.38870 55 122.57673 53 178.32167

C 58 1.40279 51 135.70746 50 9.15139

C 72 1.39007 58 119.64906 51 178.03442

C 73 1.40008 72 121.02793 58 359.22798

C 74 1.38966 73 120.65925 72 357.71921

C 63 1.45889 50 124.85971 49 212.84347

C 76 1.52410 63 112.87782 50 89.46359

C 57 1.45936 56 120.97251 55 152.44068

C 78 1.52404 57 112.89275 56 299.72656

C 60 1.45894 59 120.94518 58 152.48299

C 80 1.52415 60 112.94195 59 300.65450

C 70 1.42487 69 119.91318 68 179.24101

C 7 9.05506 1 140.66330 2 206.19305

C 5 9.02905 1 125.99596 2 205.22120

C 84 1.54789 83 107.66424 82 46.27625

C 84 1.54576 83 108.75983 82 289.59666

C 85 1.39568 84 124.78382 83 134.01177

C 87 1.39311 85 120.78326 84 178.11308

C 88 1.38982 87 120.26818 85 359.91230

C 89 1.39447 88 119.65829 87 359.95106

C 90 1.38654 89 119.77641 88 0.13038

C 86 1.39913 84 120.43845 83 154.27819

C 92 1.38924 86 120.85340 84 179.10131

C 93 1.39371 92 119.95999 86 0.28502

C 94 1.39148 93 119.83637 92 0.11829

C 95 1.39075 94 119.89348 93 359.77421

H 16 1.07990 13 122.02531 1 0.68118

H 17 1.08640 16 118.95389 13 180.15577

H 18 1.08627 17 120.13738 16 180.03526

H 19 1.08599 18 120.91500 17 180.65349

H 20 1.08473 7 121.21190 5 359.48924

H 21 1.08564 20 119.96218 7 179.70901

H 23 1.08491 8 122.03466 7 179.16079

H 24 1.08446 10 121.29486 3 358.98068

H 25 1.08639 24 119.32144 10 179.92232

H 26 1.08638 25 119.86329 24 179.69411

H 27 1.08587 26 120.60544 25 182.42222

H 28 1.09558 15 108.62176 2 212.76875

H 28 1.08739 15 107.89479 2 328.70761

H 29 1.09494 28 109.53059 15 181.35280

H 29 1.09481 28 110.46465 15 300.46808

H 29 1.09368 28 111.92901 15 61.35075

H 30 1.09464 9 107.03412 8 35.26244

H 30 1.08994 9 107.76418 8 148.17473

H 31 1.09522 30 110.15954 9 180.10270

H 31 1.09394 30 111.30117 9 300.35571

H 31 1.09458 30 110.62551 9 60.59566

H 32 1.08625 12 108.71398 4 41.99909

H 32 1.09442 12 107.83588 4 157.35368

H 33 1.09498 32 109.75659 12 179.78338

H 33 1.09415 32 111.53461 12 299.76902

H 33 1.09485 32 110.55470 12 60.51975

H 39 1.08433 37 120.08555 36 358.66006

H 40 1.08540 39 119.83679 37 179.56865

H 42 1.08540 41 120.24331 40 179.25576

H 43 1.08480 42 119.33723 41 179.34887

H 44 1.08256 38 120.91815 36 357.97154

H 45 1.08540 44 119.55471 38 179.35529

H 47 1.08550 46 120.28195 45 179.64733

H 48 1.08602 47 119.24091 46 179.73377

H 64 1.08447 61 121.37239 49 0.62199

H 65 1.08635 64 119.30211 61 180.02907

H 66 1.08639 65 119.84813 64 179.57323

H 67 1.08606 66 120.78699 65 182.85278

H 68 1.08414 55 121.40978 53 0.67265

H 69 1.08571 68 119.81470 55 179.84819

H 71 1.08523 56 121.64657 55 181.68505

H 72 1.08449 58 121.35462 51 0.58170

H 73 1.08637 72 119.30584 58 180.02421

H 74 1.08639 73 119.84688 72 179.54918

H 75 1.08607 74 120.78335 73 182.84526

H 76 1.09581 63 108.52265 50 212.90544

H 76 1.08723 63 108.02762 50 328.85068

H 77 1.09495 76 109.65020 63 180.89077

H 77 1.09483 76 110.51189 63 300.12757

H 77 1.09394 76 111.79376 63 60.92238

H 78 1.09572 57 108.48130 56 63.23611

H 78 1.08711 57 107.99162 56 179.09793

H 79 1.09489 78 109.62197 57 180.94314

H 79 1.09478 78 110.51398 57 300.16252

H 79 1.09404 78 111.84494 57 60.99758

H 80 1.09582 60 108.54122 59 64.16242

H 80 1.08727 60 108.03182 59 180.10298

H 81 1.09495 80 109.63810 60 181.03277

H 81 1.09482 80 110.50863 60 300.27104

H 81 1.09388 80 111.80605 60 61.06673

H 87 1.08264 85 120.87461 84 358.23347

H 88 1.08534 87 119.55266 85 179.41535

H 90 1.08550 89 120.28047 88 179.64618

H 91 1.08589 90 119.23158 89 179.55937

H 92 1.08435 86 120.07303 84 358.39929

H 93 1.08563 92 119.81119 86 179.30753

H 95 1.08532 94 120.24429 93 179.29786

H 96 1.08494 95 119.34015 94 179.44611

C 46 1.49855 45 120.29615 44 182.42669

C 41 1.49865 40 120.02870 39 182.09126

C 89 1.49849 88 120.33137 87 182.37301

C 94 1.49864 93 119.87217 92 182.18314

F 165 1.34392 46 111.89733 45 210.76989

F 165 1.34727 46 111.71268 45 91.25812

F 165 1.34327 46 112.06311 45 331.52627

F 167 1.34326 89 112.07622 88 331.75523

F 167 1.34729 89 111.72648 88 91.51339

F 167 1.34400 89 111.88613 88 211.00720

F 168 1.34321 94 112.10146 93 205.90867

F 168 1.34392 94 111.95687 93 326.71571

F 168 1.34767 94 111.66337 93 86.18828

F 166 1.34373 41 112.02429 40 208.05569

F 166 1.34753 41 111.69569 40 88.44142

F 166 1.34363 41 112.02238 40 328.83110

Computational data of the two-electron oxidized HPE-dimer $\mathbf{1}^{+\bullet}$**-**$\mathbf{1}^{+\bullet}$:

Sum of electronic and thermal Free Energies in Hartree/particle = –5142.338102

Imaginary frequencies: none

Coordinates (Z-matrix):

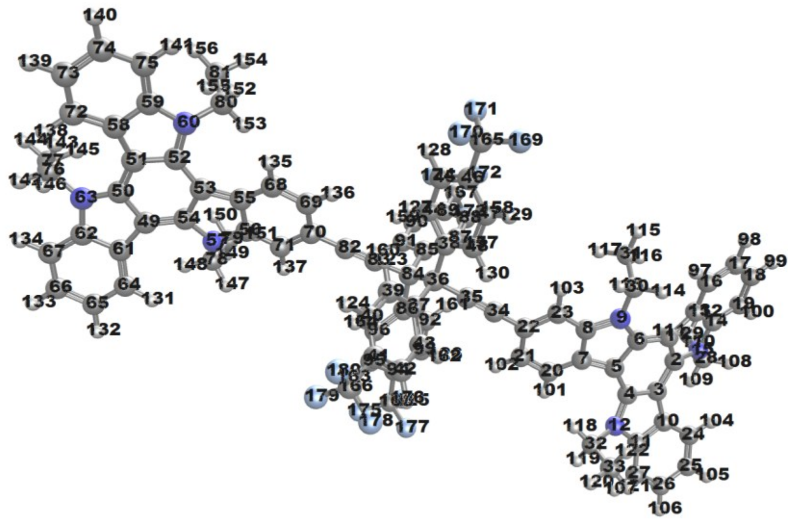

C

C 1 1.44792

C 2 1.42211 1 122.86536

C 3 1.40395 2 117.07475 1 15.40733

C 4 1.42073 3 120.81482 2 2.61455

C 1 1.39972 2 116.04677 3 342.59562

C 5 1.41821 4 134.62310 3 162.69589

C 7 1.42224 5 106.18994 4 173.39108

N 6 1.37608 1 131.45587 2 190.08574

C 3 1.44027 2 135.47374 1 204.52966

C 10 1.42005 3 105.50735 2 173.49794

N 4 1.37558 3 109.67800 2 185.16538

C 1 1.46395 2 105.52406 3 171.48141

C 13 1.41199 1 105.89166 2 4.24304

N 2 1.34802 1 109.29480 3 181.87823

C 13 1.39847 1 136.97810 2 186.20785

C 16 1.39412 13 119.92784 1 178.68746

C 17 1.39324 16 121.36564 13 359.71215

C 14 1.38652 13 123.72335 1 180.65946

C 7 1.41177 5 134.89875 4 347.35137

C 20 1.37635 7 119.50957 5 182.19896

C 21 1.41926 20 120.89625 7 0.74547

C 8 1.38641 7 122.20569 5 180.57263

C 10 1.40597 3 136.13136 2 359.57807

C 24 1.38433 10 119.27122 3 176.90769

C 25 1.40684 24 121.41071 10 359.29567

C 26 1.38279 25 120.83542 24 357.95599

C 15 1.46153 2 126.27789 1 203.56263

C 28 1.52408 15 111.92656 2 99.44588

C 9 1.45381 6 129.01346 1 347.44817

C 30 1.52111 9 113.44610 6 91.49470

C 12 1.46271 4 126.73389 3 156.64592

C 32 1.52330 12 112.46794 4 271.52435

C 22 1.41922 21 119.45166 20 179.94085

C 2 9.35753 1 47.15732 3 31.97207

C 4 9.49970 1 88.58388 2 200.28291

C 36 1.54726 35 108.67012 34 300.59721

C 36 1.54725 35 107.47584 34 57.20148

C 37 1.39867 36 120.16925 35 154.60662

C 39 1.38919 37 120.78200 36 178.88234

C 40 1.39312 39 119.94600 37 0.57306

C 41 1.39162 40 119.88331 39 0.03962

C 42 1.39027 41 119.90667 40 359.63159

C 38 1.39531 36 124.83911 35 131.36528

C 44 1.39333 38 120.70927 36 177.99384

C 45 1.38962 44 120.26568 38 0.00219

C 46 1.39434 45 119.70431 44 0.00431

C 47 1.38658 46 119.75901 45 359.99808

C 40 9.17054 39 92.63969 37 234.73632

C 49 1.45218 40 123.02278 39 262.20764

C 50 1.41403 49 121.94340 40 32.54807

C 51 1.41328 50 117.25026 49 10.66511

C 52 1.42518 51 121.81290 50 348.92468

C 49 1.38929 40 37.56949 39 356.26130

C 53 1.42436 52 135.60136 51 196.75526

C 55 1.42734 53 106.24483 52 183.06605

N 56 1.37683 55 109.96254 53 356.12274

C 51 1.44701 50 136.40082 49 191.65586

C 58 1.41838 51 105.73259 50 185.44590

N 52 1.37102 51 109.40100 50 172.78996

C 49 1.45079 40 116.19531 39 128.54289

C 61 1.40956 49 105.90863 40 147.90543

N 50 1.35407 49 108.30429 40 213.06411

C 61 1.39788 49 135.29795 40 334.36335

C 64 1.39411 61 119.17538 49 176.72638

C 65 1.39438 64 120.98359 61 359.08161

C 62 1.38500 61 122.82469 49 180.83995

C 55 1.41248 53 135.25257 52 8.45900

C 68 1.37564 55 119.52631 53 179.29513

C 69 1.42106 68 121.15238 55 359.30637

C 56 1.38854 55 122.28973 53 177.51054

C 58 1.40300 51 136.02561 50 12.27261

C 72 1.38759 58 119.27680 51 177.15636

C 73 1.40391 72 121.47152 58 359.00292

C 74 1.38480 73 120.71554 72 358.02728

C 63 1.45935 50 127.24132 49 203.03274

C 76 1.52391 63 111.97926 50 101.09389

C 57 1.46314 56 121.90513 55 155.20121

C 78 1.52280 57 112.61674 56 299.35347

C 60 1.46230 52 126.33952 51 206.01667

C 80 1.52366 60 112.24670 52 98.03496

C 70 1.41966 69 119.27874 68 179.12309

C 7 9.01881 1 140.54832 2 210.79070

C 5 8.97176 1 125.85595 2 209.72694

C 84 1.54752 83 107.23529 82 45.35341

C 84 1.54562 83 108.81233 82 288.71355

C 85 1.39507 84 124.68133 83 132.16419

C 87 1.39312 85 120.70704 84 177.92727

C 88 1.38955 87 120.24678 85 0.04584

C 89 1.39447 88 119.72199 87 359.89895

C 90 1.38639 89 119.76883 88 0.06560

C 86 1.39893 84 120.28993 83 153.63148

C 92 1.38920 86 120.79080 84 179.21273

C 93 1.39360 92 119.95892 86 0.32487

C 94 1.39121 93 119.86718 92 0.12160

C 95 1.39073 94 119.89418 93 359.73470

H 16 1.07968 13 122.29584 1 359.78408

H 17 1.08554 16 118.85841 13 180.27683

H 18 1.08539 17 120.16389 16 180.39561

H 19 1.08501 14 121.53750 13 179.27872

H 20 1.08408 7 121.18436 5 359.02498

H 21 1.08473 20 120.22699 7 179.36106

H 23 1.08387 8 122.33772 7 179.36545

H 24 1.08362 10 121.63081 3 359.29157

H 25 1.08578 24 119.23534 10 179.97054

H 26 1.08567 25 119.61086 24 179.30937

H 27 1.08510 26 120.92794 25 182.09444

H 28 1.09460 15 108.50100 2 223.08901

H 28 1.08642 15 107.68098 2 339.72860

H 29 1.09406 28 108.73287 15 181.26633

H 29 1.09410 28 110.46644 15 300.01408

H 29 1.09272 28 112.41793 15 61.50467

H 30 1.09365 9 106.69569 6 213.45542

H 30 1.08953 9 107.45232 6 325.87565

H 31 1.09462 30 109.74381 9 179.96003

H 31 1.09376 30 111.53067 9 300.03813

H 31 1.09440 30 110.79283 9 60.61062

H 32 1.08541 12 108.44898 4 32.60747

H 32 1.09393 12 107.80163 4 148.23537

H 33 1.09434 32 109.16840 12 179.51957

H 33 1.09337 32 111.91751 12 299.28970

H 33 1.09440 32 110.62096 12 60.47771

H 39 1.08454 37 120.15815 36 358.06238

H 40 1.08529 39 119.82487 37 179.60606

H 42 1.08529 41 120.25001 40 179.18666

H 43 1.08481 42 119.19896 41 179.68558

H 44 1.08244 38 121.11277 36 357.73364

H 45 1.08525 44 119.55182 38 179.32570

H 47 1.08543 46 120.29948 45 179.68658

H 48 1.08618 47 119.14666 46 179.99043

H 64 1.08376 61 121.59928 49 359.50607

H 65 1.08539 64 119.26721 61 180.02691

H 66 1.08559 65 119.86226 64 179.98270

H 67 1.08502 62 121.81989 61 180.11234

H 68 1.08343 55 121.40200 53 1.95388

H 69 1.08478 68 120.09936 55 180.14851

H 71 1.08421 56 121.87138 55 181.64240

H 72 1.08371 58 121.70482 51 359.93485

H 73 1.08580 72 119.12850 58 180.02170

H 74 1.08558 73 119.71187 72 180.03332

H 75 1.08525 74 120.95894 73 183.30092

H 76 1.09508 63 108.41132 50 224.59145

H 76 1.08579 63 107.84977 50 341.18291

H 77 1.09410 76 108.87568 63 180.82345

H 77 1.09408 76 110.51013 63 299.69074

H 77 1.09304 76 112.34425 63 61.07423

H 78 1.09484 57 108.35868 56 63.14951

H 78 1.08694 57 107.74968 56 179.00559

H 79 1.09434 78 109.25292 57 181.07048

H 79 1.09439 78 110.60763 57 300.13525

H 79 1.09378 78 112.11239 57 61.32637

H 80 1.09484 60 108.44762 52 221.74837

H 80 1.08651 60 107.80047 52 338.17665

H 81 1.09423 80 108.95162 60 181.15090

H 81 1.09428 80 110.51952 60 300.04890

H 81 1.09284 80 112.25506 60 61.36909

H 87 1.08269 85 121.06902 84 357.79630

H 88 1.08518 87 119.54953 85 179.45988

H 90 1.08542 89 120.29024 88 179.71112

H 91 1.08601 90 119.16536 89 179.96506

H 92 1.08457 86 120.14000 84 358.26648

H 93 1.08557 92 119.81281 86 179.17731

H 95 1.08516 94 120.24858 93 179.25089

H 96 1.08503 95 119.20392 94 179.54449

C 46 1.49942 45 120.25972 44 182.39863

C 41 1.49961 40 120.05012 39 182.14155

C 89 1.49951 88 120.36389 87 182.25054

C 94 1.49955 93 119.83543 92 182.09393

F 165 1.34322 46 111.83803 45 210.75862

F 165 1.34670 46 111.60456 45 91.23210

F 165 1.34275 46 111.99870 45 331.51885

F 167 1.34258 89 112.04057 88 333.03472

F 167 1.34663 89 111.60672 88 92.81866

F 167 1.34332 89 111.78651 88 212.28650

F 168 1.34238 94 112.05542 93 205.58490

F 168 1.34340 94 111.87956 93 326.41909

F 168 1.34719 94 111.55100 93 85.84810

F 166 1.34286 41 111.97054 40 208.55711

F 166 1.34717 41 111.55106 40 88.97124

F 166 1.34298 41 111.97894 40 329.39363

Computational data of the neutral carbinol **2-OH**:

Sum of electronic and thermal Free Energies in Hartree/particle = –2171.947084

Imaginary frequencies: none

Coordinates (Z-matrix):

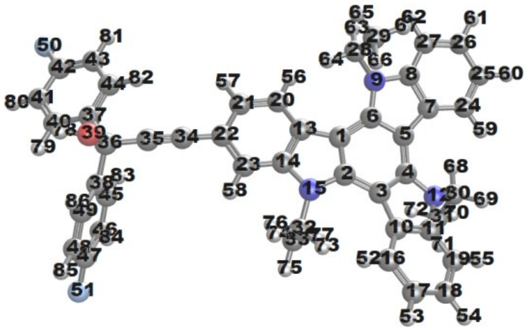

C

C 1 1.41976

C 2 1.41827 1 121.47959

C 3 1.41731 2 116.72587 1 349.15598

C 4 1.41601 3 121.87389 2 18.63998

C 5 1.41246 4 117.53316 3 349.77653

C 5 1.45001 4 135.17330 3 159.00057

C 7 1.41647 5 105.86061 4 188.38167

N 6 1.38424 5 109.67483 4 173.42842

C 3 1.45186 2 137.14263 1 173.70786

C 10 1.41810 3 106.04872 2 168.65924

N 4 1.38382 3 109.54749 2 191.58770

C 1 1.44198 2 106.85663 3 171.73674

C 13 1.41717 1 106.04252 2 2.94042

N 2 1.38595 1 109.02021 3 184.73372

C 10 1.40299 3 135.94021 2 341.51511

C 16 1.39007 10 119.83471 3 182.27697

C 17 1.39986 16 121.04881 10 1.07450

C 18 1.38957 17 120.55601 16 2.47971

C 13 1.40413 1 135.64900 2 189.83468

C 20 1.38450 13 120.01877 1 176.52454

C 21 1.41134 20 121.15553 13 358.85805

C 14 1.38967 13 122.45574 1 181.38840

C 7 1.40317 5 135.95147 4 2.66227

C 24 1.38950 7 119.74767 5 183.05314

C 25 1.40022 24 121.06286 7 0.87361

C 26 1.38899 25 120.58708 24 1.57212

C 9 1.45977 6 124.58455 5 210.02056

C 28 1.52405 9 112.98257 6 83.62468

C 12 1.45870 4 124.53211 3 147.52330

C 30 1.52415 12 112.86802 4 266.86406

C 15 1.45692 2 125.69982 1 154.57421

C 32 1.52305 15 112.84806 2 283.32771

C 22 1.42537 21 120.26428 20 179.34226

C 2 7.11400 1 73.86245 3 188.52583

C 2 8.53529 1 76.59855 3 188.56192

C 36 1.53339 35 111.81858 34 230.19184

C 36 1.53684 35 107.37185 34 353.13816

O 36 1.42916 35 106.07114 34 111.85863

C 37 1.39913 36 119.52774 35 161.89058

C 40 1.39077 37 121.00835 36 174.72951

C 41 1.38685 40 118.57081 37 0.24323

C 42 1.38660 41 121.96547 40 359.81744

C 43 1.39095 42 118.66988 41 359.86057

C 38 1.39857 36 120.35859 35 287.89637

C 45 1.38909 38 120.81834 36 178.32204

C 46 1.38887 45 118.60570 38 359.89326

C 47 1.38481 46 121.98331 45 359.78190

C 38 1.39318 36 120.44229 35 105.60808

F 42 1.34177 41 118.99180 40 179.57224

F 47 1.34314 46 118.88002 45 179.81535

H 16 1.08467 10 121.39086 3 358.80781

H 17 1.08633 16 119.26192 10 179.92030

H 18 1.08635 17 119.88253 16 180.33150

H 19 1.08613 18 120.83981 17 176.59651

H 20 1.08435 13 121.29446 1 359.44032

H 21 1.08555 20 119.81675 13 179.89907

H 23 1.08475 14 121.89007 13 180.27106

H 24 1.08375 7 121.43911 5 0.81243

H 25 1.08641 24 119.27205 7 180.15942

H 26 1.08637 25 119.88626 24 180.28332

H 27 1.08597 26 120.72877 25 177.86418

H 28 1.09500 9 108.17720 6 207.01638

H 28 1.08671 9 108.32875 6 322.63523

H 29 1.09497 28 109.65045 9 180.82130

H 29 1.09480 28 110.49890 9 300.00141

H 29 1.09389 28 111.72048 9 60.81614

H 30 1.08808 12 107.61888 4 26.94303

H 30 1.09614 12 108.89667 4 143.15268

H 31 1.09491 30 109.55597 12 178.67240

H 31 1.09370 30 111.90922 12 298.63424

H 31 1.09477 30 110.48094 12 59.47173

H 32 1.08524 15 108.81143 2 45.52019

H 32 1.09377 15 107.57389 2 160.46786

H 33 1.09487 32 109.84939 15 180.49802

H 33 1.09450 32 111.32550 15 300.42697

H 33 1.09478 32 110.60384 15 61.12906

H 39 0.96992 36 106.70698 35 167.39908

H 40 1.08683 37 119.99279 36 354.13645

H 41 1.08515 40 121.52533 37 179.76396

H 43 1.08524 42 119.82593 41 179.81812

H 44 1.08597 43 119.50873 42 180.03581

H 45 1.08678 38 119.83422 36 358.22457

H 46 1.08527 45 121.56703 38 179.80864

H 48 1.08535 47 119.86556 46 180.23589

H 49 1.08488 38 119.25627 36 1.23852

Computational data of the monocation of carbinol **2-OH** (**2-OH**$^{+\bullet}$):

Sum of electronic and thermal Free Energies in Hartree/particle = –2171.762176

Imaginary frequencies: none

Coordinates (Z-matrix):

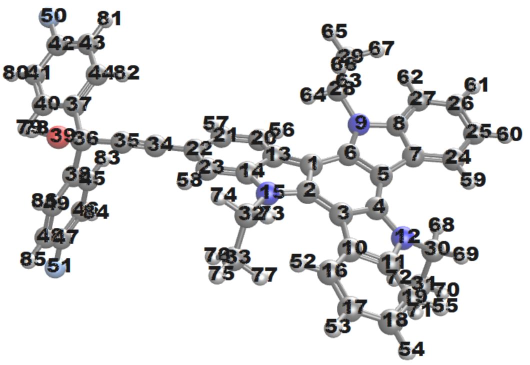

C

C 1 1.45795

C 2 1.40019 1 120.75015

C 3 1.44595 2 116.01918 1 358.65680

C 4 1.42230 3 122.87793 2 17.35701

C 5 1.40396 4 117.15617 3 344.30569

C 5 1.43981 4 135.32635 3 154.98797

C 7 1.41983 5 105.50624 4 186.66266

N 6 1.37598 5 109.62324 4 174.81501

C 3 1.46424 2 137.58004 1 191.32222

C 10 1.41221 3 105.87783 2 163.99876

N 4 1.34871 3 109.36597 2 195.34097

C 1 1.41710 2 106.92700 3 165.07331

C 13 1.42261 1 106.22395 2 6.77069

N 2 1.37489 1 107.41528 3 186.43849

C 10 1.39871 3 137.02504 2 342.11151

C 16 1.39383 10 119.94090 3 181.19656

C 17 1.39365 16 121.38458 10 0.29735

C 11 1.38695 10 123.72399 3 179.43978

C 13 1.41261 1 134.98683 2 192.77355

C 20 1.37577 13 119.55230 1 178.25015

C 21 1.41982 20 120.93120 13 359.30885

C 14 1.38509 13 122.26996 1 178.90167

C 7 1.40607 5 136.09101 4 0.52450

C 24 1.38431 7 119.27302 5 183.23115

C 25 1.40670 24 121.38690 7 0.72554

C 26 1.38296 25 120.84560 24 1.98117

C 9 1.46227 6 126.97126 5 202.55206

C 28 1.52330 9 112.49765 6 88.58420

C 12 1.46134 4 126.23087 3 156.53454

C 30 1.52406 12 111.96868 4 260.07993

C 15 1.45377 2 128.98668 1 184.47687

C 32 1.52123 15 113.40298 2 269.03202

C 22 1.41726 21 119.77444 20 179.23100

C 2 7.09580 1 72.96073 3 193.36285

C 2 8.52172 1 75.59094 3 193.62163

C 36 1.53130 35 111.87443 34 224.49907

C 36 1.53725 35 106.35179 34 347.39966

O 36 1.42527 35 105.64241 34 105.72089

C 37 1.39896 36 119.46402 35 161.12258

C 40 1.39049 37 120.90682 36 174.63279

C 41 1.38703 40 118.59765 37 0.24434

C 42 1.38676 41 121.99390 40 359.78619

C 43 1.39072 42 118.64965 41 359.86653

C 38 1.39858 36 120.51106 35 285.85389

C 45 1.38901 38 120.71511 36 178.17530

C 46 1.38881 45 118.60485 38 359.86026

C 47 1.38548 46 122.02740 45 359.76868

C 48 1.39276 47 118.66933 46 0.00589

F 42 1.34065 41 118.98722 40 179.56782

F 47 1.34170 46 118.87615 45 179.79746

H 16 1.07973 10 122.28313 3 0.12195

H 17 1.08557 16 118.84792 10 179.75044

H 18 1.08543 17 120.16100 16 179.61413

H 19 1.08502 11 121.45913 10 180.67400

H 20 1.08403 13 121.15460 1 1.58852

H 21 1.08444 20 120.18646 13 180.55669

H 23 1.08382 14 122.39867 13 180.83678

H 24 1.08370 7 121.62814 5 0.85184

H 25 1.08581 24 119.24494 7 180.02139

H 26 1.08570 25 119.61675 24 180.59963

H 27 1.08503 26 120.90215 25 177.79744

H 28 1.09416 9 107.72706 6 211.86927

H 28 1.08534 9 108.49094 6 327.40800

H 29 1.09436 28 109.20194 9 180.29157

H 29 1.09438 28 110.63028 9 299.34222

H 29 1.09340 28 111.89654 9 60.49098

H 30 1.08662 12 107.65208 4 19.81155

H 30 1.09474 12 108.54600 4 136.45147

H 31 1.09411 30 108.74562 12 178.46244

H 31 1.09269 30 112.41891 12 298.23399

H 31 1.09410 30 110.46372 12 59.69632

H 32 1.08963 15 107.47955 2 34.64689

H 32 1.09357 15 106.70136 2 147.12445

H 33 1.09462 32 109.75508 15 179.78480

H 33 1.09425 32 110.79696 15 299.10731

H 33 1.09377 32 111.53293 15 59.68683

H 39 0.96997 36 106.71093 35 167.80313

H 40 1.08670 37 120.06349 36 354.10273

H 41 1.08506 40 121.50130 37 179.80397

H 43 1.08514 42 119.83562 41 179.87720

H 44 1.08615 43 119.36002 42 180.14973

H 45 1.08665 38 119.94795 36 357.85895

H 46 1.08516 45 121.56291 38 179.67001

H 48 1.08524 47 119.84993 46 180.14424

H 49 1.08483 48 120.01266 47 180.59573

Computational data of the dication of carbinol **2-OH** (**2-OH**$^{2+\bullet\bullet}$):

Sum of electronic and thermal Free Energies in Hartree/particle = –2171.540518

Imaginary frequencies: none

Coordinates (Z-matrix):

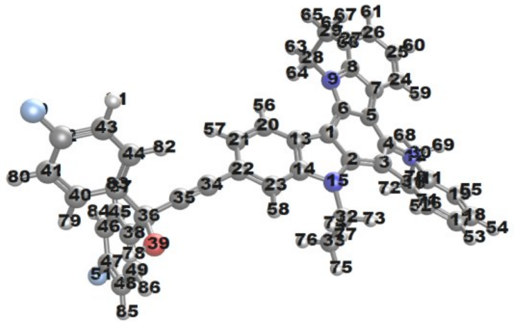

C

C 1 1.45113

C 2 1.41566 1 120.99865

C 3 1.44755 2 116.32420 1 344.80149

C 4 1.40758 3 121.13591 2 25.91579

C 1 1.41685 2 117.48905 3 353.75678

C 5 1.43622 4 135.46792 3 154.66162

C 7 1.41611 5 105.81488 4 189.24582

N 6 1.34519 1 130.05252 2 198.71098

C 3 1.43057 2 137.29315 1 174.12244

C 10 1.42439 3 105.93544 2 162.84041

N 4 1.35661 3 108.88907 2 197.53760

C 1 1.41584 2 107.05187 3 170.52677

C 13 1.42345 1 105.97211 2 3.15332

N 2 1.35315 1 108.55654 3 186.29131

C 10 1.40726 3 135.53350 2 336.37772

C 16 1.38312 10 118.99800 3 182.15109

C 17 1.40661 16 121.28810 10 1.21882

C 18 1.38815 17 121.25827 16 2.08077

C 13 1.41331 1 135.40516 2 189.40725

C 20 1.37464 13 119.33850 1 177.54033

C 21 1.42100 20 121.27555 13 359.01597

C 14 1.37780 13 122.95030 1 180.21173

C 7 1.40430 5 135.67447 4 3.96625

C 24 1.38858 7 119.01985 5 181.72702

C 25 1.39890 24 121.17597 7 0.81336

C 8 1.38447 7 122.98076 5 180.44760

C 9 1.46550 6 127.32757 1 19.36260

C 28 1.52386 9 111.94344 6 97.45797

C 12 1.46621 4 126.45419 3 156.27622

C 30 1.52342 12 112.03161 4 255.48363

C 15 1.46330 2 128.19240 1 164.60961

C 32 1.52207 15 111.99954 2 273.93116

C 22 1.40864 21 119.81040 20 179.28292

C 2 7.08029 1 73.26146 3 189.09512

C 2 8.51227 1 75.65584 3 189.51197

C 36 1.52981 35 111.80236 34 230.44628

C 36 1.53788 35 105.05374 34 353.12162

O 36 1.42215 35 105.79081 34 111.16958

C 37 1.39835 36 119.61903 35 158.22967

C 40 1.39051 37 120.82170 36 174.40935

C 41 1.38698 40 118.62246 37 0.20840

C 42 1.38713 41 122.01566 40 359.75518

C 43 1.39015 42 118.62468 41 359.89211

C 38 1.39923 36 120.58800 35 285.64937

C 45 1.38847 38 120.61769 36 178.29941

C 46 1.38926 45 118.60231 38 359.74190

C 47 1.38613 46 122.08059 45 359.76333

C 48 1.39189 47 118.66809 46 359.97942

F 42 1.33966 41 119.00213 40 179.55991

F 47 1.34000 46 118.85607 45 179.81146

H 16 1.08323 10 121.67245 3 358.16113

H 17 1.08492 16 119.35891 10 179.73439

H 18 1.08521 17 119.44765 16 179.71527

H 19 1.08404 18 120.96788 17 176.16655

H 20 1.08300 13 121.62290 1 0.44974

H 21 1.08416 20 120.01833 13 180.12750

H 23 1.08363 14 122.70552 13 180.66554

H 24 1.08200 7 121.91512 5 359.91091

H 25 1.08483 24 119.24808 7 180.43299

H 26 1.08510 25 119.71962 24 180.81474

H 27 1.08438 8 122.04424 7 178.51182

H 28 1.09355 9 107.81020 6 221.08474

H 28 1.08534 9 107.94347 6 337.37113

H 29 1.09374 28 108.35519 9 181.21419

H 29 1.09378 28 110.65460 9 299.84424

H 29 1.09230 28 112.50671 9 61.70911

H 30 1.08756 12 107.20252 4 14.68605

H 30 1.09461 12 108.47807 4 131.57025

H 31 1.09372 30 108.33814 12 177.73177

H 31 1.09233 30 112.69331 12 297.17563

H 31 1.09364 30 110.66769 12 59.08623

H 32 1.08315 15 108.44331 2 36.23173

H 32 1.09230 15 107.08555 2 150.98662

H 33 1.09365 32 108.84245 15 180.61574

H 33 1.09359 32 111.72615 15 300.06755

H 33 1.09395 32 110.94678 15 61.53289

H 39 0.97023 36 106.73869 35 168.69255

H 40 1.08644 37 120.10049 36 353.94050

H 41 1.08498 40 121.48451 37 179.83049

H 43 1.08503 42 119.84178 41 179.96406

H 44 1.08636 43 119.22020 42 180.31484

H 45 1.08652 38 120.02411 36 357.80726

H 46 1.08507 45 121.57432 38 179.49670

H 48 1.08510 47 119.84074 46 180.07296

H 49 1.08472 48 120.07002 47 180.53044

Computational data of the cation **2**$^{+}$:

Sum of electronic and thermal Free Energies in Hartree/particle = –2096.049621

Imaginary frequencies: none

Coordinates (Z-matrix):

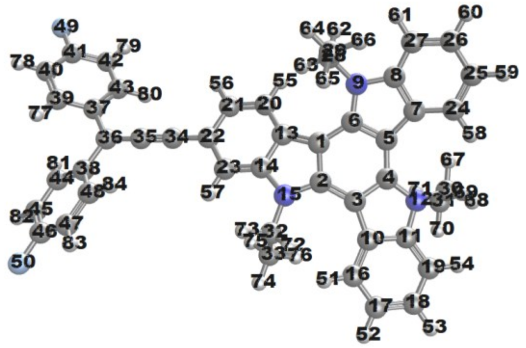

C

C 1 1.44245

C 2 1.41472 1 121.08750

C 3 1.42931 2 116.00707 1 359.03316

C 4 1.41966 3 123.00943 2 15.72970

C 5 1.40401 4 117.47725 3 345.30523

C 5 1.44696 4 135.24646 3 156.36773

C 7 1.41579 5 105.64951 4 186.13603

N 6 1.37791 5 109.85258 4 175.46243

C 3 1.46175 2 137.64893 1 189.93294

C 10 1.41609 3 105.85934 2 166.52260

N 4 1.36552 3 109.77028 2 192.44477

C 1 1.41278 2 107.20768 3 165.44343

C 13 1.43066 1 106.30166 2 6.75380

N 2 1.37744 1 107.70626 3 186.20227

C 10 1.40311 3 137.32318 2 345.24825

C 16 1.38984 10 120.24715 3 180.45289

C 17 1.39791 16 121.37283 10 0.20155

C 18 1.38770 17 120.13268 16 0.32339

C 13 1.41614 1 134.94310 2 193.10001

C 20 1.37101 13 119.76770 1 178.49154

C 21 1.42860 20 120.87851 13 359.15277

C 14 1.37420 13 122.41419 1 178.50889

C 7 1.40420 5 135.96299 4 359.74817

C 24 1.38758 7 119.52698 5 183.51481

C 25 1.40192 24 121.14443 7 0.84573

C 26 1.38724 25 120.71227 24 1.81864

C 9 1.46049 6 125.96097 5 206.80510

C 28 1.52339 9 112.70126 6 83.10892

C 12 1.46033 4 125.47114 3 152.81273

C 30 1.52397 12 112.51129 4 265.05089

C 15 1.45202 2 129.08485 1 185.58213

C 32 1.52167 15 113.40351 2 267.67284

C 22 1.38186 21 119.90524 20 179.10831

C 2 7.09141 1 72.62642 3 192.81069

C 2 8.42657 1 74.84986 3 192.71346

C 36 1.45715 35 118.80419 34 296.08450

C 36 1.45744 35 118.77051 34 116.03242

C 37 1.40973 36 121.09218 35 209.52902

C 39 1.38482 37 120.78605 36 178.00538

C 40 1.38983 39 118.62926 37 0.42062

C 41 1.39189 40 122.35882 39 359.23046

C 42 1.38233 41 118.54839 40 0.07415

C 38 1.40957 36 121.08033 35 210.22638

C 44 1.38487 38 120.78491 36 177.97990

C 45 1.38981 44 118.63072 38 0.45089

C 46 1.39188 45 122.35205 44 359.20254

C 47 1.38248 46 118.54877 45 0.05851

F 41 1.33137 40 118.87323 39 179.57697

F 46 1.33148 45 118.86846 44 179.56655

H 16 1.07986 10 122.13945 3 359.72016

H 17 1.08610 16 118.91556 10 179.76766

H 18 1.08593 17 120.11798 16 179.70775

H 19 1.08555 18 120.93666 17 178.97102

H 20 1.08400 13 120.91567 1 1.69455

H 21 1.08480 20 120.45293 13 180.35778

H 23 1.08437 14 122.67802 13 181.23836

H 24 1.08400 7 121.45459 5 1.05023

H 25 1.08612 24 119.27628 7 180.07037

H 26 1.08610 25 119.78735 24 180.38277

H 27 1.08557 26 120.72500 25 177.61752

H 28 1.09422 9 107.78367 6 206.21702

H 28 1.08621 9 108.75483 6 321.58555

H 29 1.09469 28 109.56120 9 180.11784

H 29 1.09468 28 110.59858 9 299.35495

H 29 1.09378 28 111.62364 9 60.21532

H 30 1.08698 12 107.76675 4 25.06565

H 30 1.09529 12 108.65920 4 141.36639

H 31 1.09459 30 109.20143 12 178.73664

H 31 1.09326 30 112.16451 12 298.63690

H 31 1.09452 30 110.47833 12 59.76795

H 32 1.08964 15 107.52091 2 33.10709

H 32 1.09412 15 106.87820 2 145.74285

H 33 1.09481 32 109.88607 15 179.35232

H 33 1.09444 32 110.78874 15 298.76886

H 33 1.09367 32 111.38548 15 59.27292

H 39 1.08477 37 119.97428 36 0.67688

H 40 1.08454 39 121.54161 37 181.17627

H 42 1.08458 41 119.77065 40 179.80057

H 43 1.08559 42 119.65188 41 179.39864

H 44 1.08480 38 119.93828 36 0.69351

H 45 1.08453 44 121.53125 38 181.20013

H 47 1.08462 46 119.76799 45 179.78311

H 48 1.08559 47 119.66440 46 179.43237

Computational data of the valence tautomer of the cation **2**$^{+}$ (**2**$^{+••}$):

Sum of electronic and thermal Free Energies in Hartree/particle = –2096.028272

Imaginary frequencies: none

Coordinates (Z-matrix):

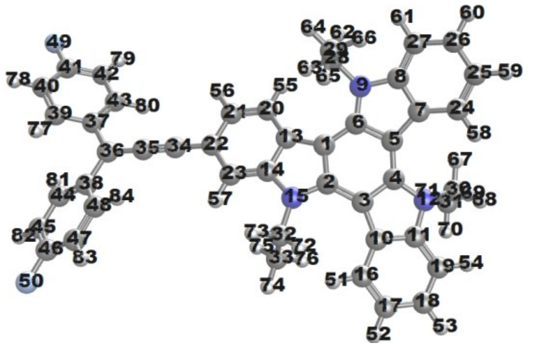

C

C 1 1.42971

C 2 1.44120 1 121.70795

C 3 1.42947 2 116.07306 1 1.27149

C 4 1.39709 3 122.06123 2 15.55256

C 1 1.40282 2 117.68375 3 343.62214

C 5 1.43950 4 135.11172 3 157.52526

C 7 1.41238 5 105.93292 4 183.18783

N 6 1.35844 1 130.65797 2 198.99123

C 3 1.43914 2 137.91785 1 188.78196

C 10 1.42988 3 106.01217 2 169.62183

N 11 1.37042 10 110.18853 3 1.30579

C 1 1.44261 2 107.08032 3 167.07503

C 13 1.41347 1 105.76650 2 5.97767

N 2 1.36486 1 107.87585 3 185.52154

C 10 1.41394 3 137.20480 2 349.81870

C 16 1.37897 10 120.08843 3 178.90354

C 17 1.40904 16 121.50441 10 0.06095

C 18 1.38024 17 120.63120 16 0.19845

C 13 1.40248 1 136.02923 2 193.37571

C 20 1.38389 13 119.68960 1 177.29268

C 21 1.41776 20 121.67467 13 358.79678

C 14 1.38742 13 123.26297 1 179.76129

C 7 1.40295 5 135.05234 4 356.54738

C 24 1.38991 7 119.18545 5 183.07078

C 25 1.39703 24 120.76682 7 1.04286

C 8 1.38495 7 122.41781 5 179.23127

C 9 1.46097 6 126.84550 1 19.47695

C 28 1.52317 9 112.23758 6 86.28551

C 12 1.46534 11 121.67307 10 206.35186

C 30 1.52309 12 112.66394 11 58.92845

C 15 1.45447 2 129.10073 1 186.75057

C 32 1.52246 15 112.84080 2 265.50994

C 22 1.40813 21 120.31951 20 179.16468

C 2 7.12172 1 73.75417 3 191.81423

C 2 8.47295 1 76.03878 3 191.66819

C 36 1.46314 35 118.54961 34 197.53636

C 36 1.46368 35 118.44619 34 17.55221

C 37 1.40888 36 121.73006 35 208.49860

C 39 1.38793 37 121.28118 36 178.28879

C 40 1.38793 39 118.84598 37 359.89203

C 41 1.38906 40 121.84428 39 359.50048

C 42 1.38624 41 118.74825 40 0.37002

C 38 1.40871 36 121.73094 35 209.27945

C 44 1.38800 38 121.27731 36 178.31585

C 45 1.38790 44 118.84616 38 359.91237

C 46 1.38901 45 121.84354 44 359.48764

C 47 1.38635 46 118.74278 45 0.35807

F 41 1.34052 40 119.10170 39 179.83185

F 46 1.34057 45 119.10253 44 179.81620

H 16 1.07838 10 122.18981 3 358.61180

H 17 1.08546 16 119.09422 10 179.83146

H 18 1.08568 17 119.67333 16 179.71097

H 19 1.08482 18 121.12896 17 179.14150

H 20 1.08402 13 121.64990 1 0.49899

H 21 1.08505 20 119.62430 13 179.81578

H 23 1.08410 14 122.36164 13 180.57055

H 24 1.08353 7 121.41352 5 0.62737

H 25 1.08519 24 119.46768 7 180.28263

H 26 1.08570 25 119.72663 24 180.38793

H 27 1.08474 8 121.92198 7 179.30577

H 28 1.09360 9 107.67220 6 209.42700

H 28 1.08477 9 108.54664 6 324.97743

H 29 1.09424 28 109.16963 9 179.79368

H 29 1.09431 28 110.64097 9 298.84255

H 29 1.09360 28 111.84221 9 60.04912

H 30 1.08724 12 107.76552 11 179.12565

H 30 1.09466 12 108.31622 11 295.08481

H 31 1.09437 30 109.09295 12 178.49124

H 31 1.09331 30 112.17978 12 298.24270

H 31 1.09443 30 110.60010 12 59.55410

H 32 1.08909 15 107.47811 2 31.15085

H 32 1.09308 15 106.65472 2 143.94741

H 33 1.09438 32 109.62211 15 178.75665

H 33 1.09380 32 110.72273 15 297.98958

H 33 1.09362 32 111.64153 15 58.55515

H 39 1.08521 37 119.75342 36 0.19875

H 40 1.08518 39 121.34123 37 180.71105

H 42 1.08522 41 119.77560 40 180.01647

H 43 1.08604 42 119.43413 41 179.64533

H 44 1.08526 38 119.74132 36 0.22624

H 45 1.08517 44 121.34180 38 180.72063

H 47 1.08525 46 119.77299 45 179.97465

H 48 1.08605 47 119.44441 46 179.61317

Computational data of the neutral radical **2•**:

Sum of electronic and thermal Free Energies in Hartree/particle = –2096.216001

Imaginary frequencies: none

Coordinates (Z-matrix):

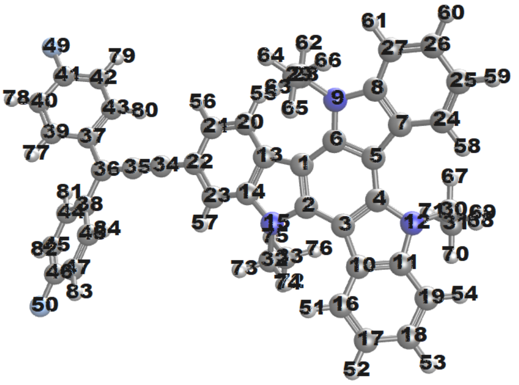

C

C 1 1.42094

C 2 1.41753 1 121.46814

C 3 1.41783 2 116.72925 1 349.10792

C 4 1.41623 3 121.87365 2 18.75912

C 5 1.41224 4 117.52048 3 349.72851

C 5 1.44997 4 135.18882 3 158.91229

C 7 1.41654 5 105.85921 4 188.44040

N 6 1.38390 5 109.69043 4 173.36267

C 3 1.45193 2 137.13034 1 173.89948

C 10 1.41797 3 106.04621 2 168.43009

N 4 1.38327 3 109.53261 2 191.78491

C 1 1.43957 2 106.88278 3 171.62525

C 13 1.41864 1 106.10997 2 2.97104

N 2 1.38571 1 108.99811 3 184.78869

C 10 1.40289 3 135.93186 2 341.28214

C 16 1.39010 10 119.82749 3 182.28366

C 17 1.39981 16 121.04765 10 1.08258

C 18 1.38961 17 120.55831 16 2.47020

C 13 1.40626 1 135.59688 2 189.99280

C 20 1.38170 13 120.10486 1 176.42244

C 21 1.41739 20 121.24488 13 358.87067

C 14 1.38742 13 122.55322 1 181.45180

C 7 1.40321 5 135.95258 4 2.73744

C 24 1.38940 7 119.74043 5 183.03095

C 25 1.40029 24 121.06825 7 0.87677

C 26 1.38890 25 120.59543 24 1.56751

C 9 1.45980 6 124.59952 5 209.88888

C 28 1.52406 9 112.98584 6 83.91626

C 12 1.45877 4 124.55215 3 147.63066

C 30 1.52413 12 112.84319 4 266.78078

C 15 1.45687 2 125.66725 1 154.55331

C 32 1.52308 15 112.85878 2 283.39302

C 22 1.40987 21 120.18297 20 179.48551

C 2 7.12484 1 73.65736 3 188.79459

C 2 8.47834 1 75.90840 3 188.76692

C 36 1.46358 35 118.50873 34 292.79957

C 36 1.46368 35 118.48956 34 112.71742

C 37 1.40902 36 121.90183 35 208.36674

C 39 1.38830 37 121.35232 36 178.32750

C 40 1.38773 39 118.87227 37 359.83691

C 41 1.38878 40 121.78493 39 359.48531

C 42 1.38659 41 118.76819 40 0.42652

C 38 1.40898 36 121.88971 35 208.78463

C 44 1.38829 38 121.35569 36 178.23318

C 45 1.38772 44 118.86766 38 359.85408

C 46 1.38878 45 121.78605 44 359.49726

C 47 1.38662 46 118.76996 45 0.40579

F 41 1.34187 40 119.11922 39 179.83289

F 46 1.34189 45 119.11962 44 179.83852

H 16 1.08468 10 121.38841 3 358.81940

H 17 1.08632 16 119.26346 10 179.92157

H 18 1.08633 17 119.88276 16 180.32485

H 19 1.08606 18 120.83928 17 176.59614

H 20 1.08438 13 121.22933 1 359.34564

H 21 1.08543 20 119.91828 13 179.94449

H 23 1.08477 14 121.94440 13 180.09030

H 24 1.08375 7 121.45161 5 0.78617

H 25 1.08639 24 119.26989 7 180.16206

H 26 1.08637 25 119.87963 24 180.28969

H 27 1.08600 26 120.71150 25 177.89296

H 28 1.09496 9 108.19212 6 207.34485

H 28 1.08673 9 108.30025 6 322.95762

H 29 1.09496 28 109.63633 9 180.85640

H 29 1.09480 28 110.49938 9 300.03642

H 29 1.09386 28 111.73690 9 60.85133

H 30 1.08806 12 107.61738 4 26.85100

H 30 1.09614 12 108.91297 4 143.07155

H 31 1.09489 30 109.54449 12 178.68164

H 31 1.09370 30 111.91504 12 298.63670

H 31 1.09477 30 110.48229 12 59.48736

H 32 1.08527 15 108.80666 2 45.59520

H 32 1.09373 15 107.56359 2 160.52805

H 33 1.09487 32 109.84856 15 180.51688

H 33 1.09450 32 111.32477 15 300.45374

H 33 1.09477 32 110.60411 15 61.15734

H 39 1.08529 37 119.72597 36 0.22506

H 40 1.08529 39 121.32388 37 180.68800

H 42 1.08534 41 119.78676 40 180.09618

H 43 1.08605 42 119.44418 41 179.72808

H 44 1.08528 38 119.71668 36 0.14974

H 45 1.08529 44 121.32601 38 180.71047

H 47 1.08534 46 119.78269 45 180.06048

H 48 1.08606 47 119.45003 46 179.71756

Computational data of the neutral dimer **2-2**:

Sum of electronic and thermal Free Energies in Hartree/particle = –4192.028252

Imaginary frequencies: none

Coordinates (Z-matrix):

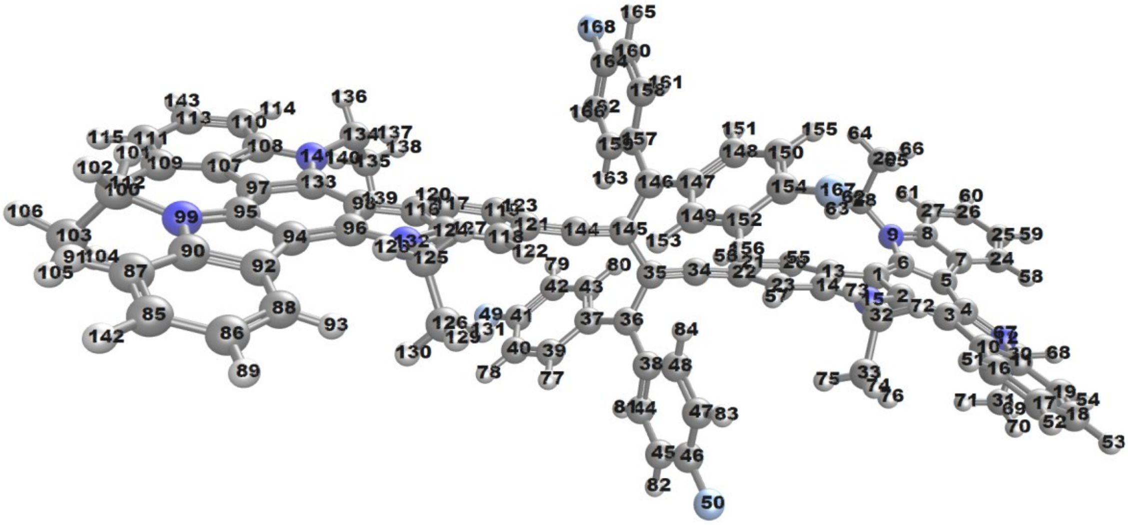

C

C 1 1.45487

C 2 1.40701 1 120.93136

C 3 1.43466 2 115.97180 1 359.09677

C 4 1.42270 3 123.10520 2 16.62280

C 5 1.40125 4 117.41209 3 344.36312

C 5 1.44553 4 135.25053 3 155.04236

C 7 1.41585 5 105.57632 4 186.52867

N 6 1.37392 5 109.96208 4 175.04008

C 3 1.46237 2 137.45430 1 191.66454

C 10 1.41432 3 105.80093 2 165.04977

N 4 1.35720 3 109.60164 2 193.81543

C 1 1.39638 2 107.21201 3 164.15841

C 13 1.43968 1 106.55377 2 7.36373

N 2 1.37274 1 107.62323 3 186.61995

C 10 1.40180 3 137.24688 2 343.89130

C 16 1.39033 10 120.09896 3 180.62944

C 17 1.39722 16 121.39770 10 0.22448

C 18 1.38828 17 120.18759 16 0.13370

C 13 1.42594 1 134.62993 2 192.93378

C 20 1.36044 13 119.84462 1 179.94207

C 21 1.44814 20 120.98554 13 358.41106

C 14 1.36220 13 122.65889 1 178.04259

C 7 1.40476 5 136.00787 4 0.21918

C 24 1.38652 7 119.42022 5 183.41775

C 25 1.40293 24 121.21549 7 0.79223

C 26 1.38617 25 120.76488 24 1.87020

C 9 1.46096 6 126.47528 5 205.09714

C 28 1.52329 9 112.58606 6 84.71375

C 12 1.46102 4 125.86342 3 155.12285

C 30 1.52378 12 112.28573 4 262.47467

C 15 1.45398 2 129.09233 1 188.51541

C 32 1.52173 15 113.57927 2 263.20430

C 22 1.34309 21 120.17466 20 178.36422

C 34 1.31230 22 174.83104 21 105.71158

C 35 1.48034 34 116.07546 22 342.99960

C 36 1.45290 35 116.98547 34 239.37468

C 36 1.44466 35 119.63407 34 56.32060

C 37 1.41226 36 121.46648 35 212.64642

C 39 1.38548 37 121.19040 36 177.92660

C 40 1.38993 39 118.86198 37 0.42388

C 41 1.38953 40 121.99360 39 359.79460

C 42 1.38604 41 118.66074 40 359.70793

C 38 1.41437 36 121.35225 35 207.15507

C 44 1.38526 38 121.41205 36 180.05352

C 45 1.38939 44 118.87379 38 359.98492

C 46 1.39108 45 121.88314 44 359.70212

C 47 1.38409 46 118.79444 45 0.06590

F 41 1.33664 40 119.02218 39 179.83677

F 46 1.33631 45 119.14268 44 179.95158

H 16 1.07981 10 122.22086 3 359.82396

H 17 1.08593 16 118.90331 10 179.74214

H 18 1.08575 17 120.10195 16 179.57656

H 19 1.08539 18 120.91808 17 179.03158

H 20 1.08391 13 120.61394 1 2.88464

H 21 1.08467 20 120.76765 13 180.24994

H 23 1.08344 14 122.84731 13 181.31449

H 24 1.08393 7 121.52534 5 1.02078

H 25 1.08600 24 119.25815 7 180.06942

H 26 1.08595 25 119.74558 24 180.46585

H 27 1.08548 26 120.75012 25 177.70771

H 28 1.09401 9 107.73525 6 207.86390

H 28 1.08609 9 108.76742 6 323.18939

H 29 1.09458 28 109.46512 9 180.13253

H 29 1.09461 28 110.61735 9 299.38779

H 29 1.09358 28 111.66688 9 60.29740

H 30 1.08681 12 107.71076 4 22.32348

H 30 1.09494 12 108.64814 4 138.74915

H 31 1.09437 30 109.02596 12 178.79675

H 31 1.09299 30 112.29758 12 298.64695

H 31 1.09438 30 110.48482 12 59.91266

H 32 1.08950 15 107.25352 2 28.40415

H 32 1.09483 15 106.87123 2 140.84161

H 33 1.09485 32 109.67040 15 178.51256

H 33 1.09465 32 111.17701 15 297.90460

H 33 1.09367 32 111.36815 15 58.59404

H 39 1.08539 37 119.59032 36 0.19068

H 40 1.08499 39 121.38374 37 181.05139

H 42 1.08506 41 119.80745 40 178.95322

H 43 1.08652 42 119.20816 41 178.13210

H 44 1.08504 38 119.56604 36 2.23711

H 45 1.08500 44 121.34899 38 181.00220

H 47 1.08512 46 119.70014 45 179.37756

H 48 1.08552 47 118.93672 46 178.77360

C 77 10.82248 39 116.55669 37 107.48541

C 85 1.39717 77 32.85690 39 222.91469

C 85 1.38840 77 90.74077 39 18.41122

C 86 1.38986 85 121.19786 77 30.92817

H 86 1.08591 85 119.77021 77 211.32179

C 87 1.39279 85 117.83620 77 343.84490

H 87 1.08457 85 120.47352 77 162.90886

C 88 1.40157 86 119.99436 85 0.33121

H 88 1.07970 86 118.02215 85 182.24615

C 92 1.45818 88 137.01905 86 180.34756

C 94 1.43721 92 106.56842 88 171.10746

C 94 1.39967 92 135.75632 88 347.26464

C 95 1.42715 94 122.67928 92 184.74578

C 96 1.45322 94 121.77538 92 176.21645

N 95 1.36303 94 108.22241 92 5.72028

C 99 1.45488 95 129.42528 94 169.02998

H 100 1.08843 99 107.93248 95 341.53907

H 100 1.09494 99 106.74672 95 228.04926

C 100 1.52367 99 113.14726 95 106.68689

H 103 1.09420 100 111.65287 99 303.50974

H 103 1.09343 100 111.29880 99 63.53868

H 103 1.09466 100 109.75216 99 182.99856

C 97 1.45231 95 135.93950 94 175.77392

C 107 1.41506 97 105.69598 95 171.83004

C 107 1.40660 97 137.23591 95 348.01925

C 108 1.39744 107 123.11470 97 183.21575

C 109 1.38563 107 120.10471 97 179.74165

H 109 1.07942 107 121.91455 97 357.58169

C 110 1.38396 108 117.91639 107 356.57038

H 110 1.08456 108 121.55795 107 177.44999

H 111 1.08595 109 119.02264 107 179.88795

C 98 1.40680 96 106.61301 94 188.28184

C 116 1.42622 98 136.06414 96 169.54552

C 116 2.46716 98 133.97695 96 349.87325

C 117 1.36092 116 120.63984 98 177.46866

H 117 1.07874 116 121.02227 98 355.21624

C 118 1.43837 116 89.03359 98 183.82769

H 118 1.08349 116 151.94590 98 0.82332

H 119 1.08406 117 120.36415 116 179.55552

C 118 1.36336 116 29.27477 98 6.94140

C 124 2.46874 118 97.75753 116 185.53841

C 125 1.52349 124 105.89340 118 296.38040

H 125 1.09540 124 85.11896 118 46.10834

H 125 1.08869 124 132.80372 118 153.14457

H 126 1.09460 125 109.70030 124 154.50996

H 126 1.09426 125 111.68207 124 274.95920

H 126 1.09362 125 111.53955 124 35.06191

N 96 1.37332 94 129.83220 92 357.39460

C 97 1.41542 95 117.55027 94 352.40592

C 108 2.46581 107 139.41074 97 356.05395

C 134 1.52464 108 105.69002 107 123.28840

H 134 1.09534 108 85.03347 107 232.64552

H 134 1.08969 108 133.22079 107 339.54532

H 135 1.09477 134 109.83171 108 156.09518

H 135 1.09468 134 111.91950 108 277.00389

H 135 1.09311 134 111.29209 108 36.67428

N 133 1.37339 97 109.13189 95 187.75258

H 85 1.08576 77 145.79927 39 172.20311

H 113 1.08583 110 119.65975 108 180.53190

C 121 1.34421 118 120.29927 116 177.45935

C 144 1.31157 121 173.99331 118 258.12730

C 145 1.48252 144 116.04422 121 11.44322

C 146 1.45481 145 116.60401 144 120.32056

C 147 1.41160 146 121.38968 145 145.84830

C 147 1.41124 146 120.65595 145 328.89810

C 148 1.38613 147 121.19956 146 183.88764

H 148 1.08535 147 119.56470 146 1.13360

C 149 1.38656 147 121.42212 146 176.62640

H 149 1.08670 147 119.42036 146 354.81519

C 150 1.38945 148 118.85577 147 359.49324

H 150 1.08499 148 121.37363 147 178.52464

H 152 1.08511 149 121.53285 147 178.97487

C 146 1.44352 145 120.04845 144 302.86725

C 157 1.41457 146 121.23076 145 153.53483

C 157 1.41536 146 121.18931 145 333.74332

C 158 1.38506 157 121.40892 146 179.61706

H 158 1.08508 157 119.54181 146 357.53049

C 159 1.38375 157 121.43865 146 180.60411

H 159 1.08539 157 119.62778 146 358.93023

C 160 1.38951 158 118.83783 157 0.03493

H 160 1.08499 158 121.37669 157 179.03226

H 162 1.08499 159 121.46354 157 178.98668

F 154 1.33811 150 119.05431 148 178.94253

F 164 1.33646 160 119.11091 158 180.01485

Computational data of the two-electron oxidized dimer of **2-2** (**$2^{+•}$-$2^{+•}$**):

Sum of electronic and thermal Free Energies in Hartree/particle = –4192.040282

Imaginary frequencies: none

Coordinates (Z-matrix):

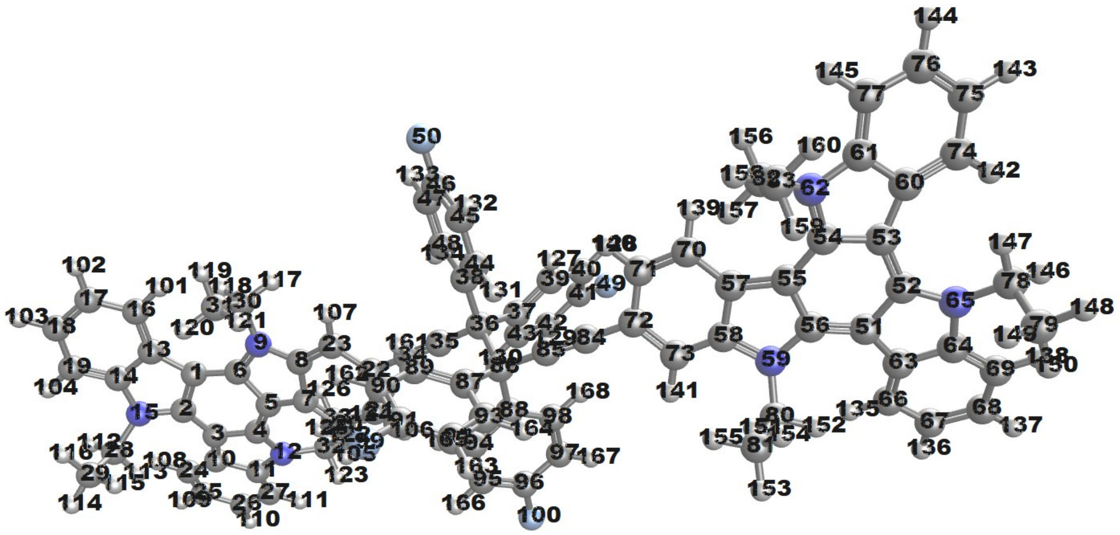

C

C 1 1.44734

C 2 1.42210 1 122.88165

C 3 1.40390 2 117.10396 1 15.36276

C 4 1.42109 3 120.79212 2 2.66741

C 1 1.39987 2 116.03771 3 342.61655

C 5 1.41751 4 134.64536 3 162.98366

C 7 1.42255 5 106.21612 4 173.03367

N 6 1.37582 1 131.47035 2 189.83118

C 3 1.44034 2 135.41873 1 204.63301

C 10 1.41994 3 105.50925 2 173.37939

N 4 1.37582 3 109.67024 2 185.24107

C 1 1.46418 2 105.51923 3 171.25743

C 13 1.41205 1 105.88597 2 4.18312

N 2 1.34833 1 109.30918 3 182.11377

C 13 1.39862 1 137.00913 2 186.22971

C 16 1.39398 13 119.94322 1 178.55423

C 17 1.39345 16 121.37419 13 359.66912

C 14 1.38672 13 123.73131 1 180.83659

C 7 1.41230 5 134.92787 4 347.12435

C 20 1.37595 7 119.51781 5 181.63026

C 21 1.41991 20 120.92400 7 0.82928

C 8 1.38597 7 122.21217 5 180.96421

C 10 1.40596 3 136.12101 2 359.44047

C 24 1.38434 10 119.27836 3 176.89432

C 25 1.40667 24 121.40262 10 359.29666

C 26 1.38287 25 120.83089 24 357.97508

C 15 1.46134 2 126.31966 1 203.30638

C 28 1.52410 15 112.01662 2 100.75775

C 9 1.45372 6 128.95126 1 348.33107

C 30 1.52103 9 113.34652 6 90.37868

C 12 1.46271 4 126.78512 3 156.79188

C 32 1.52341 12 112.48428 4 271.36368

C 22 1.41825 21 119.45258 20 180.95237

C 2 9.36847 1 47.08666 3 30.87900

C 4 9.49610 1 88.94093 2 199.08610

C 36 1.54676 35 108.27447 34 315.87339

C 36 1.54688 35 107.60543 34 72.70017

C 37 1.39933 36 120.17489 35 154.07222

C 39 1.39022 37 121.18637 36 178.84214

C 40 1.38674 39 118.73631 37 0.54690

C 41 1.38522 40 121.76495 39 359.98574

C 42 1.39149 41 118.68992 40 359.63926

C 38 1.39621 36 124.86218 35 132.10669

C 44 1.39435 38 121.10919 36 177.74277

C 45 1.38362 44 119.03831 38 359.99711

C 46 1.38755 45 121.58311 44 359.93394

C 47 1.38818 46 118.55323 45 359.97236

F 41 1.34153 40 119.06812 39 179.49366

F 46 1.34121 45 119.29121 44 179.86538

C 40 9.09334 39 93.84989 37 235.72336

C 51 1.44941 40 123.25944 39 262.20293

C 52 1.41641 51 121.97101 40 33.22599

C 53 1.41299 52 117.29491 51 10.40507

C 54 1.42377 53 121.75295 52 349.13773

C 51 1.38829 40 38.05526 39 356.20350

C 55 1.42396 54 135.68990 53 196.50748

C 57 1.42726 55 106.29774 54 183.44551

N 58 1.37821 57 109.91581 55 356.11737

C 53 1.44585 52 136.34788 51 191.99061

C 60 1.41912 53 105.74190 52 184.88616

N 54 1.37277 53 109.44140 52 173.37638

C 51 1.45179 40 115.63783 39 128.89355

C 63 1.40926 51 105.88483 40 147.55615

N 52 1.35443 51 108.32701 40 213.78770

C 63 1.39750 51 135.29381 40 334.18118

C 66 1.39431 63 119.15969 51 176.46745

C 67 1.39438 66 121.01353 63 359.06456

C 64 1.38553 63 122.82192 51 181.10105

C 57 1.41250 55 135.18145 54 9.02278

C 70 1.37578 57 119.54079 55 179.10249

C 71 1.42122 70 121.14883 57 359.30818

C 58 1.38735 57 122.29863 55 177.65005

C 60 1.40383 53 136.05792 52 11.45440

C 74 1.38681 60 119.29425 53 177.44974

C 75 1.40462 74 121.46583 60 359.08573

C 76 1.38428 75 120.73321 74 357.97703

C 65 1.45954 52 127.14193 51 203.11848

C 78 1.52401 65 112.03470 52 101.73208

C 59 1.46282 58 122.01513 57 155.41027

C 80 1.52288 59 112.60830 58 299.98690

C 62 1.46249 54 126.30750 53 206.55967

C 82 1.52356 62 112.22161 54 96.59782

C 72 1.41864 71 119.41307 70 179.29356

C 7 9.02691 1 142.01058 2 207.67847

C 5 8.99524 1 126.98122 2 207.80264

C 86 1.54688 85 107.28839 84 48.09874

C 86 1.54429 85 108.68481 84 291.09477

C 87 1.39615 86 124.66750 85 133.08107

C 89 1.39392 87 121.08398 86 177.58081

C 90 1.38380 89 119.02461 87 0.06265

C 91 1.38758 90 121.61191 89 359.83844

C 92 1.38805 91 118.55324 90 0.02560

C 88 1.39923 86 120.33506 85 153.05689

C 94 1.39075 88 121.18727 86 179.00793

C 95 1.38708 94 118.73814 88 0.34305

C 96 1.38517 95 121.75523 94 0.11705

C 97 1.39165 96 118.67305 95 359.63892

F 91 1.34099 90 119.26578 89 179.81376

F 96 1.34148 95 119.05864 94 179.56566

H 16 1.07974 13 122.28669 1 359.63213

H 17 1.08558 16 118.85429 13 180.25771

H 18 1.08543 17 120.17316 16 180.47924

H 19 1.08506 14 121.48930 13 179.12727

H 20 1.08407 7 121.14153 5 358.46794

H 21 1.08485 20 120.28314 7 179.61105

H 23 1.08396 8 122.36574 7 179.22353

H 24 1.08356 10 121.65231 3 359.21104

H 25 1.08578 24 119.23380 10 179.95168

H 26 1.08566 25 119.61588 24 179.32248

H 27 1.08509 26 120.90120 25 182.08946

H 28 1.09475 15 108.52983 2 224.38669

H 28 1.08659 15 107.63544 2 341.06917

H 29 1.09410 28 108.69535 15 181.50189

H 29 1.09405 28 110.49303 15 300.24029

H 29 1.09262 28 112.47560 15 61.75186

H 30 1.09359 9 106.73694 6 212.32022

H 30 1.08958 9 107.50784 6 324.75285

H 31 1.09457 30 109.79060 9 180.44601

H 31 1.09379 30 111.51363 9 300.53373

H 31 1.09435 30 110.75616 9 61.08354

H 32 1.08547 12 108.45066 4 32.43167

H 32 1.09405 12 107.80052 4 148.03231

H 33 1.09435 32 109.17050 12 179.33770

H 33 1.09336 32 111.93679 12 299.13077

H 33 1.09437 32 110.60502 12 60.31469

H 39 1.08466 37 119.98057 36 358.24061

H 40 1.08514 39 121.33817 37 179.85941

H 42 1.08510 41 119.97100 40 179.56263

H 43 1.08497 42 118.87182 41 179.98661

H 44 1.08259 38 120.97522 36 357.50376

H 45 1.08516 44 121.02951 38 179.62327

H 47 1.08513 46 120.00591 45 180.13185

H 48 1.08637 47 118.83611 46 180.37515

H 66 1.08383 63 121.61357 51 359.18815

H 67 1.08548 66 119.24322 63 180.01818

H 68 1.08558 67 119.86936 66 180.01762

H 69 1.08497 64 121.84779 63 179.99899

H 70 1.08356 57 121.31302 55 1.93874

H 71 1.08489 70 120.09290 57 180.25640

H 73 1.08440 58 121.92461 57 181.57140

H 74 1.08365 60 121.70447 53 0.23016

H 75 1.08580 74 119.14080 60 180.08308

H 76 1.08560 75 119.68700 74 179.97264

H 77 1.08522 76 120.97963 75 183.19837

H 78 1.09495 65 108.50050 52 225.30831

H 78 1.08578 65 107.78567 52 341.89331

H 79 1.09412 78 108.82101 65 181.21243

H 79 1.09403 78 110.50432 65 300.05781

H 79 1.09289 78 112.39165 65 61.47145

H 80 1.09482 59 108.38167 58 63.77569

H 80 1.08708 59 107.70943 58 179.66022

H 81 1.09433 80 109.24176 59 180.90025

H 81 1.09443 80 110.64189 59 299.99082

H 81 1.09378 80 112.07415 59 61.18376

H 82 1.09481 62 108.39905 54 220.20987

H 82 1.08649 62 107.90475 54 336.57499

H 83 1.09423 82 109.01949 62 181.09576

H 83 1.09431 82 110.48354 62 300.03471

H 83 1.09301 82 112.18043 62 61.29699

H 89 1.08306 87 120.93282 86 357.47374

H 90 1.08510 89 121.05362 87 179.78915

H 92 1.08509 91 120.01015 90 180.15752

H 93 1.08627 92 118.86298 91 180.35295

H 94 1.08482 88 119.98695 86 358.26522

H 95 1.08536 94 121.33332 88 179.37368

H 97 1.08513 96 119.96642 95 179.57524

H 98 1.08525 97 118.90478 96 179.91677

References

(1) Reger, D. L.; Wright, T. D.; Little, C. A.; Lamba, J. J.; Smith, M. D. Control of the stereochemical impact of the lone pair in lead(II) tris(pyrazolyl)methane complexes. Improved preparation of Na{B(3,5-$(CF_3)_2C_6H_3)_4$}. *Inorg. Chem.* **2001**, *40* (15), 3810–3814.

(2) Li, Y.; Josowicz, M.; Tolbert, L. M. Diferrocenyl molecular wires. The role of heteroatom linkers. *J. Am. Chem. Soc.* **2010**, *132* (30), 10374–10382.

(3) Krejčik, M.; Daněk, M.; Hartl, F. Simple construction of an infrared optically transparent thin-layer electrochemical cell. *J. Electroanal. Interfac. Electrochem.* **1991**, *317* (1-2), 179–187.

(4) M. J. Frisch, G. W. Trucks, H. B. Schlegel, G. E. Scuseria, M. A. Robb, J. R. Cheeseman, G. Scalmani, V. Barone, G. A. Petersson, H. Nakatsuji, X. Li, M. Caricato, A. V. Marenich, J. Bloino, B. G. Janesko, R. Gomperts, B. Mennucci, H. P. Hratchian, J. V. Ortiz, A. F. Izmaylov, J. L. Sonnenberg, D. Williams-Young, F. Ding, F. Lipparini, F. Egidi, J. Goings, B. Peng, A. Petrone, T. Henderson, D. Ranasinghe, V. G. Zakrzewski, J. Gao, N. Rega, G. Zheng, W. Liang, M. Hada, M. Ehara, K. Toyota, R. Fukuda, J. Hasegawa, M. Ishida, T. Nakajima, Y. Honda, O. Kitao, H. Nakai, T. Vreven, K. Throssell, J. A. Montgomery, Jr., J. E. Peralta, F. Ogliaro, M. J. Bearpark, J. J. Heyd, E. N. Brothers, K. N. Kudin, V. N. Staroverov, T. A. Keith, R. Kobayashi, J. Normand, K. Raghavachari, A. P. Rendell, J. C. Burant, S. S. Iyengar, J. Tomasi, M. Cossi, J. M. Millam, M. Klene, C. Adamo, R. Cammi, J. W. Ochterski, R. L. Martin, K. Morokuma, O. Farkas, J. B. Foresman, D. J. Fox. Gaussian 16, Revision C.01,Gaussian, Inc., Wallingford CT, USA, 2016.

(5) McLean, A. D.; Chandler, G. S. Contracted Gaussian basis sets for molecular calculations. I. Second row atoms, Z =11–18. *J. Chem. Phys.* **1980**, *72* (10), 5639–5648.

(6) Perdew, J. P.; Burke, K.; Ernzerhof, M. Generalized Gradient Approximation Made Simple. *Phys. Rev. Lett.* **1996**, *77* (18), 3865–3868.

(7) Cossi, M.; Rega, N.; Scalmani, G.; Barone, V. Energies, structures, and electronic properties of molecules in solution with the C-PCM solvation model. *J. Comput. Chem.* **2003**, *24* (6), 669–681.

(8) Casper, L. A.; Wursthorn, L.; Geppert, M.; Roser, P.; Linseis, M.; Drescher, M.; Winter, R. F. 4-Ferrocenylphenyl-Substituted Tritylium Dyes with Open and Interlinked C^+Ar_2 Entities: Redox Behavior, Electrochromism, and a Quantitative Study of the Dimerization of Their Neutral Radicals. *Organometallics* **2020**, *39* (17), 3275–3289.

(9) Imai, N.; Noguchi, T.; Otera, J. Convenient Preparation of 3, 3-Diaryl-2-propen-1-ols for Synthesis of Cibenzoline and the Analogs. *Bull. Okayama Univ. Sci. A* **2003** (39), 47–55.

(10) Vogelsang, L.; Birk, T.; Paschke, F.; Bauer, A.; Enenkel, V.; Holz, L. M.; Fonin, M.; Winter, R. F. Ferrocenyl-Substituted Triazatruxenes: Synthesis, Electronic Properties, and the Impact of Ferrocenyl Residues on Directional On-Surface Switching on Ag(111). *Inorg. Chem.* **2023**, *62* (39), 16236–16249.

(11) Brookhart, M.; Grant, B.; Volpe, A. F. $[(3,5\text{-}(CF_3)_2C_6H_3)_4B]^-[H(OEt_2)_2]^+$: a convenient reagent for generation and stabilization of cationic, highly electrophilic organometallic complexes. *Organometallics* **1992**, *11* (11), 3920–3922.
